# Gata3 dosage governs primitive endoderm versus trophectoderm specification in embryonic stem cells

**DOI:** 10.1038/s44319-026-00860-y

**Published:** 2026-07-06

**Authors:** Yeejin Jang, Mijeong Kim, Joonhyuk Choi, Jonghwan Kim

**Affiliations:** 1 Department of Molecular Biosciences, The University of Texas at Austin, Austin, TX 78712 USA; 2 Department of Medicine, Penn-CHOP Lung Biology Institute, Perelman School of Medicine, University of Pennsylvania, Philadelphia, PA USA

**Keywords:** Chromatin, Transcription & Genomics, Development, Stem Cells & Regenerative Medicine

## Abstract

Transcription factor (TF) dosage represents an overlooked aspect of developmental regulation. While Gata3 has traditionally been viewed as a determinant of trophectoderm (TE), its potential role in primitive endoderm (PE) has remained unclear. Here, we demonstrate that Gata3 functions as a dosage-sensitive regulator directing mutually exclusive lineage programs in mouse embryonic stem (ES) cells. Low levels of Gata3 (Gata3-L) promote PE-like transcriptional states, while high levels (Gata3-H) drive TE identity by rapidly repressing pluripotency and inducing TE markers. Genome-wide binding analysis reveals a dose-dependent redistribution of Gata3 across enhancers, with chromatin engagement consistent with pioneer factor-like activity. Functional 3D blastoid assays combined with single-cell transcriptomics further establish that Gata3 dosage alone is sufficient to instruct the spatial segregation of PE- versus TE-like compartments. These findings redefine Gata3 not merely as a TE determinant but as a central dosage-sensitive switch in lineage specification. More broadly, our results position TF dosage as a fundamental regulatory parameter that integrates enhancer selection, chromatin engagement, and spatial patterning, providing new opportunities to refine stem cell-based models and engineer developmental outcomes.

## Synopsis

Gata3 directs distinct lineage trajectories in ES cells through dose-dependent transcriptional and chromatin regulation. Differential Gata3 expression establishes PE-like and TE-like states that drive lineage segregation and spatial organization in early embryo models.

- Gata3 functions as a dosage-sensitive regulator of lineage specification in mouse ES cells.
- Low Gata3 levels preferentially induce PE-associated programs.
- High Gata3 expression remodels chromatin and activates TE networks.
- Gata3 redistributes across lineage-associated enhancers in a dose-dependent manner.
- In 3D blastoid assays, modulation of Gata3 dosage is sufficient to drive spatial segregation of epiblast-, PE-, and TE-like compartments.

## Abstract (web summary)

Gata3 directs distinct lineage trajectories in ES cells through dose-dependent transcriptional and chromatin regulation. Differential Gata3 expression establishes PE-like and TE-like states that drive lineage segregation and spatial organization in early embryo models.

## Graphical abstract

**Figure:**
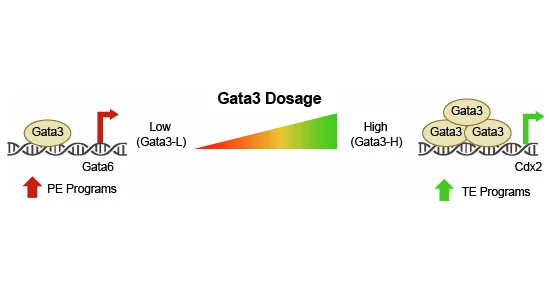

**Figure:**
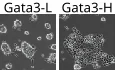

## Introduction

Early mammalian embryogenesis involves two critical steps of cell fate specification. First, the trophectoderm (TE), which contributes to the placenta, diverges from the inner cell mass (ICM). Second, the ICM further differentiates into the primitive endoderm (PE), giving rise to the yolk sac, and the epiblast (EPI), which develops into the embryo proper (Plusa and Piliszek, 2020; Rossant, 2008). These developmental transitions rely on intricate interactions between signaling pathways and transcription factor (TF) networks that guide proper lineage specification.

While numerous TFs regulate developmental decisions, the GATA family plays a pivotal role in both first and second lineage specification during early embryogenesis. Comprising six zinc finger proteins (GATA1-6), they bind the consensus sequence (A/T)GATA(A/G) and fall into two subfamilies—GATA1/2/3 and GATA4/5/6—each with distinct spatial and temporal expression patterns (Simon, 1995). In mouse, Gata2 and Gata3 are strongly linked to TE lineage development and are regulated by the Hippo signaling pathway, including nuclear translocation of Yap and Taz, which activates key TE markers such as Cdx2 (Zhu and Zernicka-Goetz, 2020). In contrast, Gata6 is essential for PE specification and is activated via the MAPK signaling cascade. Elevated Gata6 downregulates EPI markers such as Nanog and Sox2, while promoting the expression of PE markers, including Sox17, Gata4, and Sox7 (Schrode et al, 2014).

Ectopic expression of lineage-specifying TFs, singly or in combination, is a method utilized extensively in the context of induced pluripotency and direct lineage conversion (Liu et al, 2008; Takahashi and Yamanaka, 2006; Yu et al, 2007). For example, overexpression (OE) of Gata4 and Gata6 can reprogram mouse embryonic stem (ES) cells into extraembryonic endoderm (XEN)-like cells capable of contributing to PE-derived lineages (Fujikura et al, 2002). Similarly, Gata3 is a well-established master regulator of TE specification, with numerous studies supporting its role in trophoblast development (Assou et al, 2012; Home et al, 2009; Kim et al, 2023). Ectopic Gata3 in mouse ES cells activates trophoblast-associated genes (Ralston et al, 2010), and OE of GATA3 in human ES cells also triggers trophoblast-like differentiation (Xiao et al, 2020).

Importantly, although directing cells toward distinct fates in a dosage-dependent manner is relatively uncommon, certain TFs do exhibit this capacity, where variations in expression levels alter cell fate outcomes. Gata6, for instance, not only reprograms mouse ES cells to XEN-like cells but also shows dosage sensitivity in PE specification: reduced Gata6 levels bias cells toward an EPI over a PE fate (Schrode et al, 2014). Interestingly, while Gata3 is traditionally associated with TE identity, some studies have reported its ability to upregulate primitive endoderm-associated genes (Ralston et al, 2010; Xiao et al, 2020). This raises the intriguing possibility that Gata3, beyond its canonical role in TE development, may also participate in PE lineage specification, suggesting a broader and more nuanced role in early embryonic cell fate determination.

In this study, we investigated how ectopic expression of Gata3 in mouse ES cells directs both TE and PE-like fates in a dosage-dependent manner. Our results reveal that low levels of Gata3 induction preferentially promote PE lineage differentiation, while higher levels drive TE lineage specification. By mapping the genomic targets of Gata3 under different induction levels, we uncovered distinct transcriptional programs and target occupancy patterns underlying these fate choices. We further validated these findings in an in vitro blastoid model, demonstrating that Gata3 induction levels alone are sufficient to modulate spatial lineage specification, recapitulating key aspects of early embryonic patterning. Together, our findings expand the known functional repertoire of Gata3 beyond its classical role in TE development, highlighting its latent capacity to specify PE lineage and offering new insights into the dosage-sensitive regulatory mechanisms of GATA family TFs in early embryogenesis.

## Results

### Ectopic expression of Gata3 in mouse ES cells activates both TE and PE marker genes

While Gata3 is a well-characterized regulator of trophectoderm (TE) development (Assou et al, 2012; Home et al, 2009), its potential involvement in primitive endoderm (PE) differentiation has remained largely overlooked. Some reports have observed endodermal marker upregulation upon Gata3 OE in ES cells (Ralston et al, 2010; Xiao et al, 2020), but these findings were not further pursued mechanistically. We similarly observed this trend in our own system (Lee et al, 2024), which motivated us to systematically dissect Gata3’s role in PE specification. To address this, we generated stable mouse ES cell lines for Gata3 OE by employing an SBFB (Sleeping Beauty (SB) transposon-based inducible vector integrated with a metabolic biotinylation (FB)) system (Kim et al, 2024; Lee et al, 2024), enabling inducible expression of Gata3 upon Doxycycline (Dox) treatment. This system also allows biotinylation of the expressed factor via constitutive expression of BirA biotin ligase in mouse ES cells.

**Figure 1:**
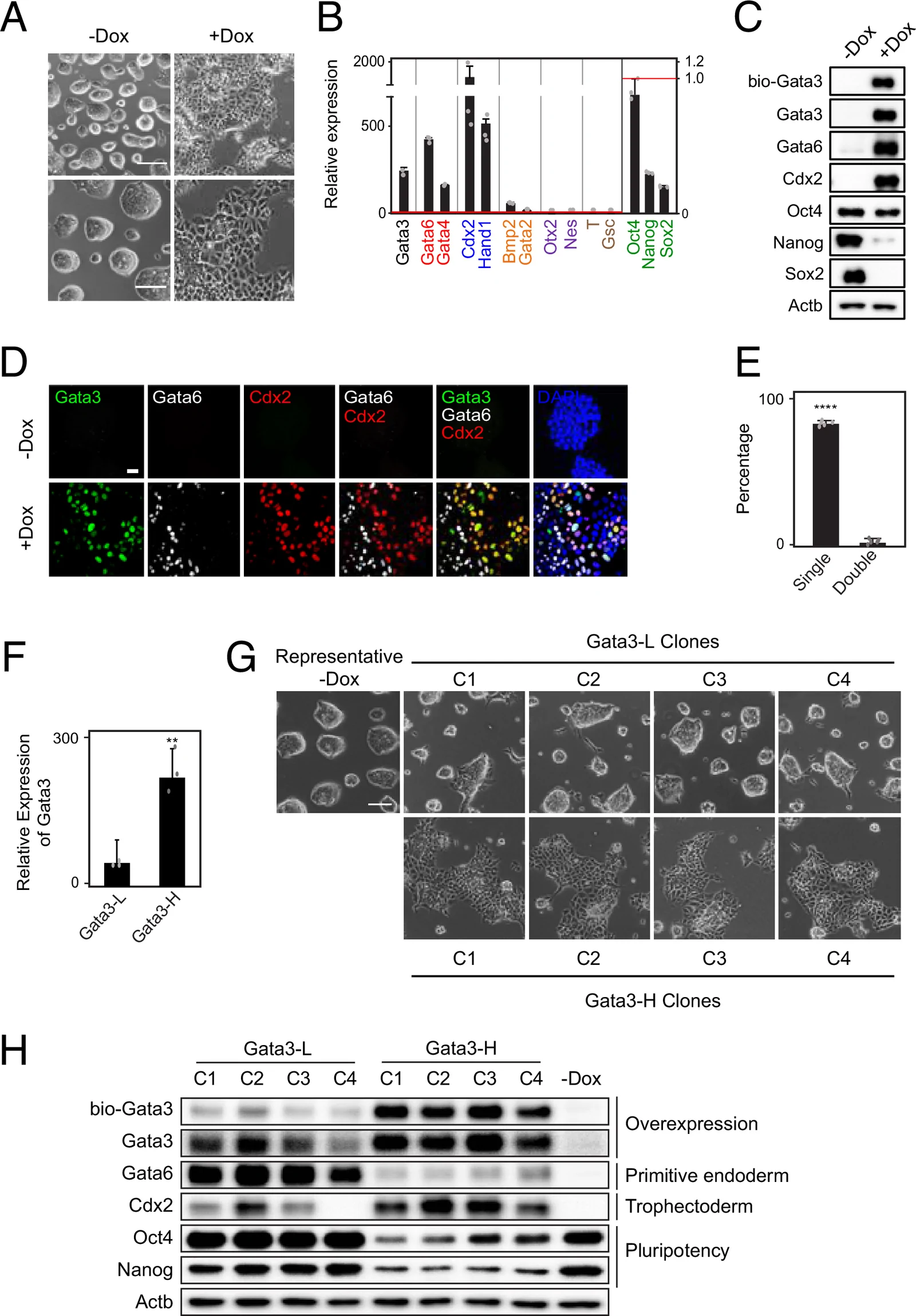
Gata3 overexpression initiates differentiation of mouse ES cells with bipotent lineage segregation. (**A**) Brightfield images of mouse ES cells overexpressing Gata3 (Gata3-OE) after treatment with 0.5 µg/mL of Dox for 2 days (+Dox). Untreated cells (−Dox) served as controls. Scale bars: 50 µm (top) and 100 µm (bottom). (**B**) Relative expression levels of multiple lineage and pluripotency marker genes in Gata3-OE cells after 2 days of 0.5 µg/mL Dox treatment. Relative expression levels of marker genes for primitive endoderm (PE, red), trophectoderm (TE, blue), mesoderm (orange), ectoderm (purple), mesendoderm (brown), and pluripotency (green) were quantified. The red line indicates the expression level in the -Dox control. Error bars indicate mean ± SD (*n* = 3 biological replicates). (**C**) Protein expression levels of PE (Gata6), TE (Cdx2), and pluripotency markers (Oct4, Nanog, and Sox2) in Gata3-OE cells (+Dox). Actb was used as a loading control, and −Dox samples served as controls. (**D**) Immunofluorescence analysis of Gata3 (green), Gata6 (gray), and Cdx2 (red) in Gata3-OE cells (+Dox), showing mutually exclusive expression of Gata6 and Cdx2. Cells were treated with 0.5 µg/mL Dox for 2 days; −Dox samples served as controls. Scale bars: 25 µm. (**E**) Quantification of cells expressing either Gata6 or Cdx2 signals (single) or both markers (double). *P* values were calculated using Student’s *t*-test (*P* = 7.96e-20). Error bars indicate mean ± SD (*n* = 8 biological replicates). (**F**) Relative expression of Gata3 in low-expressing (Gata3-L) and high-expressing (Gata3-H) clones. *P* values were calculated using Student’s *t*-test (*P* = 0.0012) across four biological replicates. Error bars indicate mean ± SD. (**G**) Brightfield images of Gata3-L and Gata3-H clones after induction with 0.5 µg/mL Dox for 2 days. Scale bar: 100 µm. (**H**) Western blot analysis showing expression levels of Gata3, PE (Gata6), TE (Cdx2), and pluripotency markers (Oct4 and Nanog) in Gata3-L and Gata3-H clones. Actb was used as a loading control, and −Dox samples served as controls. Source data are available online for this figure.

**Figure EV1:**
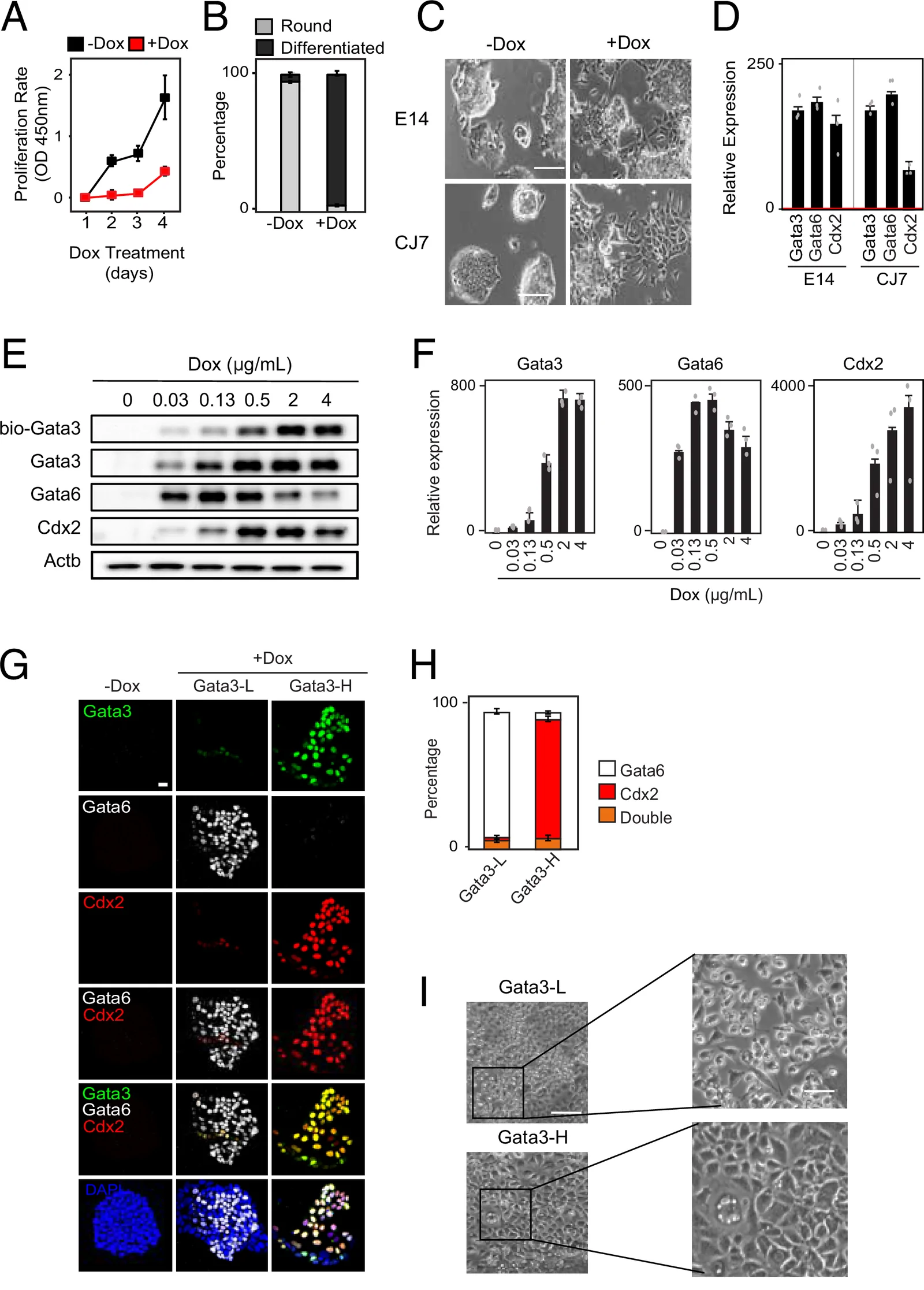
Early differentiation dynamics of mouse ES cells in response to Gata3 overexpression. (**A**) Growth curve of Gata3-OE ES cells (+Dox) compared to −Dox controls. Growth rate was measured on days 1, 2, 3, and 4 following treatments with 0.5 µg/mL Dox. Error bars indicate mean ± SD (*n* = 3 biological replicates). (**B**) Colony-forming assays of -Dox control and Gata3-OE (+Dox) cells. The bar graph represents the percentage of colonies with convex (round) or differentiated (flattened) morphology. Error bars indicate mean ± SD (*n* = 4 biological replicates). (**C**) Morphological changes in E14 and CJ7 mouse ES cells following Gata3 overexpression. Cells were treated with 0.5 µg/mL Dox for 2 days. Untreated cells (−Dox) were used as controls. Scale bar: 100 µm. (**D**) Expression of Gata3, Gata6 (PE), and Cdx2 (TE) in Gata3-OE E14 and CJ7 mouse ES cells. The red line indicates the expression level in the −Dox control. Error bars indicate mean ± SD (*n* = 3 biological replicates). (**E**) Protein and (**F**) relative mRNA expression levels of Gata3, Gata6 (PE), and Cdx2 (TE) in Gata3-OE cells treated with varying concentrations of Dox (0, 0.03, 0.13, 0.5, 2, 4 µg/mL) for 2 days. Dox with 0 µg/mL samples served as controls. Error bars indicate mean ± SD (*n* = 3 biological replicates). (**G**) Immunofluorescence analysis of Gata3 (green), Gata6, (gray) and Cdx2 (red) in Gata3-L and Gata3-H clones. Cells were treated with 0.5 µg/mL Dox for 2 days. −Dox samples were used as controls. Scale bars: 25 µm. (**H**) Quantification of the percentage of cells expressing only Gata6 (white), only Cdx2 (red), or both markers (orange, double) in Gata3-L (*n* = 8 biological replicates) and Gata3-H clones (*n* = 10 biological replicates). Error bars indicate mean ± SD. (**I**) Morphology of long-term induced Gata3-L and Gata3-H clones after 6 days of Dox treatment. Scale bars: 50 µm (left) and 100 µm (right). Source data are available online for this figure.

We first performed OE of Gata3 in mouse ES cells to test which key lineage markers are upregulated. Upon OE, ES cells exhibited marked morphological changes, including slower proliferation and a shift in colony morphology from compact, round colonies toward differentiated, flattened colonies, indicating a loss of self-renewal and the onset of differentiation (Figs. 1A and EV1A,B). Notably, Gata3 induction led to upregulation of both PE (Gata6 and Gata4) and TE (Cdx2 and Hand1) lineage markers, alongside downregulation of ES cell core self-renewal markers (Oct4, Nanog, and Sox2) at both the mRNA and protein levels, indicating a possible bipotent function (Fig. 1B,C). This bipotent response was further validated in additional ES cell lines (E14 and CJ7) (Fig. EV1C,D), highlighting that Gata3 OE induces dual lineages robustly across strains, independent of genetic background.

### The levels of ectopic Gata3 determine PE versus TE lineage choice from ES cells

Building on our observation of population-level upregulation of both PE and TE markers in Gata3-OE cells, we next asked whether these early lineage markers are co-expressed within individual cells or reflect mutually exclusive lineage commitments. To address this, we performed immunofluorescence (IF) staining on Gata3-OE cells after 2 days of Dox induction, monitoring the expression of Gata3, Gata6, and Cdx2 at the single-cell level. As shown in Fig. 1D, Gata6 and Cdx2 exhibited mutually exclusive expression patterns, with individual cells expressing either marker, but rarely both simultaneously. Quantitative analysis confirmed that ~95% of cells exhibited single-marker expression (Fig. 1E), indicating that Gata3 OE drives a mutually exclusive commitment to either the PE or TE lineage.

Notably, IF results also suggested that lineage choice correlates with the level of Gata3 induction: cells displaying higher Gata3 signal predominantly co-expressed Cdx2, while cells with lower Gata3 signal overlapped with Gata6 (Fig. 1D). This observation prompted us to test whether varying Gata3 induction levels directly influence lineage bias.

To investigate this, we treated Gata3-OE cells with a range of Dox concentrations for 2 days. Increasing Dox concentrations elevated ectopic Gata3 expression, along with increased Cdx2 levels at both the protein and mRNA levels (Fig. EV1E,F). In contrast, Gata6 expression was rapidly upregulated and peaked at lower Dox concentrations (0.13 and 0.5 µg/mL) but gradually declined at higher doses. These findings mirror the IF results (Fig. 1D), indicating that high levels of Gata3 induction reprogram cells toward TE lineage, while lower levels preferentially promote PE fate commitment.

### Stable Gata3-OE lines with different expression levels exhibit distinct lineage specification

To further validate the dose-dependent effects of Gata3 on cell fate, we isolated multiple clones from the Gata3-OE line that displayed heterogeneous levels of ectopic Gata3 expression. From these, we selected clones with high (Gata3-H) and low (Gata3-L) Gata3 expression levels, defined relative to the parental Gata3-OE line, for detailed analysis. These clones differed by over fivefold in Gata3 transcript levels (Fig. 1F). After 2 days of Dox induction, Gata3-L clones retained a rounder shape but appeared more differentiated than uninduced controls, whereas Gata3-H clones exhibited a flattened morphology (Fig. 1G).

We then analyzed protein expression of lineage markers in Gata3-L and Gata3-H clones to explore the mechanisms underlying these morphological differences and lineage bias. Consistent with our earlier observations, Gata3-L and Gata3-H clones showed distinct marker expression patterns: Gata3-L clones displayed higher expression of Gata6, partial induction of Cdx2, and sustained expression of pluripotency markers (Nanog, Oct4), consistent with their rounder morphology. On the other hand, Gata3-H clones showed the opposite trend, with elevated Cdx2, marginal Gata6, and decreased pluripotency marker expression (Fig. 1H). Immunofluorescence again revealed largely non-overlapping Cdx2 and Gata6 signals, with approximately 92% of Gata3-L cells being Gata6-positive only and 87% of Gata3-H cells being Cdx2-positive (Fig. EV1G,H), confirming that individual clones adopt predominantly PE- or TE-biased fates.

To evaluate long-term lineage commitment upon Gata3 induction, we cultured Gata3-L and Gata3-H clones under continuous Dox treatment for 6 days. Gata3-L clones acquired morphological features consistent with XEN-like cells (Niakan et al, 2013), whereas Gata3-H clones adopted TS-like morphology (Tanaka, 2006; Tanaka et al, 1998), suggesting that sustained Gata3 expression drives lineage commitment in a dose-dependent manner (Fig. EV1I).

### Gata3 dosage determines lineage-specific transcriptional programs

**Figure 2:**
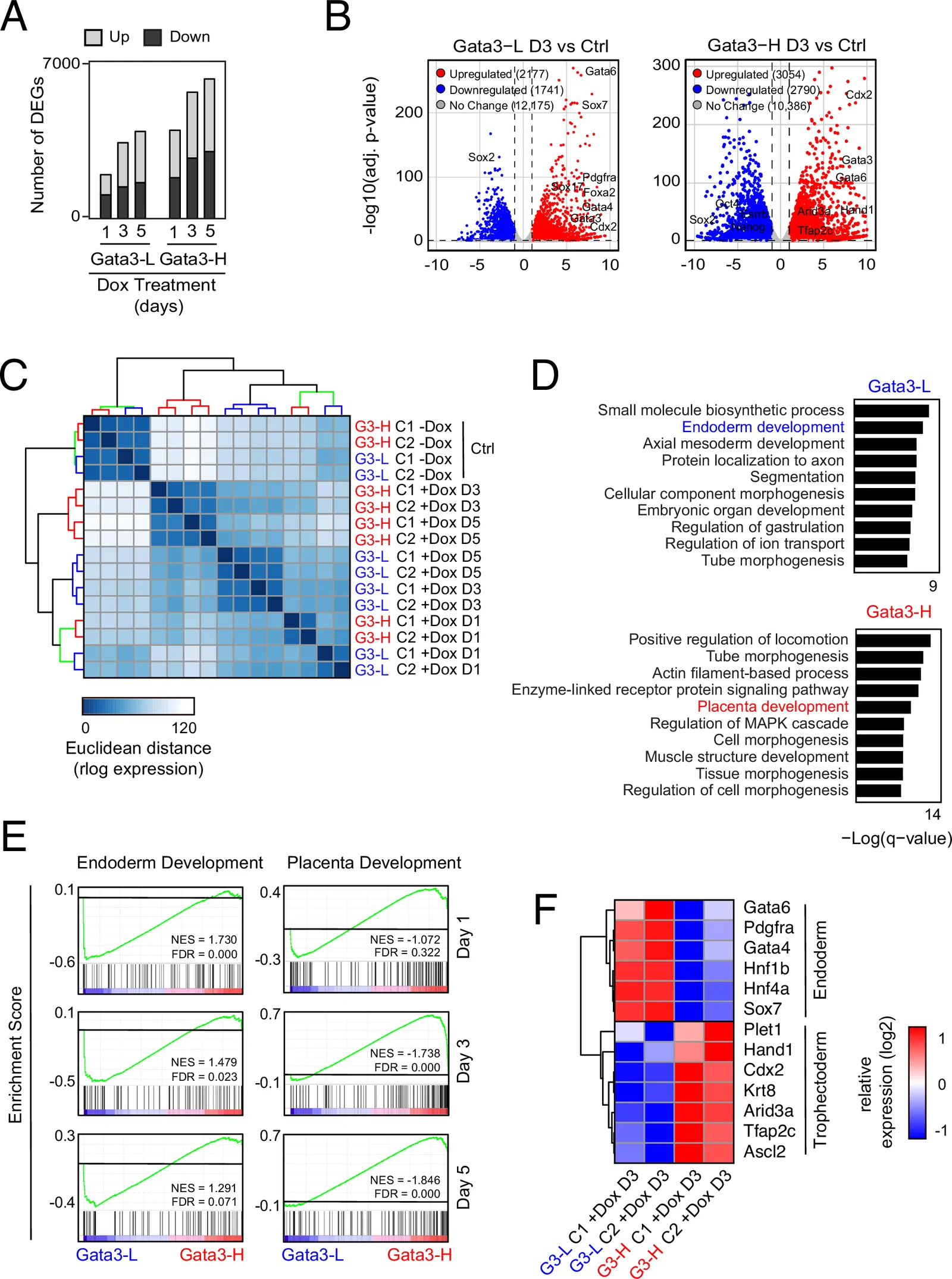
Gata3 dosage shapes transcriptomic programs and lineage-specific gene expression. (**A**) Bar graph showing the number of differentially expressed genes (DEGs) in Gata3-L and Gata3-H clones induced with 0.5 µg/mL Dox for days 1, 3, and 5. DEGs were identified by comparing the transcriptomes of Gata3-induced samples with -Dox control samples. Both upregulated and downregulated genes are represented. (**B**) Volcano plot showing DEGs between Gata3-L or Gata3-H clones and control (−Dox) samples after 3 days of 0.5 µg/mL Dox treatment (*n* = 2 biological replicates per condition; cutoff: |log_2_FC|>1, adjusted *P* value <0.05, Wald-test with Benjamini–Hochberg correction). (**C**) Heatmap showing sample-to-sample distances among the Gata3-L (G3-L) and Gata3-H (G3-H) clones at 1, 3, and 5 days of induction, as well as -Dox control samples. C1 and C2 represent different clones. (**D**) Gene ontology (GO) analysis of upregulated genes in Gata3-L and Gata3-H clones compared to control (−Dox) samples. Day 3 induced samples were used for this analysis. (**E**) Gene set enrichment analysis (GSEA) comparing Gata3-L and Gata3-H clones using endoderm development and embryonic placenta development gene sets obtained from the Molecular Signatures Database (MSigDB). NES and FDR indicate normalized enrichment score and false discovery rate, respectively. (**F**) Heatmap of log_2_-transformed DESeq2-normalized counts of key endoderm and trophectoderm gene expressions in G3-L and G3-H clones after 3 days of induction. Source data are available online for this figure.

**Figure EV2:**
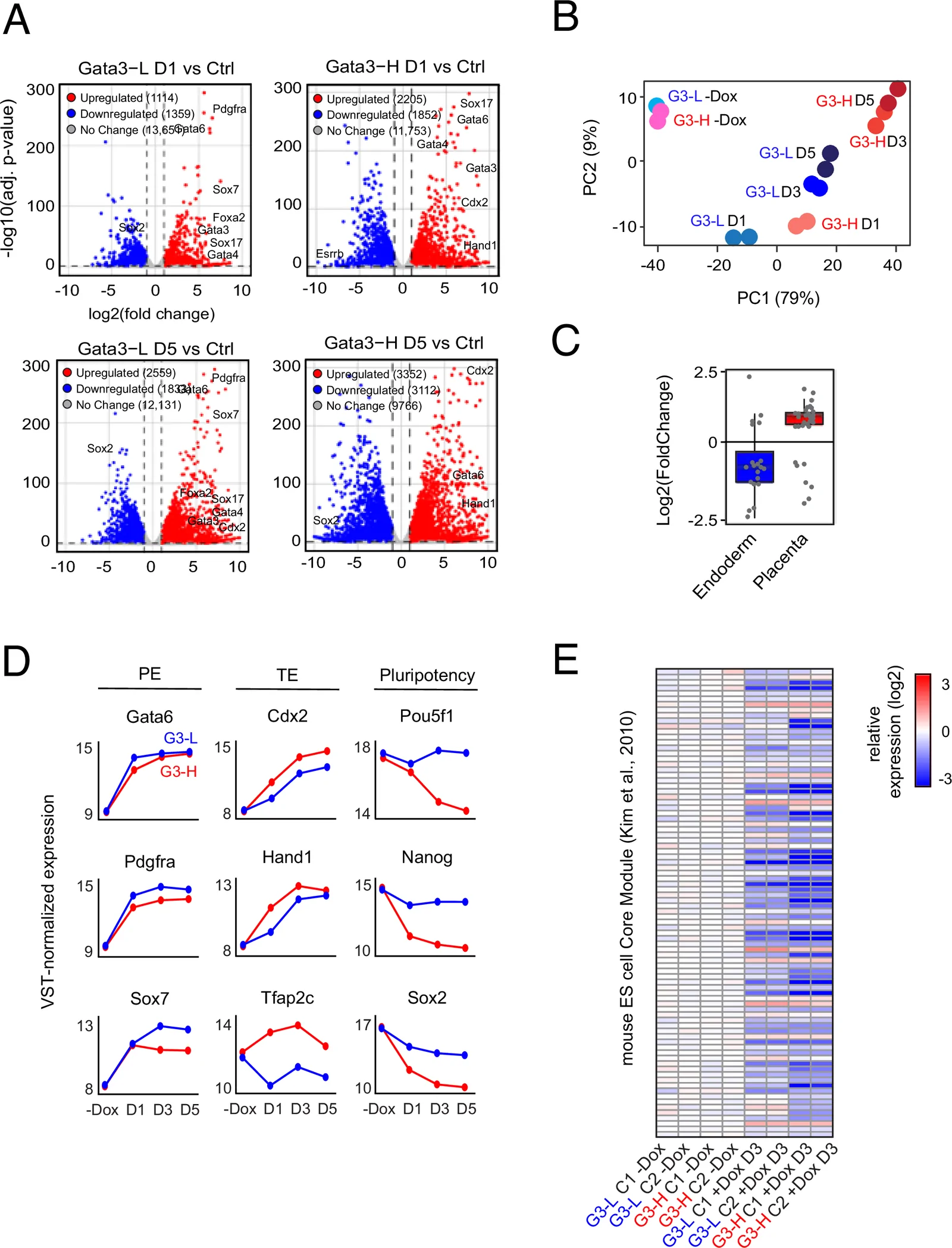
Dose-dependent Gata3 induction reshapes transcriptional programs and fate-associated gene expression. (**A**) Volcano plot showing differentially expressed genes (DEGs) between Gata3-L or Gata3-H clones and control (−Dox) after 1 and 5 days of 0.5 µg/mL Dox treatment (*n* = 2 biological replicates per condition; cutoff: |log_2_FC|>1, adjusted *P* value <0.05, Wald-test with Benjamini–Hochberg correction). (**B**) Principal component analysis (PCA) illustrating the transcriptome profiles of G3-L (blue) and G3-H (red) cells after 1, 3, and 5 days of Dox treatment (0.5 µg/mL). −Dox samples were used as controls. (**C**) Comparison of log_2_(Fold Change) between genes associated with endoderm and placenta, indicated in blue and red, respectively. Negative values represent upregulation in Gata3-L samples, while positive values represent upregulation in Gata3-H samples. The box plot is indicated by the center line, representing the median, the bounds of the box representing the 25th and 75th percentiles, and the whiskers representing the minimum and maximum values (*n* = 2 biological replicates per condition). (**D**) Variance-stabilizing transformed (VST) expression values of key PE, TE, and pluripotency markers in G3-L (blue) and G3-H (red) clones across a time-course of Dox treatment (0.5 µg/mL) at -Dox, 1, 3, and 5 days. (**E**) Expression patterns of the ES cell core module (Kim et al, 2010) after 3 days of induction for G3-L and G3-H samples compared to the control (−Dox). C1 and C2 represent different clones.

We next performed time-course RNA-sequencing (RNA-seq) on Gata3-L and Gata3-H clones to investigate how Gata3 dosage influences the global gene expression during lineage specification. Samples were collected on 1, 3, and 5 days following Dox treatment and we found that the number of differentially expressed genes (DEGs) increased over time in both clones (Fig. 2A). In Gata3-L clones, volcano plots showed a progressive upregulation of key PE markers, including Gata6, Sox7, and Pdgfra, along with decreased expression of pluripotency marker Sox2 (Figs. 2B and EV2A). Interestingly, in Gata3-H clones, some PE markers were initially upregulated at day 1, but over time, the expression profile shifted toward a TE-like identity, with significant upregulation of TE markers such as Cdx2, Hand1, Arid3a, and Tfap2c. Pluripotency markers (Oct4, Sox2, and Nanog) were rapidly declined in Gata3-H clones, indicating a shift away from the self-renewal state (Figs. 2B and EV2A).

Sampe-to-sample correlation heatmap and principal component analysis (PCA) demonstrated that transcriptomes of Gata3-L and Gata3-H clones were initially similar on day 1 but diverged progressively with induction time, suggesting distinct transcriptional responses driven by ectopic Gata3 expression levels (Figs. 2C and EV2B). Gene ontology (GO) analysis of upregulated genes revealed enrichment of the term “Endoderm Development” in Gata3-L clones, while Gata3-H clones were enriched for “Placenta development”, consistent with PE and TE fates, respectively (Fig. 2D). Gene set enrichment analysis (GSEA) further supported these findings, showing significant enrichment of “Endoderm development” gene set in Gata3-L clones and “Placenta Development” related gene set in Gata3-H clones (Fig. 2E). Notably, the enrichment of endoderm-related terms in Gata3-L clones gradually declined over time, while placenta-related terms in Gata3-H clones progressively increased.

Building on the pathway-level analysis, we next assessed the behavior of PE- and TE-associated gene sets. Box plot analysis of log2 fold changes (log2FC) revealed a clear distinction: genes higher in Gata3-L cells (negative log_2_FC) were predominantly endoderm-related genes, whereas genes higher in Gata3-H cells (positive log_2_FC) were largely placenta-associated (Fig. EV2C; Table EV1). At the individual gene level, each PE and TE marker displayed divergent expression profiles (Fig. 2F). To investigate whether Gata3 represses the pluripotency transcriptional network in a dosage-dependent manner, we examined the expression patterns of genes constituting the ES cell core self-renewal module (Kim et al, 2010). Expression of these genes decreased upon Gata3 induction, with a more pronounced reduction in Gata3-H clones compared to Gata3-L clones (Fig. EV2D,E). Together, these findings highlight how Gata3 dosage shapes distinct transcriptional landscapes and drives lineage-specific fate decisions.

### Gata3 levels affect binding intensity at cis-regulatory elements associated with PE or TE genes

As different levels of Gata3 induce distinct gene expression programs, we sought to investigate how Gata3 dosage shapes its chromosomal binding landscapes to drive distinct lineage specification. Using the SBFB system (Kim et al, 2024; Lee et al, 2024), we performed biotin-mediated chromatin immunoprecipitation followed by sequencing (bioChIP-seq) upon 2 days of Dox induction to map Gata3 genomic targets in Gata3-L and Gata3-H clones.

**Figure EV3:**
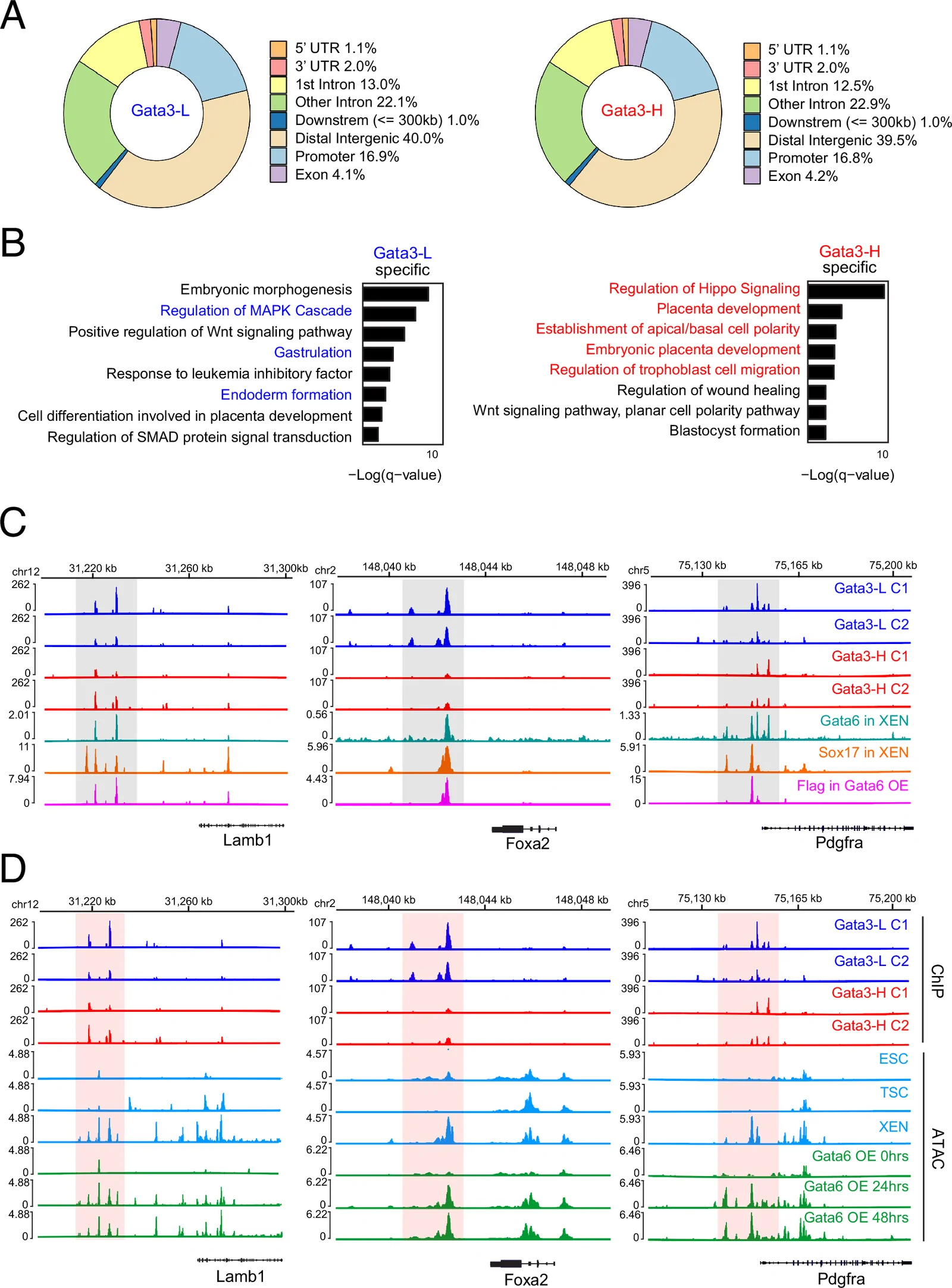
Gata3 binding profiles reveal dosage-dependent functional programs and convergence with XEN chromatin at key PE loci. (**A**) Pie charts showing the genomic distribution of Gata3 binding sites in Gata3-L and Gata3-H clones. (**B**) Enriched GO terms were identified for genes associated with Gata3-L-specific (left) and Gata3-H-specific (right) Gata3 binding regions, as defined by differential binding analysis. The top biological processes are shown, highlighting functional differences associated with low versus high Gata3 occupancy. (**C**) Genome track view showing Gata3 binding loci in Gata3-L and Gata3-H clones, along with Sox17 and Gata6 binding loci in XEN cells and Gata6-OE ES cells near key PE genes. Gray boxes indicate the highlighted regions of interest. (**D**) Genome track view showing Gata3 binding loci in Gata3-L and Gata3-H clones, together with ATAC-seq profiles of ES and TS cells, XEN cells, and Gata6-OE cells after 0, 24, and 48 h post-induction near key PE genes. Red boxes highlight regions of interest.

**Figure 3:**
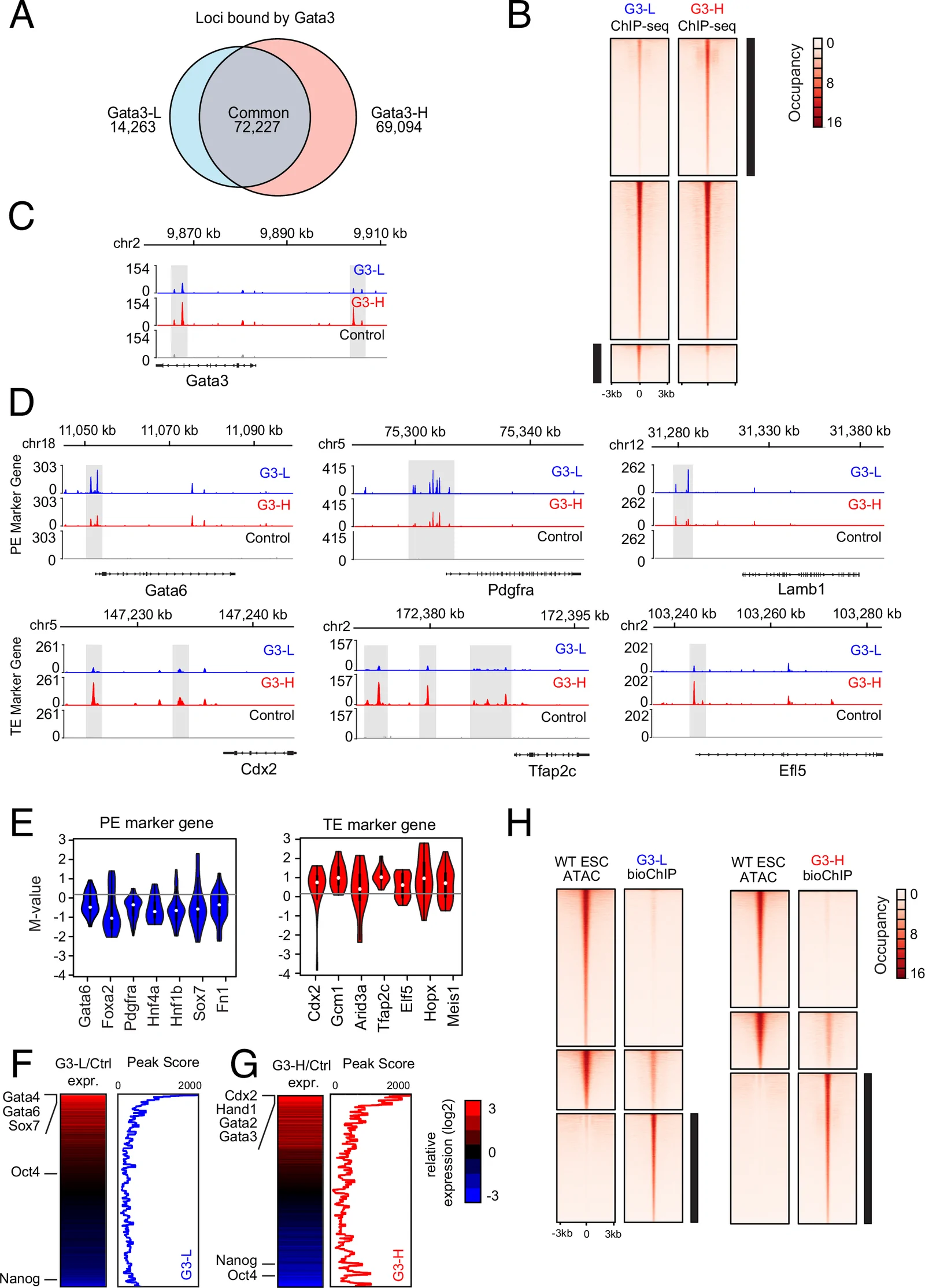
Gata3-L and Gata3-H clones share binding regions with distinct binding intensities near key PE and TE genes. (**A**) Venn diagram illustrating the genes targeted by Gata3 in Gata3-L and Gata3-H samples. (**B**) Heatmap showing the Gata3 bioChIP-seq signals across all binding loci in G3-L and G3-H samples. Peaks were clustered into three groups based on differential binding analysis using DiffBind: Gata3-L-specific (black box, left), common, and Gata-H-specific (black box, right) regions (cutoff: |log_2_FC| >2, adjusted *P* value <0.05, Wald-test with Benjamini–Hochberg correction). (**C**, **D**) Genome track views of Gata3 bioChIP-seq binding profiles in G3-L and G3-H clones at (**C**) the Gata3 locus and (**D**) selected PE and TE marker genes. Significant peak regions are indicated by gray boxes. (**E**) Violin plots of *M*-values for peaks associated with PE and TE marker genes. M-values were calculated by comparing bioChIP-seq profiles of G3-L and G3-H clones using the BETA tool. Negative M-values represent preferential Gata3 binding in G3-L clones, while positive M-values represent stronger binding in G3-H clones. (**F**, **G**) Heatmaps presenting the log_2_-normalized gene expression comparing (F, left) G3-L vs. control (−Dox) and (G, left) G3-H vs. control (−Dox). Genes are ordered by their relative expression levels in each comparison. Corresponding Gata3 occupancy signals for the ordered genes are displayed as moving-window averages (window size  = 100, bin size = 1) (**F**, **G**, right). (**H**) Heatmaps showing Gata3 bioChIP-seq signals in G3-L and G3-H samples alongside ATAC-seq signals from control (−Dox) ES cells. Black bars indicate regions that were closed in control ES cells but became accessible with Gata3 expression. Source data are available online for this figure.

Both clones exhibited widespread Gata3 occupancy at distal regulatory elements with similar chromatin architectures, suggesting that ectopic Gata3 acts like a typical cell type-specific TF regardless of the level of induction (Fig. EV3A). As expected, based on expression levels, Gata3-H clones showed substantially more Gata3-bound loci (141,321 loci) than Gata3-L clones (86,490 loci) (Fig. 3A). Although many target loci were shared, each clone also exhibited a distinct set of unique binding sites (Fig. 3B). Notably, Gata3-L-specific target regions were predominantly enriched for endoderm-related terms, including MAPK cascade, endoderm development, gastrulation, along with some placenta-related terms (Fig. EV3B). In contrast, Gata3-H-specific regions were exclusively enriched for placenta-related terms, such as Hippo signaling regulation, placenta development, and apical/basal cell polarity establishment (Fig. EV3B).

Cell fate master regulators are known to form a self-regulatory circuit (Boyer et al, 2005; Pan and Thomson, 2007). Because Gata3 exhibits dosage-dependent bipotent activity, we assessed whether this applies to both Gata3-L and Gata3-H clones. As expected, Gata3 bound to its own regulatory regions in both clones, consistent with its role as a master regulator, with stronger occupancy signals observed in Gata3-H clones (Fig. 3C). Importantly, Gata3 also occupied regulatory regions near both PE- and TE-associated marker genes in both clones, but with distinct binding strengths; despite lower overall expression, Gata3-L clones showed stronger occupancy at cis-regulatory elements of PE markers, such as Gata6, Pdgfra, and Lamb1 (Fig. 3D). In contrast, Gata3-H clones exhibited stronger binding at TE-associated loci including Cdx2, Tfap2c, and Elf5.

We quantitatively assessed these binding differences using MAnorm, a tool for assessing differential ChIP-seq peak intensities (Shao et al, 2012). Positive *M* values indicated stronger binding in Gata3-H clones, while negative values represented stronger Gata3 binding in Gata3-L clones. As shown in Fig. 3E, many PE marker genes displayed negative *M* values, reflecting stronger Gata3 occupancy in Gata3-L clones, whereas TE marker genes generally showed positive *M* values, suggesting preferential Gata3 binding in Gata3-H clones.

To investigate whether Gata3 binding correlates with transcriptional activation, we integrated the bioChIP-seq data with gene expression profiles from RNA-seq datasets. We observed a strong global correlation between Gata3 binding strength and gene expression across both clones, supporting Gata3’s role as a transcriptional activator. While both clones exhibited Gata3 occupancy and upregulation of genes associated with PE and TE lineages, the most strongly enriched targets differed: In Gata3-L clones, PE-associated genes including Gata4, Gata6, and Sox7 were most strongly upregulated (Fig. 3F), while in Gata3-H clones, TE-associated genes such as Cdx2, Hand1, and Gata2 were preferentially enriched (Fig. 3G). These results indicate that Gata3 functions as a dose-sensitive transcriptional activator, targeting distinct lineage-specific programs.

Building on the discovery of stronger Gata3 binding at PE markers in Gata3-L cells, we compared Gata3-bound loci to publicly available CUT&RUN-seq and ChIP-seq datasets from XEN cells with Gata6 and Sox17 occupancy (Pham et al, 2023; Data ref: Pham et al, 2023) and Gata6-OE ES cells representing XEN-like expression (Wamaitha et al, 2015; Data ref: Wamaitha et al, 2015). Notably, Gata3 binding near PE genes in both clones closely resembled the binding profiles of Gata6 and Sox17 in XEN or XEN-like cells, with stronger occupancy signals in Gata3-L clones (Fig. EV3C). These results further support the idea that lower levels of Gata3 activates a PE-like transcriptional program analogous to that driven by canonical PE regulators, such as Gata6 and Sox17.

### Ectopic Gata3 targets closed chromatin regions in mouse ES cells

Since many master TFs involved in cell fate conversion possess pioneer factor activity, enabling them to bind closed chromatin and initiate chromatin remodeling (Iwafuchi-Doi and Zaret, 2016; Morris, 2016), we next investigated whether Gata3 exhibits similar properties. To test this, we analyzed genome-wide Gata3 occupancy in both Gata3-L and Gata3-H clones and compared these profiles to chromatin accessibility in wild-type ES cells using ATAC-seq data. We found that Gata3 binds both open and closed chromatin regions, indicating that it has a pioneer factor-like capacity to access and potentially activate previously silent, lineage-specific regulatory elements (Fig. 3H).

To further evaluate this, we examined Gata3 binding at specific PE-related genes, where its strong occupancy revealed a previously unrecognized role for PE-associated chromatin remodeling. Using ATAC-seq datasets from mouse ES cells (Lee et al, 2024; Data ref: Lee et al, 2024), trophoblast stem (TS) cells (Hada et al, 2022; Data ref: Hada et al, 2022), XEN cells (Pham et al, 2023; Data ref: Pham et al, 2023), and Gata6-OE ES cells (Thompson et al, 2022; Data ref: Thompson et al, 2022), we compared chromatin accessibility at Gata3-bound loci. Notably, Gata3 occupied regulatory regions near PE-associated genes that display low chromatin accessibility in ES and TS cells but become accessible in XEN and Gata6-OE ES cells. These patterns are consistent with a model in which PE specification involves chromatin opening at these loci, potentially facilitated by Gata3 binding in Gata3-L cells, where occupancy at PE loci is strongest (Fig. EV3D). Together, these findings highlight that Gata3 is not only a fate determinant but also exerts pioneer factor-like activity in reshaping chromatin landscapes during PE specification.

### Generation of blastocyst-like organoids using ES cells with different levels of Gata3 induction

Our findings thus far suggest that Gata3-L clones resemble PE-like cells, while Gata3-H clones exhibit TE-like characteristics. To assess the functional significance of these differences, we employed an in vitro blastoid model, an engineered blastocyst-like structure (Sozen et al, 2019), using ES cells with varying levels of Gata3 induction.

**Figure EV4:**
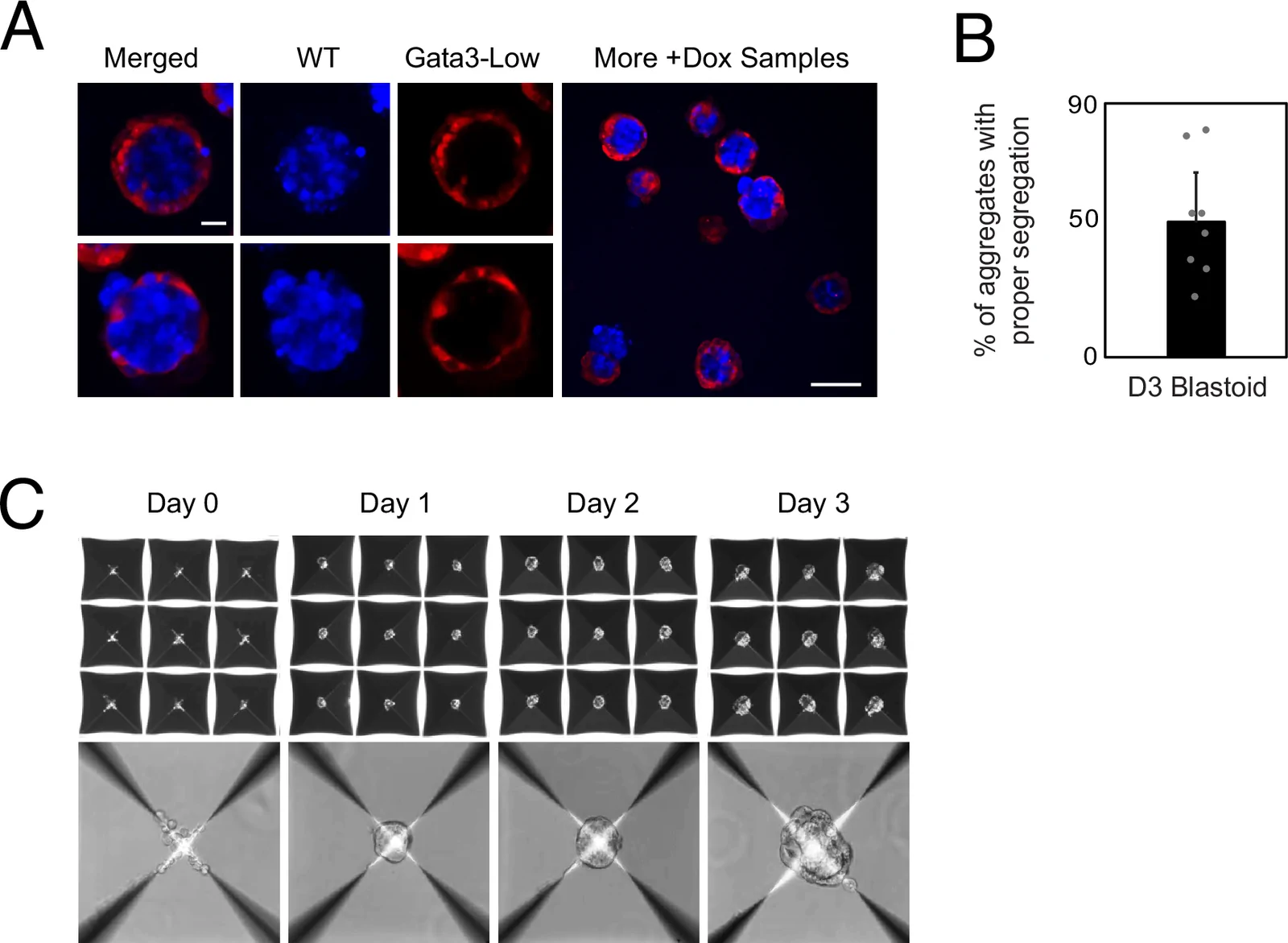
Gata3 induction promotes structural organization during blastoid formation. (**A**) Confocal images of wild-type ES cells and Gata3-L cell aggregates after 3 days of aggregation. A larger field of the +Dox samples is shown on the right. Scale bars: 20 µm (left), 100 µm (right). (**B**) Percentage of aggregates with proper segregation after 3 days of induction. Dots represent percentages from individual random fields of view. The bar indicates the mean, and the error bar represents ±SEM. *n* = 8 across two biological replicates. (**C**) Brightfield images of blastoids in an Aggrewell 400 plate throughout the culture protocol. Top: nine wells showing homogeneous blastoids. Bottom: representative image of an individual blastoid. Source data are available online for this figure.

We first tested whether moderate Gata3 induction in Gata3-L cells, associated with PE-like identity, was sufficient to drive functional segregation from wild-type ES cells. Gata3-L cells were pre-induced with Dox for 24 h prior to aggregation, then mixed with wild-type ES cells in AggreWell plates. Following an additional 48 h of Dox treatment post-aggregation, clear spatial organization emerged, consistent with previous findings where PE-like Gata4-induced ES cells segregated around wild-type ES cells (Fig. EV4A) (Amadei et al, 2021). Similarly, Gata3-L cells localized to the periphery of the aggregates in approximately 48% of blastoids by day 3 (Fig. EV4B), demonstrating functional segregation from wild-type ES cells.

**Figure 4:**
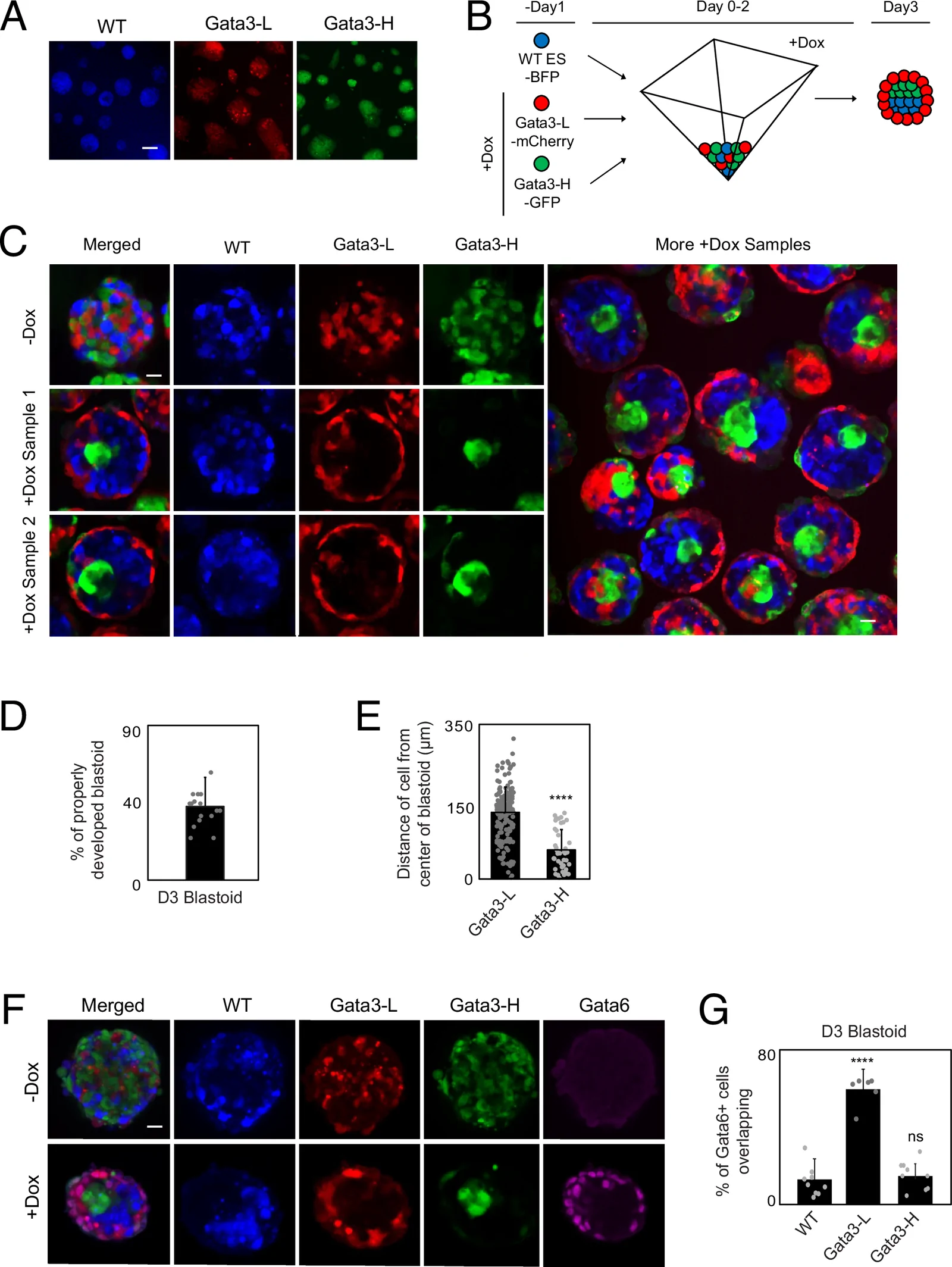
Self-assembly and spatial segregation in blastoids with differential Gata3 expression. (**A**) Fluorescent images of WT (blue), Gata3-L (red), and Gata3-H (green) ES cells after induction. Scale bar: 50 µm. (**B**) Schematic diagram of blastoid aggregation in an Aggrewell plate using fluorescently labeled wild-type ES-BFP cells (blue), Gata3-L-mCherry cells (red), and Gata3-H-GFP cells (green) to assess self-assembly and spatial localization. (**C**) Confocal images of blastoids after 3 days of aggregation. The −Dox sample served as a control, while Dox-induced (+Dox) samples showed proper segregation. A larger field of the +Dox samples is shown on the right. Scale bar: 20 µm. (**D**) Percentage of properly developed blastoids after 3 days of induction. Dots represent percentages from individual random fields of view. The bar indicates the mean, and the error bars represent ±SEM (*n* = 17) across four biological replicates. (**E**) Distance of Gata3-L and Gata3-H cells from the center of the blastoid. Dots represent distances of individual cells from the center of blastoids from random fields of view. Bars indicate the mean, and the error bars represent ±SEM. *P* values were calculated using Student’s *t*-test (*P* = 2.30e-15, *n* = 176 (Gata3-L), *n* = 42 (Gata3-H) across four biological replicates). (**F**) Immunofluorescence analysis of day 3 blastoids stained for Gata6, with (+Dox) and without (−Dox) induction. Scale bar: 20 µm. (**G**) Percentage of cells with Gata6 signals overlapping with wild-type (control), Gata3-L, and Gata3-H cells in day 3 blastoids. Dots represent values from individual random fields of view. Bars indicate the mean, and the error bars represent ±SEM. *P* values were calculated using Student’s *t*-test (WT vs Gata3-L: *P* = 2.23e-8; WT vs Gata3-H: *P* = 0.19; WT and Gata3-H: *n* = 9, Gata3-L: *n* = 8 across four biological replicates). Source data are available online for this figure.

Building on these results and given that mixtures of wild-type ES cells with iCdx2 (TE-like) and iGata6 (PE-like) ES cells can form blastocyst-like structures in vitro (Lau et al, 2023; Tarazi et al, 2022), we tested whether combining wild-type, Gata3-L, and Gata3-H ES cells could self-organize into compartmentalized blastoids based solely on Gata3 expression levels. To track the cell source, distinct fluorescent reporters were introduced into each cell type to track the localization (Fig. 4A). After 24 h of Dox pre-treatment, cells were mixed at defined ratios (6000 wild-type ES, 6000 Gata3-L, and 20,000 Gata3-H cells) in AggreWell plates to generate blastocyst-like organoids and cultured for 3 days (Fig. 4B). By day 3 of continuous Dox induction, the blastoids displayed emerging structural organization and signs of layer formation (Fig. EV4C).

Remarkably, while uninduced control organoids displayed random cell distribution, Dox-induced blastoids consistently formed well-organized architectures with three distinct compartments: Gata3-L-mCherry-positive cells forming an outer layer surrounding clusters of Gata3-H-GFP-positive cells and wild-type-BFP-positive cells (Fig. 4C). This spatial organization was observed in approximately 40% of blastoids, closely resembling the compartmentalized patterns of previous blastoid models (Lodewijk et al, 2025; Tarazi et al, 2022) (Fig. 4D). To quantify this pattern, we measured the radial distance of Gata3-L and Gata3-H cells from the blastoid center. Notably, Gata3-L cells located significantly further from the center than Gata3-H cells, indicating that lower Gata3 expression promotes peripheral positioning (Fig. 4E).

To further validate the role of Gata3 in PE identity, we performed immunofluorescence staining for Gata6, a key PE marker, in day 3 blastoids. Gata6 expression strongly overlapped with Gata3-L-mCherry-positive cells at the periphery (Fig. 4F), with ~60% of Gata6-positive cells co-localizing with Gata3-L cells, confirming their PE-like identity (Fig. 4G). Together these results demonstrated that Gata3 dosage alone is sufficient to drive spatial segregation within blastoids, directing ES cell fate toward PE or TE lineages. These findings highlight Gata3’s critical role in recapitulating lineage compartmentalization during early embryogenesis.

### Single-cell transcriptomic analysis reveals dose-dependent lineage bias in blastoids

**Figure 5:**
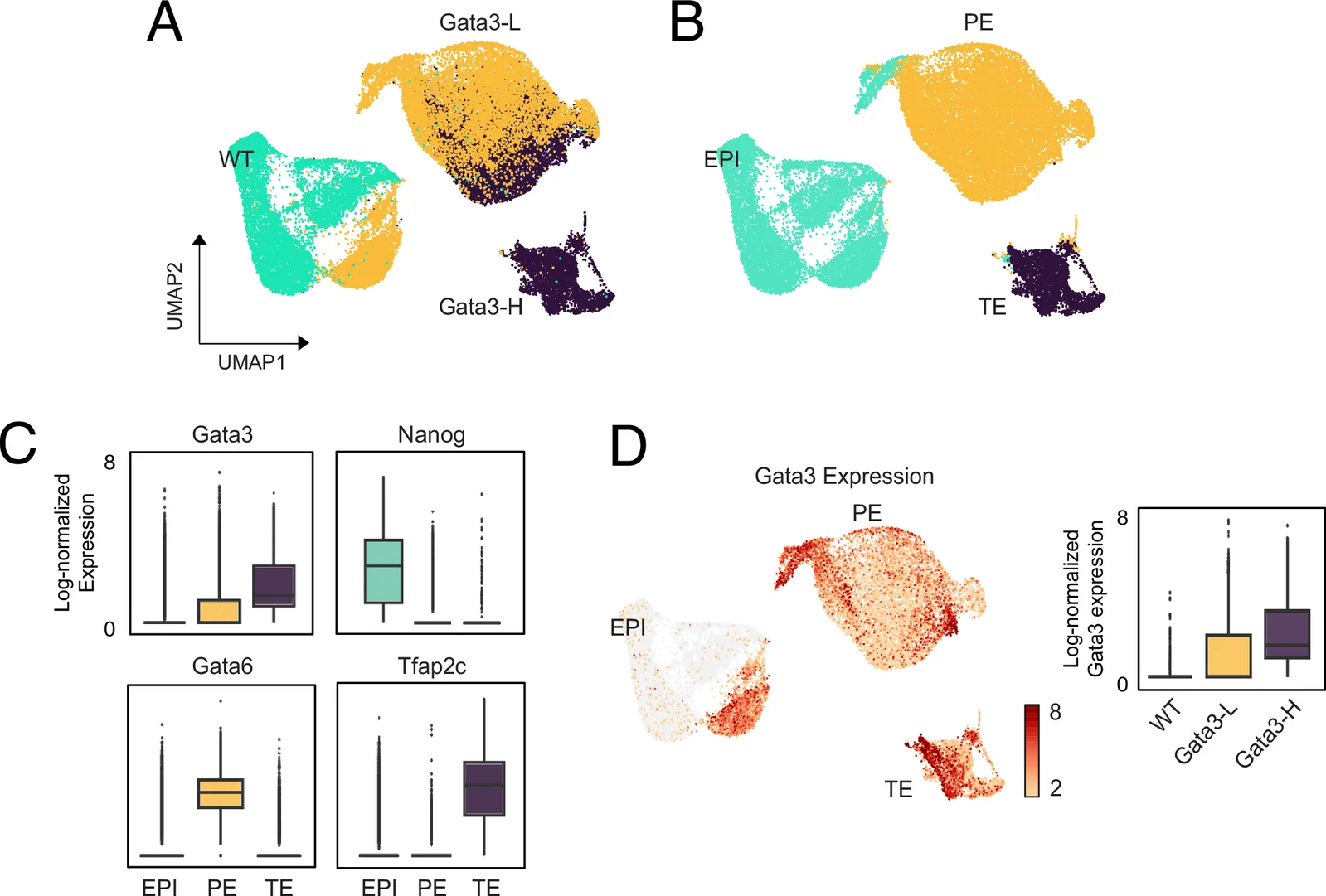
Gata3 dosage directs cell fate specification in blastoids. (**A**) UMAP showing the distribution of WT, Gata3-L, and Gata3-H cells across clusters, with predominant enrichment in their expected lineages and some overlap, including Gata3-L cells in the EPI cluster and Gata3-H cells in the PE cluster. (**B**) UMAP of unsupervised k-means clustering of day 3 blastoids after FACS sorting of WT, Gata3-L, and Gata3-H cells reveals three major clusters. Data were analyzed using the Seurat v5.3.1 package. (**C**) Cluster assignment to EPI-, PE-, and TE-like lineages based on canonical lineage marker expression. In all box plots, the center line represents the median, the bounds of the box represent the 25th and 75th percentiles, and the dotted whiskers represent the minimum and maximum values. *n* represents the number of individual cells analyzed from a single scRNA-seq run (EPI: *n* = 17278, PE: *n* = 12226, TE: *n* = 3902). (**D**) Gata3 expression across clusters demonstrates the highest expression in TE-like cells and lower expression in PE-like cells. The box plot of Gata3 expression levels across cell distribution is indicated by the center line, representing the median, the bounds of the box representing the 25th and 75th percentiles, and the dotted whiskers representing the minimum and maximum values. *n* represents the number of individual cells analyzed from a single scRNA-seq run (WT: *n* = 7948, Gata3-L: *n* = 13204, Gata3-H: *n* = 12254). Source data are available online for this figure.

**Figure EV5:**
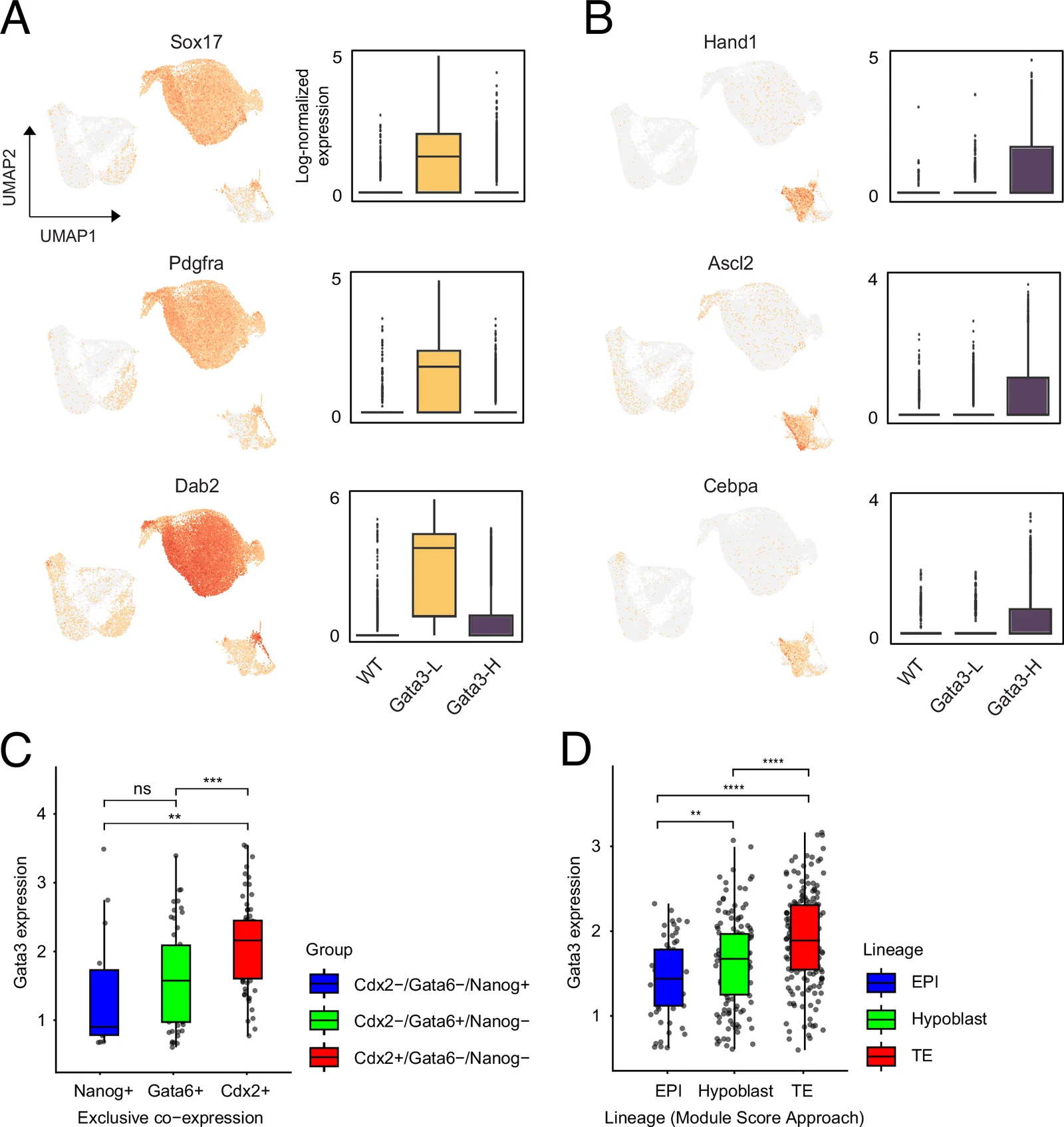
Gata3-L and Gata-H clones acquire mature PE- and TE-like transcriptional programs in blastoids, recapitulating Gata3 expression patterns observed in vivo in the blastocyst. (**A**,** B**) Expression of mature lineage-associated markers shown as UMAP feature plots and corresponding bar graph. In all box plots, the center line represents the median, the bounds of the box represent the 25th and 75th percentiles, and the dotted whiskers represent the minimum and maximum values. *n* represents the number of individual cells analyzed from a single scRNA-seq run: (**A**) PE markers (Sox17, Pdgfra, and Dab2) in Gata3-L cells, and (**B**) TE markers (Hand1, Ascl2, and Cebpa) in Gata3-H cells (WT: *n* = 7948, Gata3-L: *n* = 13204, Gata3-H: *n* = 12254). (**C**) Mouse E4.5 blastocysts scRNA-seq data, with lineage defined using exclusive expression of lineage markers. The box plot is indicated by the center line, representing the median, the bounds of the box representing the 25th and 75th percentiles, and the whiskers representing the minimum and maximum values. Data were presented as mean ± SEM. Statistical significance was determined using Student’s *t*-test. The adjusted *P* values for the comparison between Nanog+ lineage (*n* = 15), Gata6+ lineage (*n* = 50) is *P* = 0.36 (ns). Significant differences were observed for Nanog+ vs. Cdx2+ (*n* = 71; *P* = 0.0086) and Gata6+ vs. Cdx2+ (*P* = 0.00035). (**D**) Human blastocyst analyzed by scRNA-seq, with lineage defined using a module score approach. The box plot is indicated by the center line, representing the median, the bounds of the box representing the 25th and 75th percentiles, and the whiskers representing the minimum and maximum values. Data were presented as mean ± SEM. Statistical significance was determined using Student’s *t*-test. The adjusted *P* values are as follows: EPI (*n* = 61) vs. Hypoblast (*n* = 61), *P* = 0.0096; EPI vs. TE (*n* = 140), *P* = 2.5e-9; Hypoblast vs. TE, *P* = 1.7e-5.

To further define how varying levels of Gata3 influence lineage commitment in blastoids, we performed single-cell RNA-sequencing (scRNA-seq) on day 3 blastoids generated from FACS-sorted WT, Gata3-L, and Gata3-H cells, each labeled with distinct fluorescent markers. UMAP visualization of the combined populations revealed segregation of cells according to their originating Gata3 dosage (Fig. 5A). Unsupervised k-means clustering identified three major transcriptional states corresponding to EPI, PE-, and TE-like lineages (Fig. 5B), as defined by canonical marker expression (Fig. 5C). WT, Gata3-L, and Gata3-H cells were predominantly enriched in EPI, PE-like, and TE-like clusters, respectively. Within clusters, Gata3 expression levels stratified according to dosage, with lower expression associated with PE-like states and higher expression associated with TE-like states (Fig. 5C,D). Examination of established PE (Sox17, Pdgfra, Dab2) and TE (Hand1, Ascl2, and Cebpa) markers further confirmed activation of lineage-associated programs, indicative of a more matured state, with enrichment consistent with Gata3 dosage (Fig. EV5A,B). Collectively, these data demonstrate that Gata3 dosage systematically biases lineage specification in blastoid models.

## Discussion

TFs are known to drive cell fate transitions, but how their expression levels modulate lineage outcomes remains poorly defined. In most cases, TFs are restricted to directing a single lineage, raising the question of how dosage-sensitive regulators may influence multiple fate choices (Niwa et al, 2005; Wamaitha et al, 2015). In this study, we demonstrated that Gata3, a canonical regulator of TE identity, functions as a dose-sensitive determinant of early lineage specification in mouse ES cells. Low Gata3 expression preferentially induced PE-like differentiation, whereas high expression promoted TE lineage specification, each associated with distinct gene expression programs and genomic binding landscapes. Incorporation of these cells to form 3D blastoids revealed spatial segregation aligned with Gata3 dosage, showing that Gata3 expression alone is sufficient to direct compartmentalized lineage organization in vitro. These findings establish TF dosage as a critical parameter in developmental fate decisions.

Our results also complement and extend prior in vivo observations. In mice, Gata3 is first induced at the 4-cell stage and remains consistently expressed throughout pre-implantation development, with selective enrichment in the TE lineage at the blastocyst stage (Home et al, 2009). While ectopic expression of Gata3 activates trophoblast gene expression in mouse ES cells, and its downregulation impairs blastocyst maturation (Home et al, 2009; Nishiyama et al, 2009; Ralston et al, 2010), its potential role in PE specification in vivo has not been investigated. Intriguingly, analysis of public single-cell RNA-seq (scRNA-seq) datasets from E4.5 mouse blastocysts revealed detectable Gata3 expression within PE-like clusters alongside canonical PE markers (Chen et al, 2016; Deng et al, 2014; Posfai et al, 2017), suggesting a possible contribution of Gata3 to PE lineage specification in vivo (Fig. EV5C) (Chen et al, 2024; Data ref: Chen et al, 2024). Similar patterns were observed in human blastocysts at 6–8 days post-fertilization (Fig. EV5D) (Zhou et al, 2019; Data ref: Zhou et al, 2019). Further in vivo studies via knockout approaches, or ex vivo studies, such as injecting Gata3-L or Gata3-H cells into early-stage embryos, will help define their lineage potential in a more physiologically relevant developmental context.

Our findings further raise the possibility that dosage sensitivity may extend to Gata3’s close paralog, Gata2. Previous studies have noted functional redundancy between Gata2 and Gata3 in trophoblast development (Home et al, 2017; Paul et al, 2017), suggesting they may share overlapping regulatory potential. Whether Gata2 can similarly compensate for Gata3 in PE specification remains to be tested.

Despite the insights gained, technical challenges limited our ability to model long-term outcomes. Extending blastoids' culture to later developmental stages, like egg-cylinder formation, would strengthen evidence for Gata3’s lineage influence. However, while Dox-inducible systems, such as the SBFB platform, enable precise temporal control, limited sustainability of expression constrained our ability to assess post-implantation morphogenesis. Future strategies incorporating CRISPR activation or alternative gene regulation platforms may overcome these barriers.

Altogether, our work highlights TF dosage as a key determinant of stem cell fate. Beyond early development, precise TF expression levels govern lineage specification, cellular plasticity, and functional identity in diverse contexts, including disease. For instance, graded expression of PU.1 in fetal liver progenitors directs B cell versus macrophage differentiation (DeKoter and Singh, 2000), and TBX5 dosage influences 3D genome architecture during cardiomyocyte differentiation, with implications for congenital heart disease (preprint: Grant et al, 2025). The dosage-sensitive behavior of Gata3 described here underscores a broader principle of how gene regulatory networks interpret TF concentrations to enforce or diversify cell fate decisions. Understanding these dynamics will be critical for decoding cell identity regulation and for harnessing TF dosage in regenerative or therapeutic strategies.

## Methods

**Table:** Reagents and tools table

Reagent/resource	Reference or source	Identifier or catalog number
**Experimental models**		
J1 embryonic stem cells (*M. musculus*)	ATCC	SCRC-1010
CJ7 embryonic stem cells (*M. musculus*)	Dr. Stuart Orkin	N/A
E14 embryonic stem cells (*M. musculus*)	ATCC	CRL-1821
Trophoblast stem cells (*M. musculus*)	Dr. Janet Rossant	N/A
**Recombinant DNA**		
pCMV(CAT)T7-SB100	Addgene	#34879
pSBtet-GP	Addgene	#60495
NEB 5-alpha Competent *E.coli*, High Efficiency	NEB	C2987H
**Antibodies**		
Gata6	R&D Systems	AF1700
Gata3	Cell Signaling	5852S
Cdx2	Abcam	Ab76541
Pou5f1	Santa Cruz Biotechnology	sc-5279
Gata4	Santa Cruz Biotechnology	sc-25310
Sox2	Santa Cruz Biotechnology	sc-365823
Nanog	Bethyl Laboratories	A300-397A
Strep-HRP	Cytiva	RPN1231-2ML
Actb	Abgent	AM1829B
HRP-linked anti-rabbit IgG	Cell Signaling	7074S
HRP-linked anti-mouse IgG	Cell Signaling	7076S
HRP-linked anti-goat IgG	Santa Cruz	sc-2354
Alexa Fluor 488-Rabbit	Cell Signaling	4412S
Alexa Fluor 594-Rabbit	Invitrogen	A11037
Alexa Fluor 647-Goat	Invitrogen	A32849
Alexa Fluor 647-Rabbit	Invitrogen	A31573
**Oligonucleotides and other sequence-based reagents**		
RT-qPCR Primers	This Study	Table EV2
**Chemicals, enzymes and other reagents**		
RNeasy Plus Mini Kit	Qiagen	74136
ProtoScript II First Strand cDNA Synthesis Kit	NEB	E6560S
Notl-HF	NEB	R3189S
Nhel-HF	NEB	R3131S
T4 DNA Ligase	NEB	
Lipofectamine Stem Transfection Reagent	Invitrogen	STEM00008
Puromycin	Gibco	A1113803
G418	Gibco	10131027
Doxycycline (Dox)	Fisher BioReagents	BP26535
Gelatin	MilliporeSigma	G9382
Dulbecco’s Modified Eagle’s Medium (DMEM)	Gibco	11965-118
Fetal Bovine Serum (FBS)	MilliporeSigma	12306 C
EmbryoMax Nucleosides	MilliporeSigma	ES-008-D
MEM non-essential amino acids	Gibco	11140050
β-mercaptoethanol	MilliporeSigma	M3148
Penicillin-Streptomycin-Glutamine (PSG)	Gibco	
Recombinant Mouse LIF	Gemini Bio-Products	400–495
0.25% Trypsin-EDTA	Thermo Fisher Scientific	25200056
Cell Counting Kit	Dojindo	CK0413
CCK-8 reagent	Dojindo	CK0401
qScript™ cDNA SuperMix	QuantaBio	95048-500
PowerUp™ SYBR™ Green Master Mix	Applied Biosystems	A46112
Polyvinylidene Fluoride (PVDF) membrane	MilliporeSigma	IPVH00010
Blotting-Grade Blocker; nonfat dry milk	Bio-Rad	1706404
10X Tris/Glycine Buffer	Bio-Rad	1610734
Tween® 20, for molecular biology, viscous liquid	MilliporeSigma	P9416-100ML
Enhanced Chemiluminescence (ECL) Western Blotting Reagent	Cytiva	45002401
Paraformaldehyde, 96%	Fisher Scientific	AC416785000
Phosphate-buffered saline (PBS), 1X without calcium and magnesium, PH 7.4 ± 0.1	Corning	21-040-CV
Bovine serum albumin (BSA)	MilliporeSigma	A7906
Horse serum	Gibco	16050122
Triton X-100	MilliporeSigma	T8787
NEBNext® Poly(A) mRNA Magnetic Isolation Module	NEB	E7490L
NEBNext® Ultra™ II RNA Library Prep Kit for Illumina	NEB	E7645L
NEBNext® Multiplex Oligos for Illumina	NEB	E7780S
Glycine	Fisher Scientific	BP381-1
GlutaMax	Gibco	35050061
Penicillin-Streptomycin	Gibco	15140122
Sodium pyruvate	Gibco	11360070
Anti-adherence rinsing solution	Stem Cell Technologies	07010
24-well AggreWell 400 Plate	Stem Cell Technologies	34415
Fgf4	R&D Systems	7486F4025
Heparin	MilliporeSigma	H3149-10KU
Y-27632	Selleck Chemicals	S1049
Formaldehyde	MilliporeSigma	F8775
TrypLE Express	Gibco	12604013
µ-Slide eight-well plate	Ibidi	80826
GEM-X Flex Sample Preparation v2 Kit	10X Genomics	PN-1000781
**Software**		
Primer3	https://primer3.ut.ee/	
Trim Galore! (v0.6.10)	https://github.com/FelixKrueger/TrimGalore	
STAR (v2.7.3a)	https://github.com/alexdobin/STAR/issues/832	
samtools (v.1.19.2)	https://github.com/samtools/samtools/releases/	
featureCounts (v1.5.0-p1)	https://subread.sourceforge.net/featureCounts.html	
DESeq2 (1.42.1) R package	https://bioconductor.org/packages/release/bioc/html/DESeq2.html	
Salmon (v1.10.3)	https://github.com/COMBINE-lab/salmon/releases	
Bowtie2 (v2.3.5.1)	https://bowtie-bio.sourceforge.net/bowtie2/index.shtml	
Picard (v2.27.5)	https://broadinstitute.github.io/picard/	
Integrative Genomics Viewer (IGV) (v2.19.6)	https://igv.org	
MACS3 (v3.0.2)	https://github.com/macs3-project/MACS/releases	
HOMER (v5.1)	http://homer.ucsd.edu/homer/motif/HOMER	
deepTools bamCoverage (v3.5.5)	https://deeptools.readthedocs.io/en/develop/content/tools/bamCoverage.html	
DiffBind (v3.12.0)	https://bioconductor.org/packages/release/bioc/html/DiffBind.html	
MAnorm	https://github.com/shao-lab/MAnorm	
Seurat (v5.3.1)	https://cran.r-project.org/web/packages/Seurat/index.html	
GSEA (v4.4.0)	https://www.gsea-msigdb.org/gsea/index.jsp	
Loupe Browser (v9.1)	10X Genomics	
Nikon NIS Elements (v6.10.01)	Nikon	
ImageJ (v1.53)	https://imagej.net/ij/index.html	
**Other**		
Tecan M1000 Plate Reader	Tecan	
StepOnePlus™ Real-Time PCR System	Applied Biosystems	
Zeiss LSM 710 Confocal Microscope	Zeiss	
Illumina NovaSeq 6000	Illumina	
Nikon W1 Spinning Disk Confocal Microscope	Nikon	

### Establishment of stable inducible cell lines for the overexpression of Gata3

The original luciferase open reading frame (ORF) in the pSBtet-GP vector (Addgene) was substituted with a sequence encoding a FLAG tag, a biotinylation site, and multiple cloning sites containing NotI and NheI restriction sites, generating the modified vector pSBFB (Lee et al, 2024). Total RNA was extracted from mouse trophoblast stem (TS) cells, which were derived from blastocyst (TS3.5) and gifted from Dr. Janet Rossant's laboratory, using the RNeasy Plus Mini Kit (Qiagen), and 500 ng of RNA was reverse transcribed into cDNA using the ProtoScript II First Strand cDNA Synthesis Kit (NEB). Gata3 coding sequences were amplified from the cDNA using primers containing NotI and NheI restriction enzyme sites. The pSBFB vector and amplified DNA were digested with Notl and Nhel (NEB), purified, and ligated using T4 DNA Ligase (NEB). Cloned vectors were verified with Sanger sequencing. These vectors were then transfected into BirA-mouse ES cells using Lipofectamine Stem Transfection Reagent (Invitrogen), along with pCMV(CAT)T7-SB100 (Addgene), a transposase vector that facilitates genomic integration in BirA-expressing mouse ES cells. Transduced cells were selected using 1 µg/mL of puromycin (Gibco), and 250 µg/mL of G418 (Gibco) starting 24 h post-transfection to obtain stable cell lines. To induce the expression of Gata3, cells were treated with 0.5 µg/mL of Doxycycline (Dox) (Fisher BioReagents) for 48 h, and protein expression of Gata3 was confirmed with Western blot using Streptavidin-HRP.

### Generation and selection of Gata3 inducible clones

Following the generation of stable Gata3-overexpressing cell lines, a pooled population was maintained under pluripotent conditions. Individual clones were isolated from this pool and expanded in the absence of Dox. To access inducible expression, each clone was treated with 0.5 µg/mL Dox for 48 h, and Gata3 expression levels were quantified by qPCR and Western blot (Streptavidin-HRP). Based on the magnitude of induced Gata3 expression relative to the pooled Gata3-overexpressing cell line, clones were classified as Gata3-L (low induction) or Gata3-H (high induction). Uninduced cells from each clone were maintained in parallel and cryopreserved.

### Cell culture

Mouse ES cells, including the J1, CJ7, and E14 lines were cultured on 0.1% gelatin-coated plates in ES cell culture media, containing Dulbecco’s Modified Eagle’s Medium (DMEM, Gibco), supplemented with 18% fetal bovine serum (FBS, MilliporeSigma), EmbryoMax Nucleosides (MilliporeSigma), MEM non-essential amino acids (Gibco), 0.1 mM β-mercaptoethanol (MilliporeSigma), 50 U/mL Penicillin-streptomycin-glutamine (PSG, Gibco), and 1000 U/mL recombinant mouse LIF (Gemini Bio-Products, 400–495). Cells were maintained in a 37 °C incubator with 5% CO_2_, passaging every 2–3 days by dissociating into single cells with 0.25% trypsin-EDTA (Thermo Fisher Scientific).

### Cell growth assay

Cells were plated in 96-well plates coated with 0.1% gelatin and maintained in mouse ES cell culture medium. Cells were seeded at a density of 5 × 10⁵ cells/mL, and 0.5 µg/mL of Dox was added 24 h post-seeding to induce Gata3 expression. Cell proliferation was assessed using the Cell Counting Kit (Dojindo) at four time points: the day of Dox induction (day 1) and subsequent 24-h intervals until day 4 post-induction. At each time point, the culture medium was replaced with mouse ES cell media containing the CCK-8 reagent (Dojindo) in each well. The plates were incubated at 37 °C for 2 h, and the absorbance at 450 nm was measured using a Tecan M1000 Plate Reader to quantify cell viability and proliferation.

### Colony-forming assay

The self-renewal capacity of Gata3-overexpressing cells was assessed using a colony-forming assay. Gata3 OE cells were seeded into six-well plates coated with 0.1% gelatin at a density of 600 cells per well and cultured in mouse ES cell medium with or without Dox. After 6 days of culture, the resulting colonies were examined under a microscope and classified based on their morphology as either round (indicating self-renewal) or differentiated. The number of colonies in each category was subsequently quantified.

### RNA extraction and RT-qPCR

Total RNA was isolated from Dox-treated and/or untreated Gata3-overexpressing cells using the RNeasy Plus Mini Kit (Qiagen). Reverse transcription to cDNA was conducted using qScript™ cDNA SuperMix (QuantaBio) with 500 ng of total RNA as input. The synthesized cDNA was subsequently diluted 20-fold, and 2 µL of the diluted cDNA was used for each quantitative PCR (qPCR) reaction. qPCR was conducted using the PowerUp™ SYBR™ Green Master Mix (Applied Biosystems) on a StepOnePlus™ Real-Time PCR System (Applied Biosystems). Primers were designed to amplify the junction between two exons using Primer3 (https://primer3.ut.ee/), ensuring specificity (Table EV2). Relative mRNA expression levels were normalized to Gapdh as a loading control, and fold changes were calculated using the 2^−ΔΔCT^ method to express data relative to the control condition. *P* values were determined using three biological replicates with Student’s *t*-test, with significance levels annotated as *, **, and *** for *P* values <0.05, 0.01, and 0.001, respectively.

### Western blot

Dox-treated and untreated cells were lysed using Laemmli Sample Buffer (Bio-Rad) supplemented with 5% β-mercaptoethanol (MilliporeSigma) and heated at 95 °C for 10 min. The lysates were loaded on a sodium dodecyl sulfate (SDS) polyacrylamide gel (7.5–10%), and proteins were transferred onto polyvinylidene fluoride (PVDF) membranes (MilliporeSigma). To block any nonspecific binding, the membranes were incubated in 5% skim milk in TBS-T (Tris-buffered saline with 0.1% Tween-20) for 1 h. Subsequently, the membranes were probed overnight at 4 °C with either Streptavidin-Horseradish Peroxidase conjugates (streptavidin-HRP, Cytiva) or the appropriate primary antibodies (see Reagents and tools table). The membranes were then washed three times with TBS-T for 10 min each wash, followed by incubation with a secondary antibody for 1 h at room temperature. After three additional 10-min washes in TBS-T, protein was visualized using an enhanced chemiluminescence (ECL) Western blotting reagent (Cytiva).

### Immunofluorescence

Gata3-overexpressing cells, with or without Dox treatment, were fixed with 4% paraformaldehyde for 20 min at room temperature and were washed three times with PBS (Corning) containing Ca²⁺ and Mg²⁺ (PBS-CM) with 0.1% bovine serum albumin (BSA, MilliporeSigma). Fixed cells were then permeabilized and blocked in 10% horse serum (Gibco) prepared in PBS-CM/0.1% BSA with 0.3% Triton X-100 (MilliporeSigma) for 45 min at room temperature. The cells were then incubated overnight at 4 °C with primary antibodies in PBS-CM/0.1% BSA with 10% horse serum (see Reagents and tools table), followed by three washes with PBS-CM/0.1% BSA. Secondary antibodies conjugated to Alexa Fluor 488 or Alexa Fluor 594 (Thermo Fisher Scientific) were applied for 2 h at room temperature. After three washes to remove excess antibodies, nuclei were counterstained with 4’,6-diamidino-2-phenylindole (DAPI, MilliporeSigma) for 10 min. The cells were rewashed, and fluorescent images were captured using a Zeiss LSM 710 confocal microscope. Samples within the same experiment were imaged under uniform microscopy settings.

### RNA-seq

Total RNA extraction was performed from different clones of Gata3-overexpressing cells treated with 0.5 µg/mL Dox for 0, 1, 3, and 5 days using the RNeasy Plus Mini Kit (Qiagen). mRNA was isolated from 300 ng of total RNA using the NEBNext® Poly(A) mRNA Magnetic Isolation Module (NEB). The sequencing libraries were prepared using the NEBNext® Ultra™ II RNA Library Prep Kit for Illumina® (NEB) for paired-end sequencing, and indexing was performed using NEBNext® Multiplex Oligos for Illumina® (NEB). The RNA-seq libraries were sequenced on an Illumina NovaSeq 6000 platform. The raw reads were first quality-controlled and trimmed using Trim Galore! (v0.6.10) (https://github.com/FelixKrueger/TrimGalore) with default settings for paired-end data, ensuring a minimum read length of 20 bases. The trimmed reads were then aligned to the GRCm38 reference genome using STAR (v2.7.3a). The resulting BAM files were filtered for mapping quality (MQ ≥10) using samtools (v.1.19.2) to remove low-quality alignments. Gene-level read counts were obtained using featureCounts (v1.5.0-p1) from the aligned reads. Differential gene expression analysis was performed using the DESeq2 (1.42.1) R package, with genes showing a base mean >10, a *P* adjusted value <0.05, and a log_2_(fold change) >1 considered differentially expressed (DEGs). Gene expression levels were also quantified in transcripts per million (TPM) using Salmon (v1.10.3). Gene ontology (GO) and pathway enrichment analyses were conducted on DEGs, as well as gene set enrichment analysis (GSEA) (v4.4.0) (Subramanian et al, 2005) to identify enriched gene sets in the development of the placenta and endoderm.

### bioChIP-seq

bioChIP-seq was performed on different clones of Gata3-overexpressing cells with 0.5 µg/mL Dox for 2 days. Cells were fixed using 1% formaldehyde (Fisher Bioreagents) for 7 min at room temperature, and the reaction was stopped by adding glycine (Fisher Scientific) to achieve a final concentration of 0.125 M. The bioChIP procedure followed the previously described protocol (Kim et al, 2009). Genomic DNA was sonicated into approximately 200 bp fragments using a Bioruptor (Diagenode). The cross-linked DNA fragments were precleared with Protein A Agarose (Thermo Fisher), then immunoprecipitated using Dynabeads MyOne Streptavidin T1 (Invitrogen). Sequencing libraries were prepared using the NEBNext® Ultra™ DNA Library Prep Kit (NEB) followed by sequencing on an Illumina NovaSeq 6000 with 50-bp paired-end reads.

### bioChIP-seq analysis

The raw data were trimmed using Trim Galore! (v0.6.10) with default settings for paired-end data, ensuring a minimum read length of 20 bases. The trimmed reads were then aligned to the mouse reference genome mm10 using Bowtie2 (v2.3.5.1) with default settings, then filtered for a minimum mapping quality of 10 using samtools (v.1.19.2). Duplicate reads were removed using Picard (v2.27.5) (https://broadinstitute.github.io/picard/), and the resulting BAM files were indexed using samtools. Normalized bigWig files for visualization in the Integrative Genomics Viewer (IGV) were generated using deepTools, and individual peaks were called using MACS3 (v3.0.2) with default settings to specify regions of enrichment. Enriched motifs of binding loci for each clone were identified using the findMotifsGenome.pl module under HOMER (v5.1) (http://homer.ucsd.edu/homer/motif/HOMER). For visualization, RPGC- or CPM-normalized bigwig files were produced using deepTools bamCoverage (v3.5.5). The differential binding analysis was performed using DiffBind (v3.12.0) with adjusted *P* value <0.05 and absolute log2 fold change >1. Comparative analysis of ChIP-seq peaks between Gata3-L and Gata3-H clones was performed with MAnorm (Shao et al, 2012) under default settings.

### Generation of 3D blastoid

Wild-type and Gata3-L mouse ES cells were plated in 12-well plates coated with 0.1% gelatin and maintained in mouse ES cell culture medium. Gata3-H mouse ES cells were plated in a six-well plate under the same conditions. Cells were seeded at a density of 1.5 × 10⁵ cells/mL, and after 24 h of cell seeding, Dox was added for 24 h to induce Gata3 expression. The Dox concentrations were selected based on the levels required to achieve maximal expression of PE and TE markers for Gata3-L and Gata3-H cells, which were 0.13 and 0.5 µg/mL, respectively.

The generation of 3D blastoids was performed based on established protocols (Lau et al, 2023). After 24 h of induction, blastoid base media, which consists of DMEM (Gibco), 15% FBS (MilliporeSigma), 2 mM GlutaMax (Gibco), 1% Penicillin-Streptomycin (Gibco), 1% MEM non-essential amino acid (Gibco), 1 mM sodium pyruvate (Gibco), and 0.1 mM β-mercaptoethanol (MilliporeSigma) were prepared. Anti-adherence rinsing solution (Stem Cell Technologies) was added to each well of the 24-well AggreWell 400 plate (Stem Cell Technologies) according to the manufacturer’s protocol and were centrifuged at 2000×*g* for 5 min at room temperature. While the AggreWell with anti-adherence rinsing solution was incubated for at least 30 min at room temperature, a cell suspension was prepared and counted, resulting in 6000 wild-type, 6000 Gata3-L, and 20,000 Gata3-H cells seeded for each well. The cell mixture was centrifuged at 200×*g* for 3 min. During centrifugation, the anti-adherence rinsing solution was aspirated from the AggreWell and washed twice with 1 mL/well PBS for each wash.

Next, 500 µL of blastoid media base supplemented with 25 ng/mL Fgf4 (R&D Systems) and 1 µg/mL Heparin (MilliporeSigma) was added to each well. After centrifugation, the cell mixture was resuspended in 1 mL/well of blastoid base media supplemented with 25 ng/mL Fgf4 (R&D Systems), 1 µg/mL Heparin (MilliporeSigma), and 2 µM Y-27632 (Selleck Chemicals) and mixed by pipetting. Then, 1 mL of the resuspended cell pellet was added dropwise across the entire area of each well, and the AggreWell plate with cells was centrifuged at 100×*g* for 3 min at room temperature. The plate was maintained in a 37 °C incubator with 5% CO_2_, with careful handling to prevent cells from dislodging from the microwells. Blastoids were checked every day, followed by medium changes. On day 1, 1 mL of medium was slowly removed from the top of each AggreWell, then replaced with 1 mL of fresh blastoid base media gently along the side of the well to avoid disturbing the cells within each microwell. The media change occurred twice in total on day 1. Similarly, on day 2, 1 mL of medium was changed and replaced with fresh blastoid base media. On day 3, 3D blastoids were transferred to a µ-Slide eight-well plate (ibidi) using a pipette with a cut tip and imaged using a Nikon W1 Spinning Disk Confocal Microscope. Images were acquired using Nikon NIS Element Software (v6.10.01) and analyzed in ImageJ (v1.53).

### Immunofluorescence of 3D Blastoids

The immunofluorescence of 3D Blastoids was followed based on an established protocol (Lau et al, 2023). Blastoids cultured for 3 days were transferred into Eppendorf tubes precoated with anti-adherence rinsing solution. The tubes were left undisturbed for 5 min to allow the blastoids to settle. The samples were then washed three times with PBS. After the PBS was removed, 4% formaldehyde was added to each tube for fixation, followed by a 20-min incubation at room temperature. After fixation, the blastoids were washed three times with PBS-T (0.1% Tween-20 in PBS), incubating for 5 min per wash. Fixed blastoids can be stored in 1 mL of PBS-T in parafilm-sealed Eppendorf tubes at 4 °C for up to 1 week. Prior to permeabilization, PBS-T was carefully removed without disturbing the blastoids. Then, 400–500 µL of permeabilization buffer (30 µl of 10% Triton X-100 and 100 µl 1 M glycine solution in PBS, prepared fresh before permeabilization) was added to each tube, and the samples were incubated on a nutator at room temperature for 30 min. After permeabilization, the buffer was removed, and the blastoids were washed three times with 400–500 µL of PBS-T for 5 min each. The Gata6 antibody (R&D Systems) was diluted in blocking buffer (10 µL of 10% Tween-20 and 100 µL filtered FBS in 890 µL PBS, prepared fresh before immunofluorescence) at a 1:500 dilution, with a total volume of 400–500 µL per tube. PBS-T was removed, and the primary antibody solution was added. The blastoids were incubated overnight at 4 °C on a nutator. The next day, the primary antibody solution was removed, and the blastoids were washed three times with PBS-T for 5 min each at room temperature. The secondary antibody, Alexa Fluor 647 anti-Goat (Invitrogen), was diluted in blocking buffer (400–500 µL per tube) and then added. The samples were incubated on a nutator for 3 h at room temperature or overnight at 4 °C. Following incubation, the secondary antibodies were removed, and the blastoids were washed three times with PBS-T for 5 min each. Samples were captured using a Nikon W1 Spinning Disk Confocal Microscope. Samples within the same experiment were imaged under uniform microscopy settings. Images were acquired using Nikon NIS Element Software (v6.10.01) and analyzed in ImageJ (v1.53).

### Single-cell sample preparation of blastoid

3D blastoids were collected on day 3 and dissociated into single cells based on established protocols (Amadei et al, 2021). Blastoids were transferred to a Falcon tube, centrifuged, and washed with PBS, then incubated in TrypLE Express (Gibco) at 37 °C for 20 min. For complete disassociation, aggregates were subjected to vigorous pipetting every 5 min. Cells were subsequently centrifuged and resuspended in 1% BSA in PBS for FACS sorting of WT, Gata3-L, and Gata3-H populations based on distinct fluorescent markers (mTagBFP2, mCherry, and GFP, respectively). Sorted cells were collected in 0.5% BSA in PBS supplemented with 1 mM EDTA, followed by sample preparation using the Fixation of Cells & Nuclei for GEM-X Flex Gene Expression (10X Genomics). Sequencing was performed at UT Austin Genomic Sequencing and Analysis Facility (GSAF).

### scRNA-seq analysis

Preprocessed single-cell gene expression data were provided by 10X Genomics, generated using Cell Ranger, aligned to the mm10 reference genome. Data were normalized using NormalizeData in Seurat (v5.3.1) (R), and dimensionality reduction was performed using UMAP. Downstream analysis and visualization were conducted in both Loupe Browser v9.1 (10X Genomics) and Seurat.

### Dataset used for analysis

The previously published ChIP-seq datasets of XEN cells with Gata6 and Sox17 occupancy were obtained from GSE213661. The ChIP-seq data for Gata6-overexpressing ES cells were sourced from GSE69323. The ATAC-seq datasets for mouse ES cells were obtained from GSE220724 and GSE175632, while those for mouse TS cells were from GSE175632, and XEN cells were also sourced from GSE213661. ATAC-seq datasets for Gata6-overexpressing ES cells were obtained from GSE181104. Public scRNA-seq datasets, GSE241463 for mouse E4.5 blastocysts and GSE109555 for human embryos at 6 to 8 days post-fertilization, were also used.

## Supplementary information

Table EV1

Table EV2

Peer Review File

Source data Fig. 1

Source data Fig. 2

Source data Fig. 3

Source data Fig. 4

Source data Fig. 5

EV Figures Source Data

Expanded View Figures

## Data Availability

Raw and processed RNA-seq, ChIP-seq, and scRNA-seq data generated in this study have been deposited in the GEO database under SuperSeries accession number GSE306026, which comprises subseries GSE306024, GSE306025, and GSE327389. [SuperSeries] GSE306026: https://www.ncbi.nlm.nih.gov/geo/query/acc.cgi?acc=GSE306026, [SubSeries; RNA-seq] GSE306024: https://www.ncbi.nlm.nih.gov/geo/query/acc.cgi?acc=GSE306024, [SubSeries; ChIP-seq] GSE306025: https://www.ncbi.nlm.nih.gov/geo/query/acc.cgi?acc=GSE306025, [SubSeries; scRNA-seq] GSE327389: https://www.ncbi.nlm.nih.gov/geo/query/acc.cgi?acc=GSE327389. The source data of this paper are collected in the following database record: biostudies:S-SCDT-10_1038-S44319-026-00860-y.
