# Supplementary material for: Gata3 dosage governs primitive endoderm versus trophectoderm specification in embryonic stem cells
Source: EMBO Rep. 2026 Jul 6;27(16):4668–93. doi: 10.1038/s44319-026-00860-y (PMC13503713; doi:10.1038/s44319-026-00860-y)
Supplement: Supplementary file 1 [file 44319_2026_860_MOESM10_ESM.pdf]

Expanded View Figures

Figure EV1. Early differentiation dynamics of mouse ES cells in response to Gata3 overexpression.

(**A**) Growth curve of Gata3-OE ES cells (+Dox) compared to −Dox controls. Growth rate was measured on days 1, 2, 3, and 4 following treatments with 0.5 μg/mL Dox. Error bars indicate mean ± SD ($n = 3$ biological replicates). (**B**) Colony-forming assays of -Dox control and Gata3-OE (+Dox) cells. The bar graph represents the percentage of colonies with convex (round) or differentiated (flattened) morphology. Error bars indicate mean ± SD ($n = 4$ biological replicates). (**C**) Morphological changes in E14 and CJ7 mouse ES cells following Gata3 overexpression. Cells were treated with 0.5 μg/mL Dox for 2 days. Untreated cells (−Dox) were used as controls. Scale bar: 100 μm. (**D**) Expression of Gata3, Gata6 (PE), and Cdx2 (TE) in Gata3-OE E14 and CJ7 mouse ES cells. The red line indicates the expression level in the −Dox control. Error bars indicate mean ± SD ($n = 3$ biological replicates). (**E**) Protein and (**F**) relative mRNA expression levels of Gata3, Gata6 (PE), and Cdx2 (TE) in Gata3-OE cells treated with varying concentrations of Dox (0, 0.03, 0.13, 0.5, 2, 4 μg/mL) for 2 days. Dox with 0 μg/mL samples served as controls. Error bars indicate mean ± SD ($n = 3$ biological replicates). (**G**) Immunofluorescence analysis of Gata3 (green), Gata6, (gray) and Cdx2 (red) in Gata3-L and Gata3-H clones. Cells were treated with 0.5 μg/mL Dox for 2 days. −Dox samples were used as controls. Scale bars: 25 μm. (**H**) Quantification of the percentage of cells expressing only Gata6 (white), only Cdx2 (red), or both markers (orange, double) in Gata3-L ($n = 8$ biological replicates) and Gata3-H clones ($n = 10$ biological replicates). Error bars indicate mean ± SD. (**I**) Morphology of long-term induced Gata3-L and Gata3-H clones after 6 days of Dox treatment. Scale bars: 50 μm (left) and 100 μm (right). Source data are available online for this figure.

 © The Author(s)

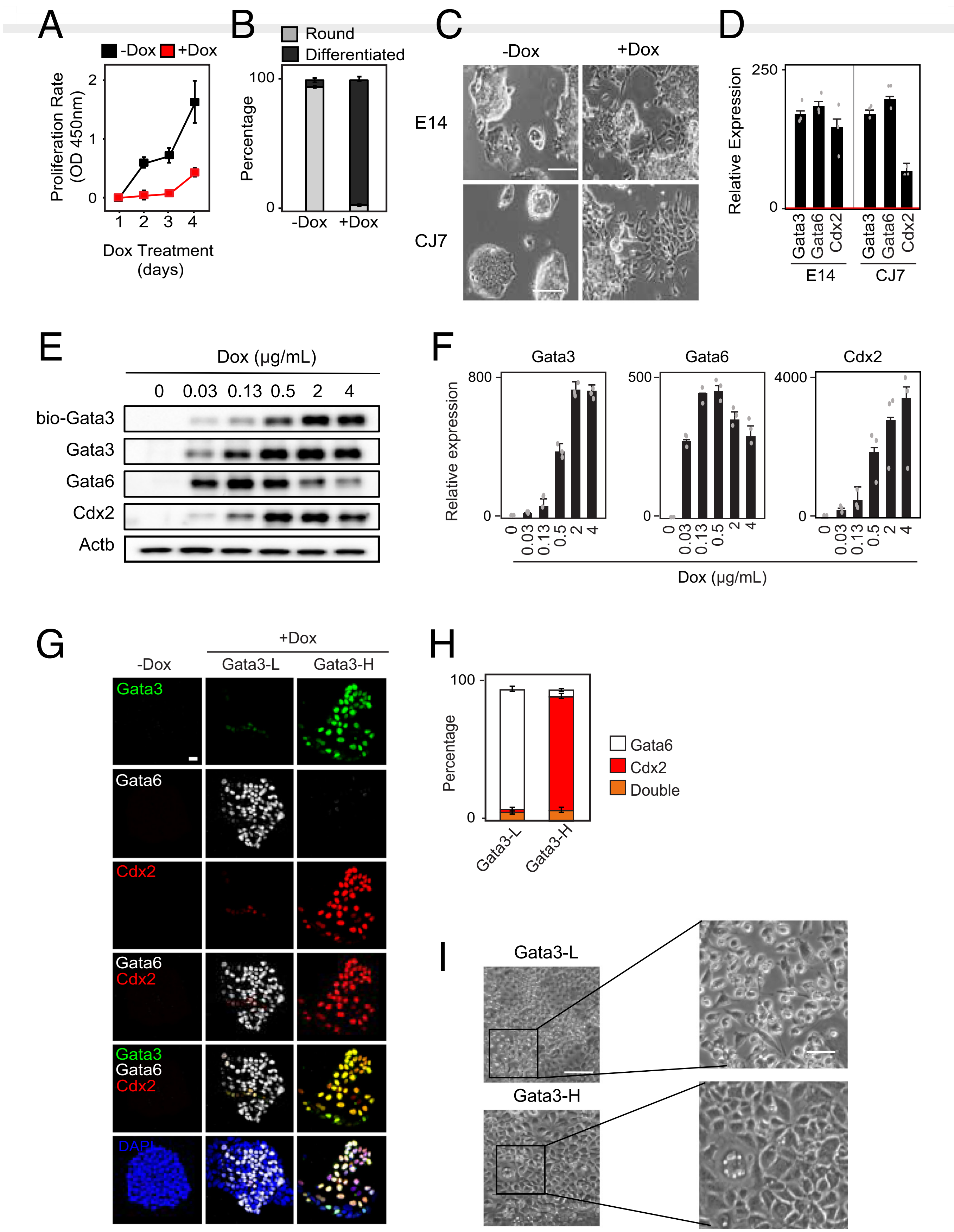
A
-Dox +Dox
Proliferation Rate (OD 450nm)
Dox Treatment (days)
B
Round
Differentiated
Percentage
-Dox +Dox
C
-Dox +Dox
E14
CJ7
D
Relative Expression
Gata3 Gata6 Cdx2 Gata3 Gata6 Cdx2
E14 CJ7
E
Dox (μg/mL)
0 0.03 0.13 0.5 2 4
bio-Gata3
Gata3
Gata6
Cdx2
Actb
F
Gata3
Gata6
Cdx2
Relative expression
Dox (μg/mL)
G
+Dox
-Dox
Gata3-L
Gata3-H
Gata3
Gata6
Cdx2
DAPI
H
Percentage
Gata6
Cdx2
Double
Gata3-L
Gata3-H
I
Gata3-L
Gata3-H

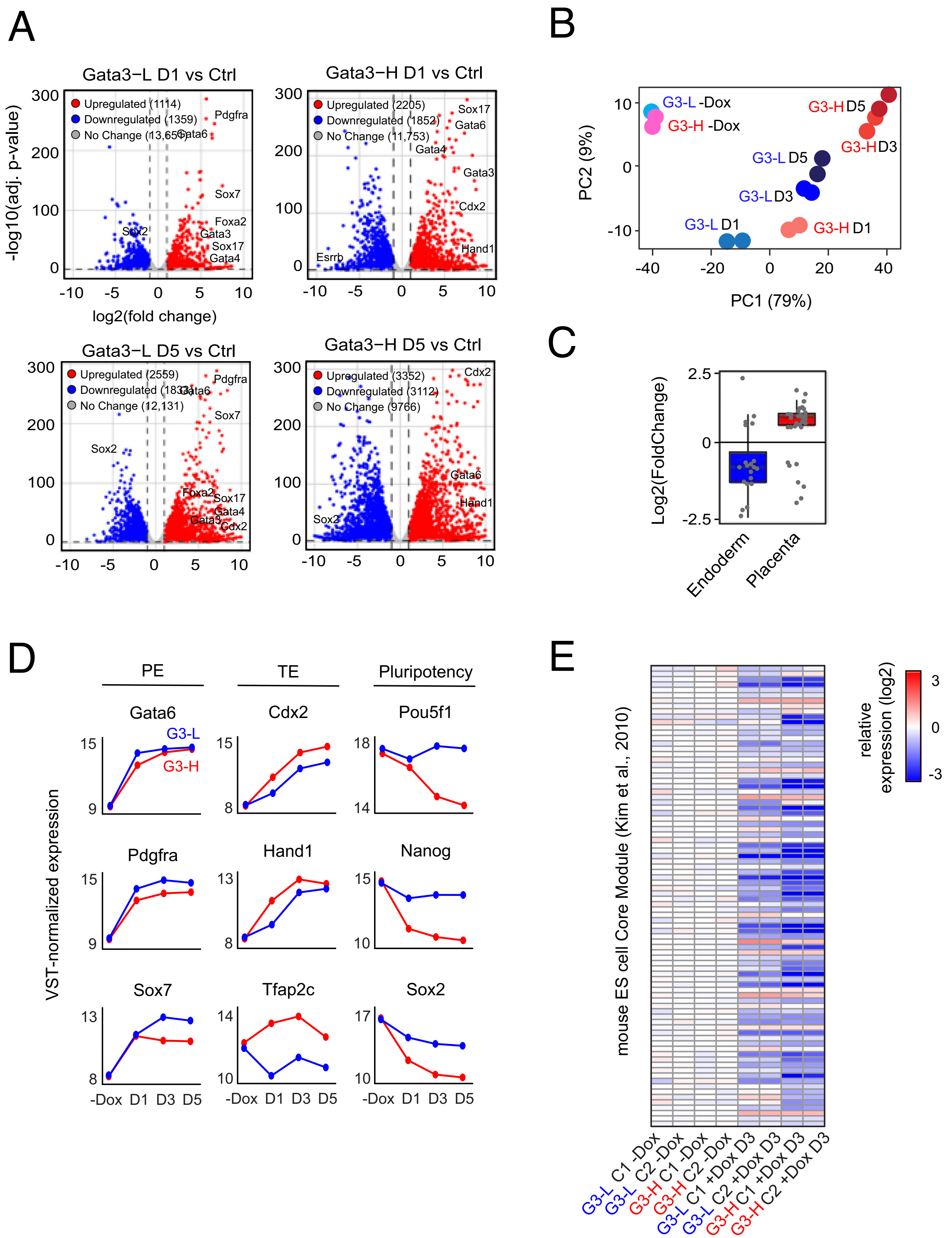

A
Gata3−L D1 vs Ctrl
Upregulated (1114)
Downregulated (1359)
No Change (13,656)
Pdgfra
Gata6
Sox7
Foxa2
Gata3
Sox17
Gata4
Sox2
-log10(adj. p-value)
log2(fold change)
Gata3−H D1 vs Ctrl
Upregulated (2205)
Downregulated (1852)
No Change (11,753)
Sox17
Gata6
Gata4
Gata3
Cdx2
Hand1
Esrrb
Gata3−L D5 vs Ctrl
Upregulated (2559)
Downregulated (1833)
No Change (12,131)
Pdgfra
Gata6
Sox7
Sox2
Foxa2
Sox17
Gata4
Gata3
Cdx2
Gata3−H D5 vs Ctrl
Upregulated (3352)
Downregulated (3112)
No Change (9766)
Cdx2
Gata6
Hand1
Sox2
B
PC2 (9%)
PC1 (79%)
G3-L -Dox
G3-H -Dox
G3-H D5
G3-H D3
G3-L D5
G3-L D3
G3-L D1
G3-H D1
C
Log2(FoldChange)
Endoderm
Placenta
D
PE
TE
Pluripotency
Gata6
Cdx2
Pou5f1
G3-L
G3-H
Pdgfra
Hand1
Nanog
Sox7
Tfap2c
Sox2
VST-normalized expression
-Dox D1 D3 D5
E
mouse ES cell Core Module (Kim et al., 2010)
relative expression (log2)
G3-L C1 -Dox
G3-L C2 -Dox
G3-H C1 -Dox
G3-H C2 -Dox
G3-L C1 +Dox D3
G3-L C2 +Dox D3
G3-H C1 +Dox D3
G3-H C2 +Dox D3

Figure EV2. Dose-dependent Gata3 induction reshapes transcriptional programs and fate-associated gene expression.

(**A**) Volcano plot showing differentially expressed genes (DEGs) between Gata3-L or Gata3-H clones and control (−Dox) after 1 and 5 days of 0.5 μg/mL Dox treatment ($n = 2$ biological replicates per condition; cutoff: $|log_2FC|>1$, adjusted P value <0.05, Wald-test with Benjamini–Hochberg correction). (**B**) Principal component analysis (PCA) illustrating the transcriptome profiles of G3-L (blue) and G3-H (red) cells after 1, 3, and 5 days of Dox treatment (0.5 μg/mL). −Dox samples were used as controls. (**C**) Comparison of log_2(Fold Change) between genes associated with endoderm and placenta, indicated in blue and red, respectively. Negative values represent upregulation in Gata3-L samples, while positive values represent upregulation in Gata3-H samples. The box plot is indicated by the center line, representing the median, the bounds of the box representing the 25th and 75th percentiles, and the whiskers representing the minimum and maximum values ($n = 2$ biological replicates per condition). (**D**) Variance-stabilizing transformed (VST) expression values of key PE, TE, and pluripotency markers in G3-L (blue) and G3-H (red) clones across a time-course of Dox treatment (0.5 μg/mL) at -Dox, 1, 3, and 5 days. (**E**) Expression patterns of the ES cell core module (Kim et al, 2010) after 3 days of induction for G3-L and G3-H samples compared to the control (−Dox). C1 and C2 represent different clones.

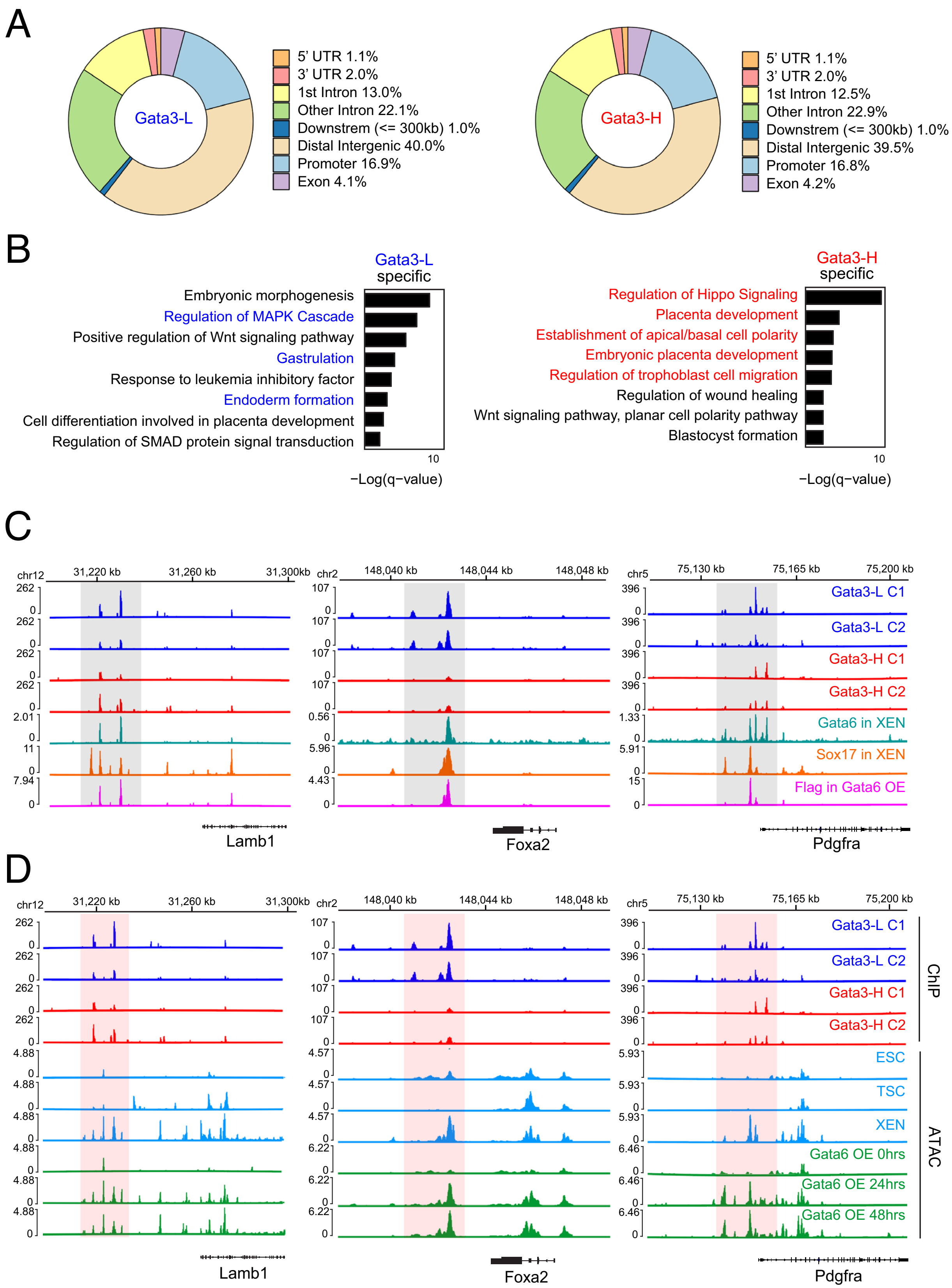
A
Gata3-L
5' UTR 1.1%
3' UTR 2.0%
1st Intron 13.0%
Other Intron 22.1%
Downstream (<= 300kb) 1.0%
Distal Intergenic 40.0%
Promoter 16.9%
Exon 4.1%
Gata3-H
5' UTR 1.1%
3' UTR 2.0%
1st Intron 12.5%
Other Intron 22.9%
Downstream (<= 300kb) 1.0%
Distal Intergenic 39.5%
Promoter 16.8%
Exon 4.2%
B
Gata3-L specific
Embryonic morphogenesis
Regulation of MAPK Cascade
Positive regulation of Wnt signaling pathway
Gastrulation
Response to leukemia inhibitory factor
Endoderm formation
Cell differentiation involved in placenta development
Regulation of SMAD protein signal transduction
10
−Log(q−value)
Gata3-H specific
Regulation of Hippo Signaling
Placenta development
Establishment of apical/basal cell polarity
Embryonic placenta development
Regulation of trophoblast cell migration
Regulation of wound healing
Wnt signaling pathway, planar cell polarity pathway
Blastocyst formation
10
−Log(q−value)
C
chr12 31,220 kb 31,260 kb 31,300kb
chr2 148,040 kb 148,044 kb 148,048 kb
chr5 75,130 kb 75,165 kb 75,200 kb
Gata3-L C1
Gata3-L C2
Gata3-H C1
Gata3-H C2
Gata6 in XEN
Sox17 in XEN
Flag in Gata6 OE
Lamb1
Foxa2
Pdgfra
D
chr12 31,220 kb 31,260 kb 31,300kb
chr2 148,040 kb 148,044 kb 148,048 kb
chr5 75,130 kb 75,165 kb 75,200 kb
Gata3-L C1
Gata3-L C2
Gata3-H C1
Gata3-H C2
ESC
TSC
XEN
Gata6 OE 0hrs
Gata6 OE 24hrs
Gata6 OE 48hrs
ChIP
ATAC
Lamb1
Foxa2
Pdgfra

 © The Author(s)

Figure EV3. Gata3 binding profiles reveal dosage-dependent functional programs and convergence with XEN chromatin at key PE loci.

(**A**) Pie charts showing the genomic distribution of Gata3 binding sites in Gata3-L and Gata3-H clones. (**B**) Enriched GO terms were identified for genes associated with Gata3-L-specific (left) and Gata3-H-specific (right) Gata3 binding regions, as defined by differential binding analysis. The top biological processes are shown, highlighting functional differences associated with low versus high Gata3 occupancy. (**C**) Genome track view showing Gata3 binding loci in Gata3-L and Gata3-H clones, along with Sox17 and Gata6 binding loci in XEN cells and Gata6-OE ES cells near key PE genes. Gray boxes indicate the highlighted regions of interest. (**D**) Genome track view showing Gata3 binding loci in Gata3-L and Gata3-H clones, together with ATAC-seq profiles of ES and TS cells, XEN cells, and Gata6-OE cells after 0, 24, and 48 h post-induction near key PE genes. Red boxes highlight regions of interest.

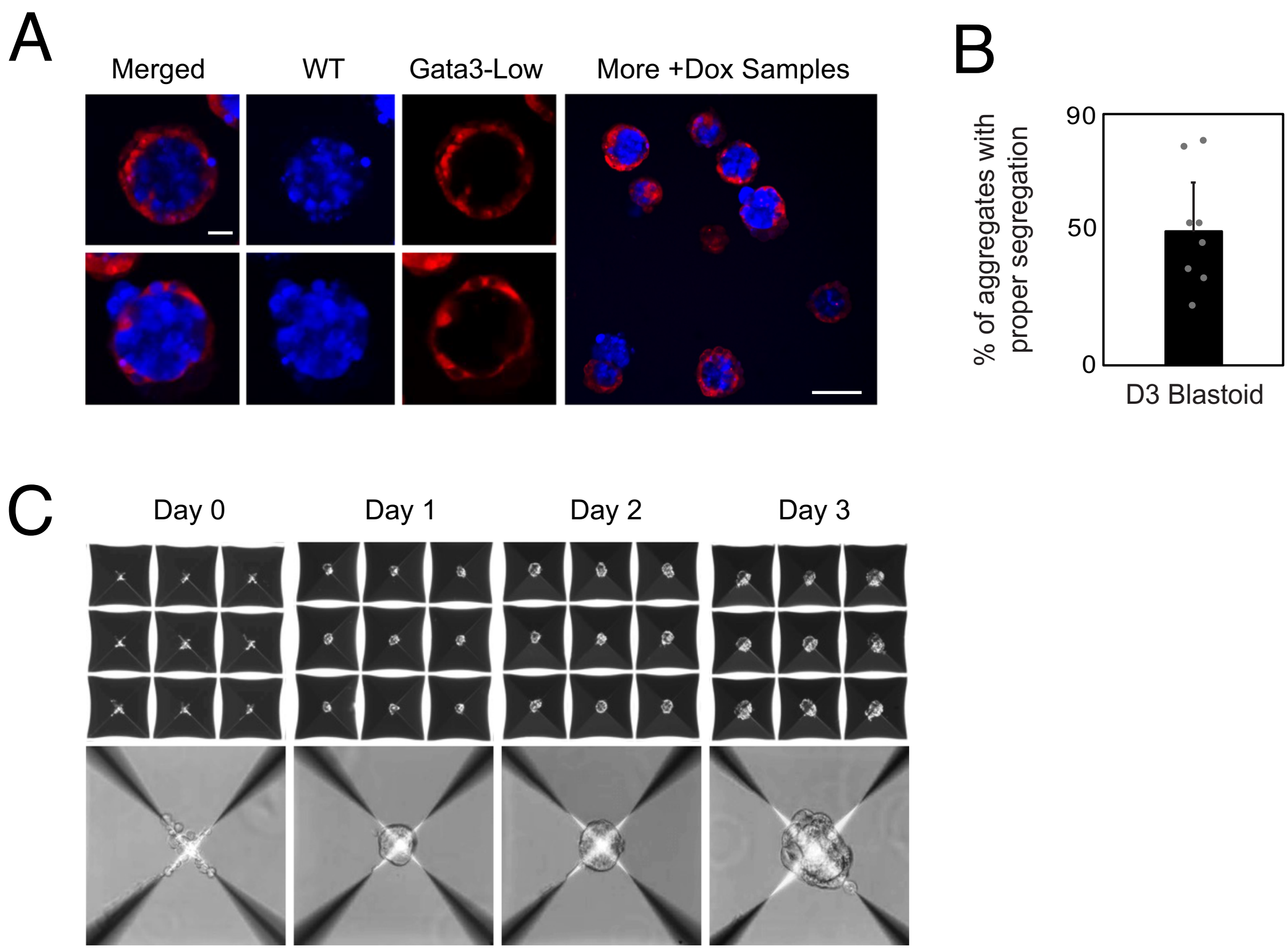

Figure EV4. Gata3 induction promotes structural organization during blastoid formation.

(**A**) Confocal images of wild-type ES cells and Gata3-L cell aggregates after 3 days of aggregation. A larger field of the +Dox samples is shown on the right. Scale bars: 20 μm (left), 100 μm (right). (**B**) Percentage of aggregates with proper segregation after 3 days of induction. Dots represent percentages from individual random fields of view. The bar indicates the mean, and the error bar represents ±SEM. $n = 8$ across two biological replicates. (**C**) Brightfield images of blastoids in an Aggrewell 400 plate throughout the culture protocol. Top: nine wells showing homogeneous blastoids. Bottom: representative image of an individual blastoid. Source data are available online for this figure.

 © The Author(s)

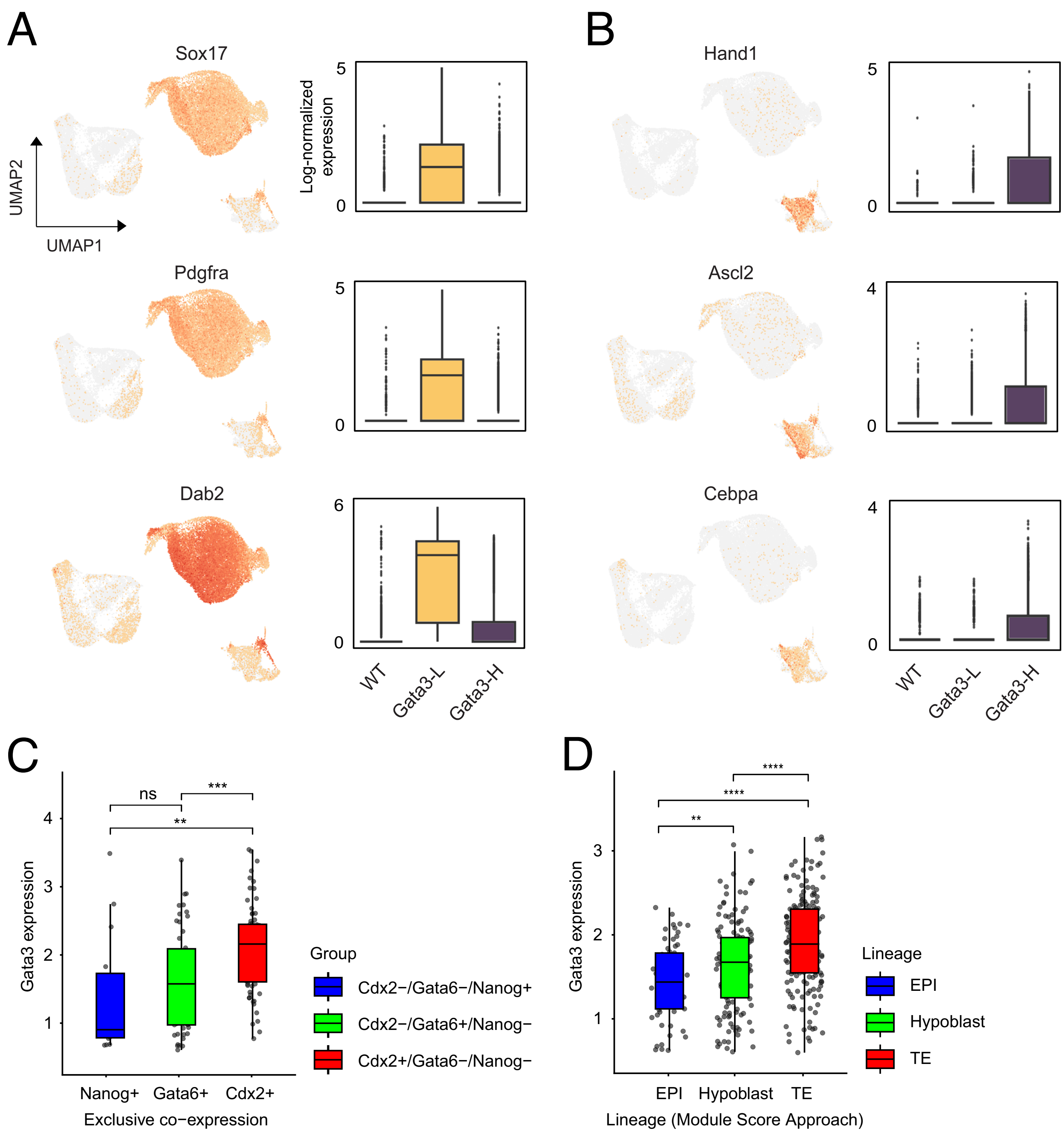
A
Sox17
Pdgfra
Dab2
UMAP2
UMAP1
Log-normalized expression
5
0
5
0
6
0
WT
Gata3-L
Gata3-H
B
Hand1
Ascl2
Cebpa
5
0
4
0
4
0
WT
Gata3-L
Gata3-H
C
ns
***
**
Gata3 expression
Group
Cdx2−/Gata6−/Nanog+
Cdx2−/Gata6+/Nanog−
Cdx2+/Gata6−/Nanog−
Nanog+
Gata6+
Cdx2+
Exclusive co−expression
D
****
****
**
Gata3 expression
Lineage
EPI
Hypoblast
TE
Lineage (Module Score Approach)

Figure EV5. Gata3-L and Gata-H clones acquire mature PE- and TE-like transcriptional programs in blastoids, recapitulating Gata3 expression patterns observed in vivo in the blastocyst.

(**A**, **B**) Expression of mature lineage-associated markers shown as UMAP feature plots and corresponding bar graph. In all box plots, the center line represents the median, the bounds of the box represent the 25th and 75th percentiles, and the dotted whiskers represent the minimum and maximum values. *n* represents the number of individual cells analyzed from a single scRNA-seq run: (**A**) PE markers (Sox17, Pdgfra, and Dab2) in Gata3-L cells, and (**B**) TE markers (Hand1, Ascl2, and Cebpa) in Gata3-H cells (WT: $n = 7948$, Gata3-L: $n = 13204$, Gata3-H: $n = 12254$). (**C**) Mouse E4.5 blastocysts scRNA-seq data, with lineage defined using exclusive expression of lineage markers. The box plot is indicated by the center line, representing the median, the bounds of the box representing the 25th and 75th percentiles, and the whiskers representing the minimum and maximum values. Data were presented as mean ± SEM. Statistical significance was determined using Student's *t*-test. The adjusted *P* values for the comparison between Nanog+ lineage ($n = 15$), Gata6+ lineage ($n = 50$) is $P = 0.36$ (ns). Significant differences were observed for Nanog+ vs. Cdx2+ ($n = 71$; $P = 0.0086$) and Gata6+ vs. Cdx2+ ($P = 0.00035$). (**D**) Human blastocyst analyzed by scRNA-seq, with lineage defined using a module score approach. The box plot is indicated by the center line, representing the median, the bounds of the box representing the 25th and 75th percentiles, and the whiskers representing the minimum and maximum values. Data were presented as mean ± SEM. Statistical significance was determined using Student's *t*-test. The adjusted *P* values are as follows: EPI ($n = 61$) vs. Hypoblast ($n = 61$), $P = 0.0096$; EPI vs. TE ($n = 140$), $P = 2.5e\text{-}9$; Hypoblast vs. TE, $P = 1.7e\text{-}5$.

 © The Author(s)
