# Supplementary material for: Gata3 dosage governs primitive endoderm versus trophectoderm specification in embryonic stem cells
Source: EMBO Rep. 2026 Jul 6;27(16):4668–93. doi: 10.1038/s44319-026-00860-y (PMC13503713; doi:10.1038/s44319-026-00860-y)
Supplement: Supplementary file 2 [file 44319_2026_860_MOESM3_ESM.pdf]

EMBO reports

Gata3 dosage governs primitive endoderm versus trophectoderm specification in embryonic stem cells

Yeejin Janga, Mijeong Kima, Joonhyuk Choia, and Jonghwan Kim

Corresponding author(s): Jonghwan Kim (jonghwankim@mail.utexas.edu)

Review Timeline:

Submission Date:	19th Sep 25
Editorial Decision:	20th Nov 25
Revision Received:	10th Apr 26
Editorial Decision:	21st May 26
Revision Received:	1st Jun 26
Accepted:	23rd Jun 26

Editor: Kurt Weir

Transaction Report:

(Note: With the exception of the correction of typographical or spelling errors that could be a source of ambiguity, letters and reports are not edited. Depending on transfer agreements, referee reports obtained elsewhere may or may not be included in this compilation. Referee reports are anonymous unless the Referee chooses to sign their reports.)

Dear Dr. Kim

Thank you for the submission of your research manuscript to our journal. We have now received the full set of referee reports that is copied below.

As you will see, the referees acknowledge that the findings are interesting and that the conclusions are overall supported by the data presented but they also raise a number of concerns and have suggestions how to further strengthen the data, particularly demonstrating differential GATA levels in vivo and greater characterization of the impact of GATA level on cell type in the blastoid model.

Given these constructive comments, we would like to invite you to revise your manuscript with the understanding that the referee concerns (as detailed above and in their reports) must be fully addressed and their suggestions taken on board. Please address all referee concerns in a complete point-by-point response. Acceptance of the manuscript will depend on a positive outcome of a second round of review. It is EMBO Reports policy to allow a single round of revision only and acceptance or rejection of the manuscript will therefore depend on the completeness of your responses included in the next, final version of the manuscript.

We realize that it is difficult to revise to a specific deadline. In the interest of protecting the conceptual advance provided by the work, we recommend a revision within 3 months (February 20th). Please discuss the revision progress ahead of this time with the editor if you require more time to complete the revisions.

I am also happy to discuss the revision further via e-mail or a video call, if you wish.

You can either publish the study as a short report or as a full article. For short reports, the revised manuscript should not exceed 27,000 characters (including spaces but excluding materials & methods and references) and 5 main plus 5 expanded view figures. The results and discussion sections must further be combined, which will help to shorten the manuscript text by eliminating some redundancy that is inevitable when discussing the same experiments twice. For a normal article there are no length limitations, but it should have more than 5 main figures and the results and discussion sections must be separate. In both cases, the entire materials and methods must be included in the main manuscript file.

================
IMPORTANT NOTE:

We perform an initial quality control of all revised manuscripts before re-review. Your manuscript will FAIL this control and the handling will be delayed IN CASE the following APPLIES:

1) A data availability section providing access to data deposited in public databases is missing. If you have not deposited any data, please add a sentence to the data availability section that explains that.

2) Your manuscript contains statistics and error bars based on n=2. Please use scatter blots in these cases. No statistics should be calculated if n=2.

When submitting your revised manuscript, please carefully review the instructions that follow below. Failure to include requested items will delay the evaluation of your revision.

================

When submitting your revised manuscript, we will require:

1) a .docx formatted version of the manuscript text (including legends for main figures, EV figures and tables). Please make sure that the changes are highlighted to be clearly visible.

2) individual production quality figure files as .eps, .tif, .jpg (one file per figure).
Please download our Figure Preparation Guidelines (figure preparation pdf) from our Author Guidelines pages https://www.embopress.org/page/journal/14693178/authorguide for more info on how to prepare your figures.

3) a .docx formatted letter INCLUDING the reviewers' reports and your detailed point-by-point responses to their comments. As part of the EMBO Press transparent editorial process, the point-by-point response is part of the Review Process File (RPF), which will be published alongside your paper.

4) a complete author checklist, which you can download from our author guidelines (). Please insert information in the checklist

that is also reflected in the manuscript. The completed author checklist will also be part of the RPF.

5) Please note that all corresponding authors are required to supply an ORCID ID for their name upon submission of a revised manuscript (). Please find instructions on how to link your ORCID ID to your account in our manuscript tracking system in our Author guidelines
()

6) We replaced Supplementary Information with Expanded View (EV) Figures and Tables that are collapsible/expandable online. A maximum of 5 EV Figures can be typeset. EV Figures should be cited as 'Figure EV1, Figure EV2" etc... in the text and their respective legends should be included in the main text after the legends of regular figures.

- For the figures that you do NOT wish to display as Expanded View figures, they should be bundled together with their legends in a single PDF file called *Appendix*, which should start with a short Table of Content. Appendix figures should be referred to in the main text as: "Appendix Figure S1, Appendix Figure S2" etc. See detailed instructions regarding expanded view here:

- Additional Tables/Datasets should be labeled and referred to as Table EV1, Dataset EV1, etc. Legends have to be provided in a separate tab in case of .xls files. Alternatively, the legend can be supplied as a separate text file (README) and zipped together with the Table/Dataset file.

7) Before submitting your revision, primary datasets (and computer code, where appropriate) produced in this study need to be deposited in an appropriate public database (see <
https://www.embopress.org/page/journal/14693178/authorguide#dataavailability>).

Please remember to provide a reviewer password if the datasets are not yet public.

The accession numbers and database should be listed in a formal "Data Availability " section (placed after Materials & Method) that follows the model below (see also < https://www.embopress.org/page/journal/14693178/authorguide#dataavailability>). Please note that the Data Availability Section is restricted to new primary data that are part of this study.

Data availability

The datasets (and computer code) produced in this study are available in the following databases:

- RNA-Seq data: Gene Expression Omnibus GSE46843 (https://www.ncbi.nlm.nih.gov/geo/query/acc.cgi?acc=GSE46843)
- [data type]: [name of the resource] [accession number/identifier/doi] ([URL or identifiers.org/DATABASE:ACCESSION])

*** Note - All links should resolve to a page where the data can be accessed. ***

8) At EMBO Press we ask authors to provide source data for the main manuscript figures. You will receive a separate email with instructions for providing source data with your revised manuscript, including how to upload and organize the files.

Additional information on source data and instruction on how to label the files are available

9) The journal requires a statement specifying whether or not authors have competing interests (defined as all potential or actual interests that could be perceived to influence the presentation or interpretation of an article). In case of competing interests, this must be specified in your disclosure statement. Further information: https://www.embopress.org/competing-interests

10) Figure legends and data quantification:
The following points must be specified in each figure legend:

- the name of the statistical test used to generate error bars and P values,
- the EXACT p-values,
- the number (n) of independent experiments (please specify technical or biological replicates) underlying each data point,
- the nature of the bars and error bars (s.d., s.e.m.)

- If the data are obtained from n {less than or equal to} 5, show the individual data points in addition to the SD or SEM.
- If the data are obtained from n {less than or equal to} 2, use scatter blots showing the individual data points.

Discussion of statistical methodology can be reported in the materials and methods section, but figure legends should contain a basic description of n, P and the test applied.

See also the guidelines for figure legend preparation:
https://www.embopress.org/page/journal/14693178/authorguide#figureformat

- Please also include scale bars in all microscopy images and define their size in the legend, not the image.

11) Our journal encourages inclusion of *data citations in the reference list* to directly cite datasets that were re-used and obtained from public databases. Data citations in the article text are distinct from normal bibliographical citations and should directly link to the database records from which the data can be accessed. In the main text, data citations are formatted as follows: "Data ref: Smith et al, 2001" or "Data ref: NCBI Sequence Read Archive PRJNA342805, 2017". In the Reference list, data citations must be labeled with "[DATASET]". A data reference must provide the database name, accession number/identifiers and a resolvable link to the landing page from which the data can be accessed at the end of the reference. Further instructions are available at .

12) All Materials and Methods need to be described in the main text using our 'Structured Methods' format. According to this format, the Methods section includes a Reagents and Tools Table (listing key reagents, experimental models, software and relevant equipment and including their sources and relevant identifiers) followed by a Methods and Protocols section describing the methods, ideally using a step-by-step protocol format. The aim is to facilitate adoption of the methodologies across labs. Please download and fill our Reagents and Tools Table template (.docx), which you can find in our author guidelines: https://www.embopress.org/page/journal/14693178/authorguide#structuredmethods.
When submitting your revised manuscript, please do not include the Reagents and Tools Table in the Methods section of the manuscript but upload it as a separate file choosing the file type "Reagent Table".
An example of a Method paper with Structured Methods can be found here: https://www.embopress.org/doi/10.15252/msb.20178071.

13) As part of the EMBO publication's Transparent Editorial Process, EMBO Reports publishes online a Review Process File to accompany accepted manuscripts. This File will be published in conjunction with your paper and will include the referee reports, your point-by-point response and all pertinent correspondence relating to the manuscript.

You are able to opt out of this by letting the editorial office know (emboreports@embo.org). If you do opt out, the Review Process File link will point to the following statement: "No Review Process File is available with this article, as the authors have chosen not to make the review process public in this case."

We would also welcome the submission of cover suggestions, or motifs to be used by our Graphics Illustrator in designing a cover.

I look forward to seeing a revised form of your manuscript when it is ready and please let me know if you have questions or comments regarding the revision.

Yours sincerely,

Kurt Weir
Editor
EMBO Reports

Referee #1:

In the study presented by Jang et al., the authors observe that varying levels of GATA3 expression in mESCs differentiated cells into primitive endoderm (PE)-like or trophectoderm (TE)-like cells, which was ascribed to the levels of GATA3 induction. By generating clones with varying levels of inducible GATA3 expression, the authors identify a dosage-sensitive response to GATA3, with GATA3 low (GATA3-L) cells preferentially adopting a PE fate while GATA3 high (GATA-H) cells adopted a TE fate. ChIP-seq performed at 2 days after induction of bio-GATA3 identified that most sites overlapped but some were unique to each clone with low GATA3 expression exhibiting higher GATA3 binding at PE distal regulatory elements, such as Gata6, while GATA3 binding in GATA3-H cells was higher at regulatory elements for genes associated with TE fate. The authors then induced GATA3 expression in the GATA3-H and GATA3-L clones for 24 hours, mixed them with wild-type ES cells and were able to recapitulate the organization of the blastoid. GATA3-L cells preferentially localized to the periphery, demonstrating that dosage-sensitive GATA3 expression in ESCs is sufficient to drive divergent cell specification and organization in vitro. Overall, this is a compelling study as it describes that transcription factor dosage can drive divergent cell fates. While that is assumed by many, there are few direct demonstrations as clean as in this study.

Comments:
1. How were the GATA3-L and GATA3-H clones generated? Were the clones selected prior to dox induction and maintained in

the pluripotent state and subsequently tested with dox induction to identify clones that induce higher expression levels of GATA3? A clearer description of this would be helpful.

2. How stable is the cell identity upon GATA3 induction in the GATA3-L and GATA3-H clones? Is a pulse (i.e., 24 hours) with treatment of dox sufficient to lead to self-sustaining TE and PE-like cell identities? Do these colonies persist in the TE or PE like state?

3. The experimental design for the blastoid formation assays would benefit from more clarity. Since it is not surprising that the cells not treated with dox at any point are unable to organize appropriately (does GATA3 expression level somehow pre-prime cells to organize or if dox was added during blastoid formation would they also organize, do they sort into the appropriate compartments during aggregation).

Along these lines, the findings underscore the cell-autonomous behavior of the GATA3-specified cells in the blastoids.

4. Do the GATA3-L and GATA3-H clones also express mature TE and PE markers upon blastoid formation? Are they functionally matured and express the relevant cell type markers?
Immunofluorescence for PE lineage markers such as Sox17, Pdgfra, Dab2 and TE markers such as Eomes, Elf5, Hand1 may help strengthen the functionality.

5. Would also be interesting to test if YAP/TAZ signaling is also implicated in the observed organization perhaps using IF for forms of activated phospho-YAP to understand if GATA3 expression levels alone act to mediate the segregation and fates in the blastoids or also act with the mechanical cues to produce functional blastoids. A minimal addition along these lines would be useful; we don't recommend extensive new work here.

6. Additional context for understanding the chromatin landscape of GATA3-L and GATA3- sites may be helpful to add mechanistic insight into how these levels may mediate targeting of chromatin that is inaccessible as detected by ATAC-Seq. Perhaps overlaying some of this data with existing ChIP-seq datasets for H3K27ac, H3K4me1, H3K9me3 in mESCs could clarify whether GATA3-L preferentially engages with cis-regulatory elements that have specific chromatin states. Useful but not absolutely necessary. There is plenty of histone mark data online that could be used to generate heatmaps addressing this question.

7. Is the GATA3 expression level also recapitulated in vivo during blastocyst formation? Does this dosage align with TE and PE cell fates?

Referee #2:

The manuscript by Yeejin Jang and co authors have showed that GATA3, in important regulator of Trophectoderm development, may also contribute to primitive endoderm development when expressed in low doses in mouse embryonic stem cells. The manuscript is interesting and could lead to a better understanding of the mechanisms of cell fate specification during early mammalian development. However, there are many aspects that should be addressed.
1. My major concern is the validation of these findings in the context of embryonic development in vivo. Is this mechanism, low level induction of GATA3 expression, relevant in the context of primitive endoderm development? If so, authors should show that GATA3 is induced at a low level during epiblast to hypoblast transition.
2. It is not clear what criteria is used to determine for high vs low doses of GATA3 level. There needs to a specific quantitation process to define that aspect, such as a comparison to the endogenous expression level in trophectoderm or trophoblast cells.
3. What is the phenotype when GATA3 is not high or not low?
4. The blastoid experiments are positive approaches. However, I am concerned about the distribution of cell types as there are mixed results. I suggest authors perform single cell RNA-seq with blastoids to better track cell fate specification through RNA-velocity or other programs.

Referee #3:

This study focuses on the dosage-dependent regulatory effect of the transcription factor Gata3 in the lineage specification of mouse embryonic stem cells (mESCs) into the primitive endoderm (PE) and trophectoderm (TE). Integrating omics analysis techniques, it clarifies that this regulatory effect is closely associated with the dose-dependent redistribution of Gata3 binding at enhancer regions and its pioneer factor-like chromatin remodeling activity.
The research defines Gata3 as a dosage-sensitive regulatory molecule that mediates the mutually exclusive differentiation of the PE and TE lineages, which supplements and extends the classical understanding of Gata3 as a TE-specific determinant. Furthermore, it provides a novel perspective on the regulatory role of transcription factor expression levels in early embryonic lineage specification.
Overall, the experimental design is logically rigorous, the technical route is standardized, the conclusions are fully supported by

experiments, and the discussion is in-depth. However, I suggest that the authors appropriately supplement validations of cell function or differentiation potential based on the analysis of differentiation marker gene expression. This additional evidence will better reinforce the core conclusion that Gata3 expression at different dosages regulates PE and TE differentiation.

Summary of additional/modified data/text in response to the reviewer's comments

Reviewer	Comment	Response
Reviewer 1	1-1	Please see our response.
	1-2	Short-term Dox treatment (pulse experiment) was performed.<br>**Response Figure 1-2**<br>Newly added as an independent section in **Materials & Methods**
	1-3	Blastocyst formation was tested under pre, post, pre/post Dox treatment conditions.<br>**Response Figure 1-3**
	1-4	**Related to 2-4**. scRNA-seq was performed.<br>**Response Figure 1-4**<br>Newly added to main results (**Fig. 5A-C** and **Fig. EV5A,B**)
	1-5	IF and Verteporfin treatment were performed<br>**Response Figure 1-5**
	1-6	H3K27ac, H3K4me1, and H3K9me3 datasets were analyzed.<br>**Response Figure 1-6**
	1-7	**Related to 2-1**. Publicly available scRNA-seq datasets from mouse and human embryos were analyzed.<br>**Response Figure 1-7**<br>Newly added to discussion section (**Fig. EV5C,D**)
Reviewer 2	2-1	**Related to 1-7**.<br>See our response and **Response Figure 1-7**<br>Newly added **Fig. EV5C,D**
	2-2	RT-qPCR was performed.<br>**Response Figure 2-2**<br>Newly added as an independent section in **Materials & Methods**
	2-3	Clone morphologies were monitored.<br>**Response Figure 2-3**
	2-4	**Related to 1-4**. scRNA-seq was performed.<br>See **Response Figures 1-4** and **2-4**<br>Newly added to main results (**Fig. 5D**)
Reviewer 3	3-1	Differentiation potential was tested in combination with XEN and TS cell culture conditions.<br>**Response Figure 3-1**

Overall Response:

We thank the Editor and reviewers for their thoughtful and constructive feedback. We are encouraged that the conceptual framework and experimental approach were well received. In response, we have revised the manuscript and incorporated additional analyses and experiments to address all concerns.

We clarified the generation and characterization of inducible Gata3 clonal lines and examined lineage stability, showing that sustained Gata3 expression is required to maintain lineage-associated transcriptional programs. We refined the blastoid assays by testing induction timing, demonstrating that both initial priming and continuous Gata3 expression are necessary for proper spatial organization.

To address lineage heterogeneity and maturation, we performed single-cell RNA-seq on blastoid-derived cells. These analyses show that Gata3 expression levels stratify within populations and correlate with PE-like and TE-like transcriptional programs. We further validated these findings using differentiation assays under PE- and TE-promoting conditions, supporting a dosage-dependent bias in lineage-associated programs.

To evaluate in vivo relevance, we analyzed published mouse and human embryo datasets and found that Gata3/GATA3 expression is highest in TE but remains detectable at lower levels in PE/hypoblast populations, consistent with our in vitro observations. We also provide additional analyses of chromatin context and YAP/TAZ signaling to further support our conclusions.

Together, these revisions strengthen our conclusion that Gata3 dosage modulates lineage-associated transcriptional programs and spatial organization, providing a framework for understanding transcription factor dosage in early lineage specification.

Referee #1:

In the study presented by Jang et al., the authors observe that varying levels of GATA3 expression in mESCs differentiated cells into primitive endoderm (PE)-like or trophectoderm (TE)-like cells, which was ascribed to the levels of GATA3 induction. By generating clones with varying levels of inducible GATA3 expression, the authors identify a dosage-sensitive response to GATA3, with GATA3 low (GATA3-L) cells preferentially adopting a PE fate while GATA3 high (GATA-H) cells adopted a TE fate. ChIP-seq performed at 2 days after induction of bio-GATA3 identified that most sites overlapped but some were unique to each clone with low GATA3 expression exhibiting higher GATA3 binding at PE distal regulatory elements, such as Gata6, while GATA3 binding in GATA3-H cells was higher at regulatory elements for genes associated with TE fate. The authors then induced GATA3 expression in the GATA3-H and GATA3-L clones for 24 hours, mixed them with wild-type ES cells and were able to recapitulate the organization of the blastoid. GATA3-L cells preferentially localized to the periphery, demonstrating that dosage-sensitive GATA3 expression in ESCs is sufficient to drive divergent cell specification and organization in vitro. Overall, this is a compelling study as it describes that transcription factor dosage can drive divergent cell fates. While that is assumed by many, there are few direct demonstrations as clean as in this study.

1-1. How were the GATA3-L and GATA3-H clones generated? Were the clones selected prior to dox induction and maintained in the pluripotent state and subsequently tested with dox induction to identify clones that induce higher expression levels of GATA3? A clearer description of this would be helpful.

Response: Gata3-overexpressing (OE) mouse ES cells were generated using a Sleeping Beauty (SB) transposon-based inducible system combined with metabolic biotinylation (FB), enabling Doxycycline

(Dox)-inducible expression of biotinylated Gata3. Following transfection, a pooled population of Gata3-OE cells was drug-selected and maintained under pluripotent conditions. Individual clones were subsequently isolated from this pool and expanded in the absence of Dox. Each clone was then induced with Dox, and Gata3 expression levels were quantified by Western blot (using both native antibody and Strep-HRP) and qPCR. Based on the magnitude of induced Gata3 expression, clones were classified as Gata3-L (low induction) or Gata3-H (high induction). Uninduced cells from each clone were maintained in parallel and cryopreserved. The observed differences in Gata3 expression levels between Gata3-L and Gata3-H clones likely reflect variation in genomic integration sites and/or transposon copy number. A detailed description has been added to the Materials and Methods section.

1-2. How stable is the cell identity upon GATA3 induction in the GATA3-L and GATA3-H clones? Is a pulse (i.e., 24 hours) with treatment of dox sufficient to lead to self-sustaining TE and PE-like cell identities? Do these colonies persist in the TE or PE like state?

Response: We appreciate the reviewer's comment on this important point. In both Gata3-L and Gata3-H clones, the acquisition of TE- and PE-like identities depends on sustained Gata3 induction. To determine whether transient expression is sufficient to stabilize these lineage programs, we performed a 24-hour Dox induction (Pulse) followed by Dox withdrawal for 0, 24, 48, and 72 hours under LIF+ conditions (**Response Figure 1-2A**). This brief induction was insufficient to establish a self-sustaining lineage program. While no obvious morphological changes were observed following pulse treatment compared to continuous induction (**Response Figure 1-2B,C**), Gata3 levels rapidly declined, accompanied by a progressive reduction in PE- and TE-associated marker expression (**Response Figure 1-2D,E**).

In contrast, continuous Dox induction maintained elevated Gata3 levels and sustained expression of lineage markers, indicating that persistent Gata3 OE supports the stabilization of the transcriptional program (**Response Figure 1-2D,E**). Notably, Gata3 expression under continuous induction peaked at approximately 48 hours and then declined. This behavior aligns with a known limitation of Dox-inducible systems, where transgene expression can be partially silenced during differentiation [1]. Nevertheless, sustained Gata3 induction was sufficient to maintain PE- and TE-like transcriptional programs, whereas transient induction was not.

Together, these results indicate that transient Gata3 induction is insufficient for the stable maintenance of lineage-specific programs, while sustained expression is required to reinforce and further maintain lineage-associated transcriptional programs.

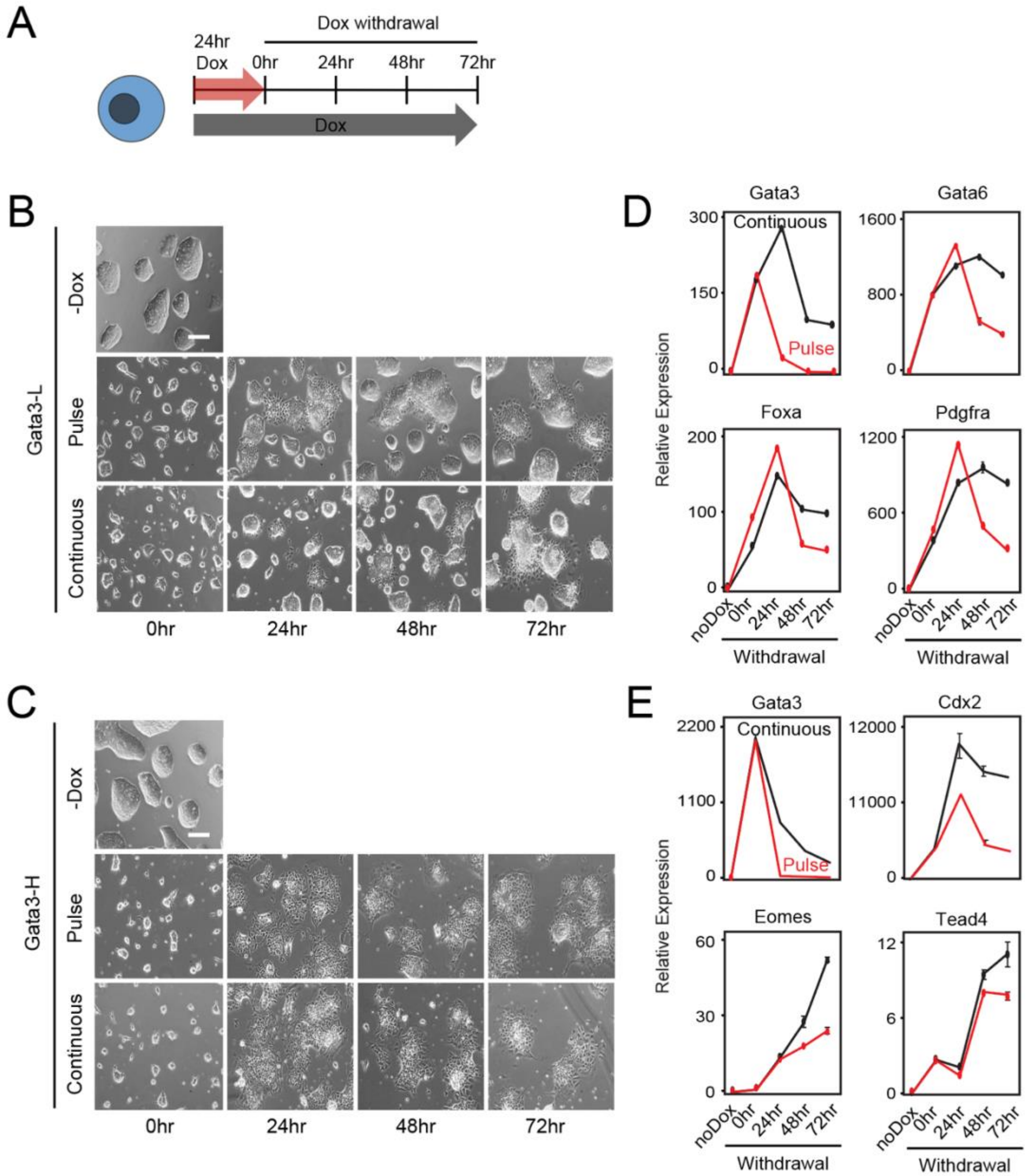

Figure 1-2. Gata3 pulse with Dox treatment is insufficient to sustain PE- and TE-like cell identities. (A) Schematic of the Gatat3 pulse treatment experiment. Cells were treated with 0.5 μg/mL Dox for 24 hours, followed by withdrawal for 0, 24, 48, 72 hours. Continuous Dox induction included as a control.
(B-C) Brightfield images of mouse ES cells overexpressing Gata3 at (B) low levels (Gata3-L) and (C) high levels (Gata3-H), comparing untreated cells (-Dox) with pulse and continuous Dox conditions. Scale bars: 100 μm.
(D-E) Relative expression levels of Gata3, (D) PE markers in Gata3-L, and (E) TE markers in Gata3-H cells following pulse (red) or continuous (black) Dox treatment after withdrawal.

1-3. The experimental design for the blastoid formation assays would benefit from more clarity. Since it is not surprising that the cells not treated with dox at any point are unable to organize appropriately (does GATA3 expression level somehow pre-prime cells to organize or if dox was added during blastoid formation would they also organize, do they sort into the appropriate compartments during aggregation).
Along these lines, the findings underscore the cell-autonomous behavior of the GATA3-specified cells in the blastoids.

Response: Our protocol was established through extensive prior optimization, including variation in cell number, induction timing and duration, and culture conditions, guided by previous blastoid studies [2, 3]. To determine whether Gata3 expression pre-primes cells or whether induction during blastoid formation alone is sufficient, we tested three induction strategies: pre-induction only, post-induction only, and combined pre- and post-induction (original method) (**Response Figure 1-3**).

In the pre-induction only condition, cells were induced with 0.125 µg/mL (Gata3-L) or 0.5 µg/mL (Gata3-H) Dox prior to aggregation, but not during blastoid culture. Although aggregates formed, they failed to organize, with Gata3-L and Gata3-H cells remaining intermixed and no properly formed blastoids observed (0%). This likely reflects a decline in Gata3 expression following Dox withdrawal at the onset of aggregation. In the post-induction only condition, 0.5 µg/mL Dox was added only during blastoid formation. Aggregates showed partial and inconsistent spatial localization, with both populations tending toward the periphery, but proper organization remained limited (9%).

In contrast, the combined pre- and post-induction condition produced reproducible spatial segregation and the highest frequency of properly organized blastoids (42%). These results indicate that both timing and duration of Gata3 induction are critical: prior induction alone is insufficient, whereas sustained induction during blastoid formation is required to maintain appropriate Gata3 levels. Consistent with Comment 1-2, where transient induction led to rapid loss of lineage-associated markers, these findings further support that continuous Gata3 expression is required.

Together, these data indicate that both initial priming and sustained induction are necessary for stable and lineage-appropriate blastoid organization.

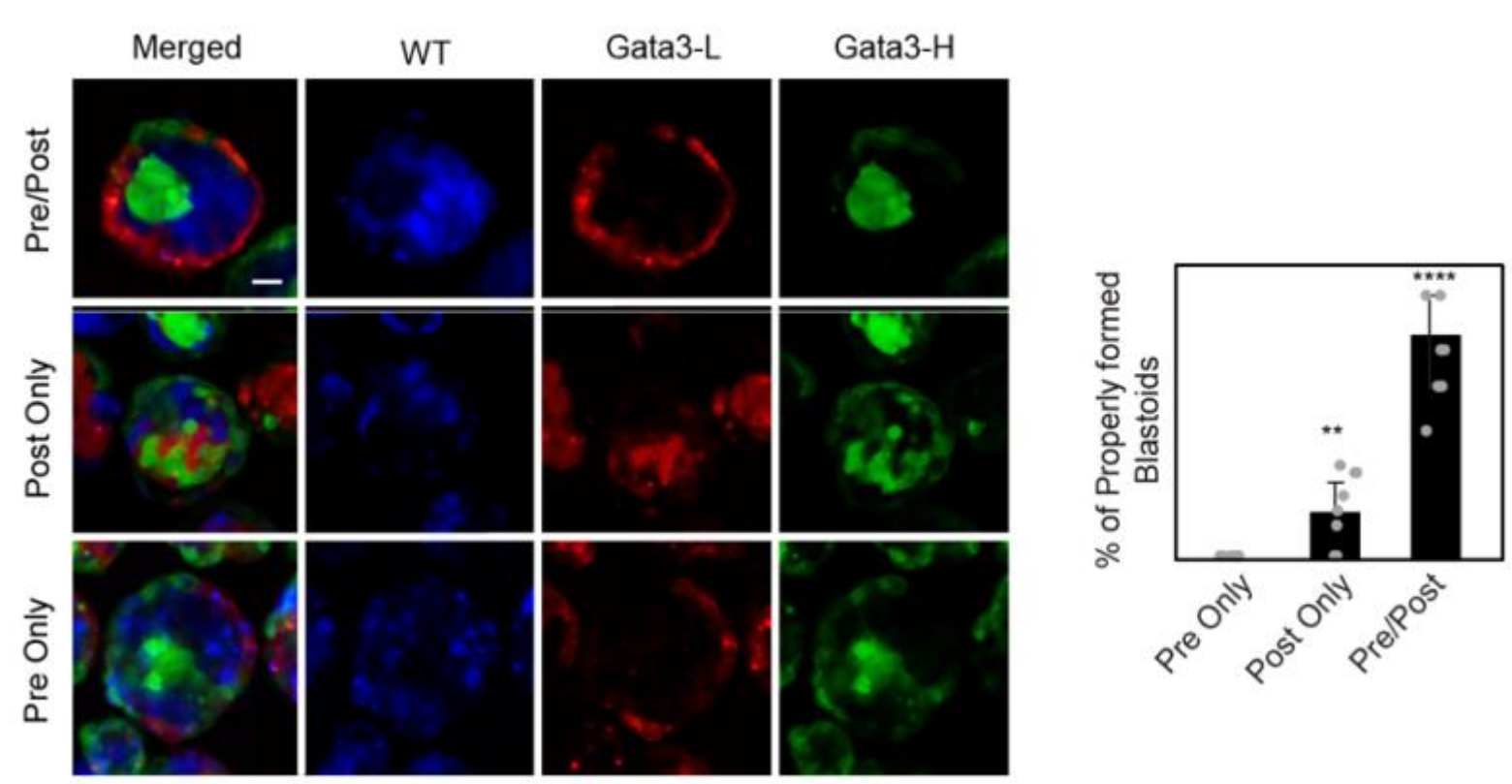

Figure 1-3. Initial priming and sustained Gata3 induction are required for proper blastoid formation. Confocal images of blastoids after 3 days of aggregation, comparing pre-induction only, post-induction only, and combined pre- and post-induction (original method). The percentage of properly developed blastoids was quantified for each condition. Dots represent percentages from individual aggregates in random fields of view, bars indicate the mean, and error bars represent ± SEM. Scale bars: 50 µm. P-values were calculated using Student's t-test. Data information: Data represent a single experiment where n = 8 random fields were quantified. Dots represent individual aggregates. Descriptive P-values are addressed: Pre-only vs. Post-only, P = 0.0020; Pre-only vs. Pre/Post, P = 2.59e-07; Post-only vs. Pre/Post, P = 1.61e-05.

1-4. Do the GATA3-L and GATA3-H clones also express mature TE and PE markers upon blastoid formation? Are they functionally matured and express the relevant cell type markers? Immunofluorescence for PE lineage markers such as Sox17, Pdgfra, Dab2 and TE markers such as Eomes, Elf5, Hand1 may help strengthen the functionality.

Response (related to Comment 2-4): While immunofluorescence could provide additional spatial validation, we performed single-cell transcriptomic analysis, as scRNA-seq provides a quantitative, transcriptome-wide assessment of lineage-associated programs; these data were also used in response to Comment 2-4.

To assess whether Gata3-L and Gata3-H clones express mature PE and TE markers upon blastoid formation, we performed scRNA-seq on dissociated day 3 blastoids (methods detailed in Comment 2-4). UMAP visualization of FACS-sorted populations (WT, Gata3-L, and Gata3-H) and unsupervised k-means clustering identified three major clusters corresponding to EPI, PE-like, and TE-like transcriptional states (**Response Figure 1-4A,B**). Although some overlap between populations was observed, clusters were predominantly enriched for WT (EPI), Gata3-L (PE-like), and Gata3-H (TE-like) cells. Importantly, Gata3 expression levels stratified across clusters, with highest expression in TE-like cells and lower levels in PE-like cells (**Response Figure 1-4C**).

To further evaluate lineage maturation, we examined established PE markers (Sox17, Pdgfra, Dab2) [4] and TE markers (Hand1, Ascl2, Cebpa) [5, 6] (**Response Figure 1-4D,E**). Both Gata3-L and Gata3-H populations contributed to these lineage-associated transcriptional programs, with enrichment consistent with their respective biases (Gata3-L toward PE-like and Gata3-H toward TE-like states). These findings indicate that Gata3 dosage promotes activation of lineage-associated transcriptional programs characteristic of more mature states within day 3 blastoids.

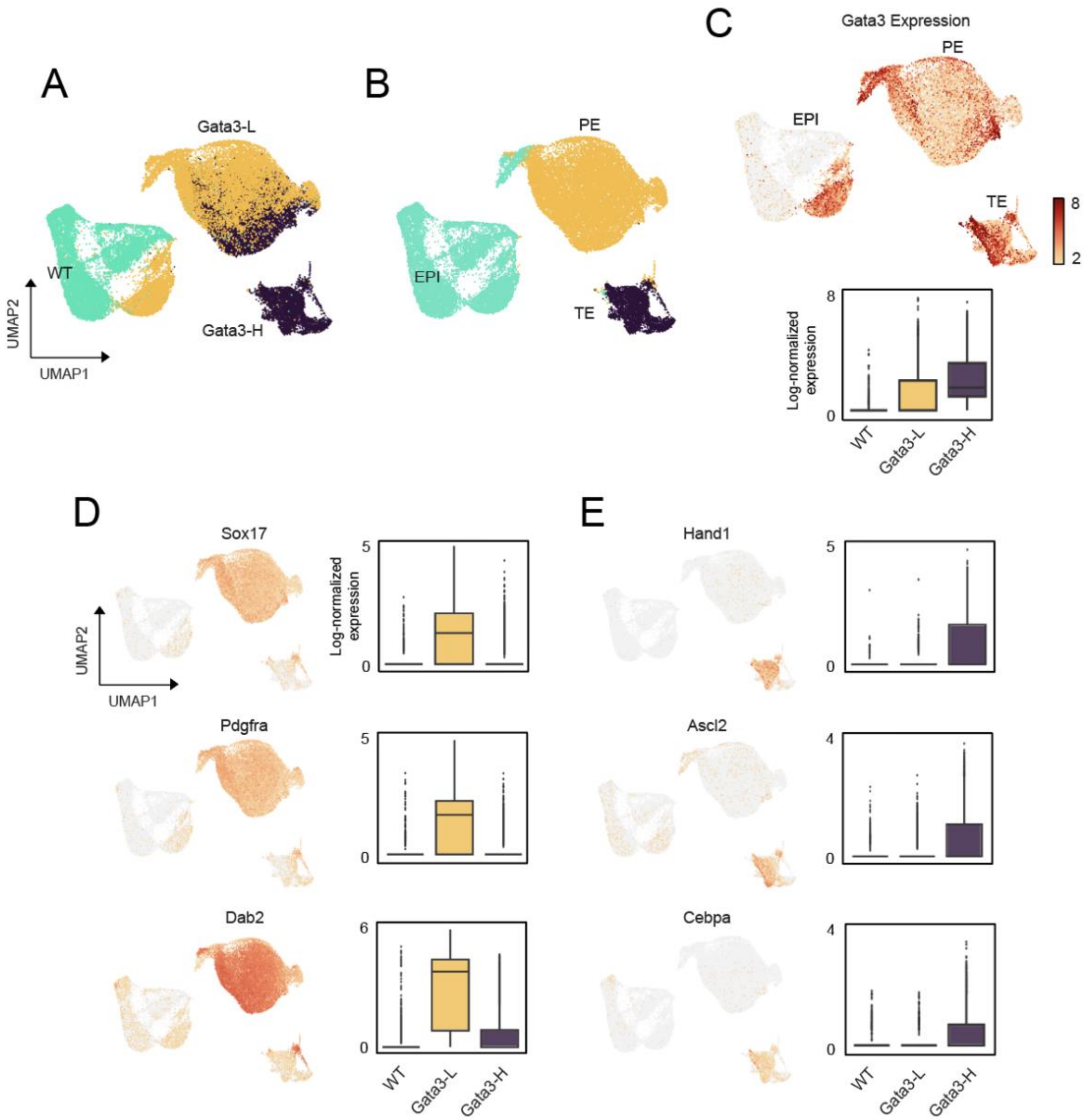

Figure 1-4. Gata3-L and Gata3-H clones acquire mature PE- and TE-like transcriptional programs in day 3 blastoids.

(A) UMAP visualization of single-cell transcriptomes from WT, Gata3-L, and Gata3-H cells disassociated from day 3 blastoids, colored by cell type. Data was visualized by 10X Genomics Loupe Browser v9.0.

(B) UMAP visualization of K-means clustering into three clusters corresponding to EPI, PE, and TE identities.

(C) Gata3 expression across clusters, showing highest expression in TE-like cells and lower expression in PE-like cells. A bar graph of log-normalized Gata3 expression across clusters is also shown.

(D-E) Expression of mature lineage-associated markers shown as UMAP feature plots and corresponding bar graph: (D) PE markers (Sox17, Pdgfra, Dab2) in Gata3-L cells, and (E) TE markers (Hand1, Ascl2, Cebpa) in Gata3-H cells.

1-5. Would also be interesting to test if YAP/TAZ signaling is also implicated in the observed organization perhaps using IF for forms of activated phospho-YAP to understand if GATA3 expression levels alone act to mediate the segregation and fates in the blastoids or also act with the mechanical cues to produce functional blastoids. A minimal addition along these lines would be useful; we don't recommend extensive new work here.

Response: Following the reviewer's suggestion, we examined the potential contribution of YAP/TAZ signaling to lineage organization in our blastoid system. Our previous study showed that Yap1 knockdown impairs differentiation into both PE and TE lineages, indicating that Yap activity can influence lineage specification [7]. Building on this, we assessed Yap localization in Gata3-L and Gata3-H clones after 2 days of induction (0.5 µg/mL Dox) by immunofluorescence. Gata3-L cells exhibited both nuclear and cytoplasmic Yap, whereas Gata3-H cells showed predominantly nuclear Yap, confirmed by nuclear/cytoplasmic (N/C) ratio analysis (**Response Figure 1-5A**).

To test whether Yap activity functionally contributes to lineage segregation, we treated day 3 blastoids with Verteporfin, a pharmacological inhibitor of YAP-TEAD activity [8, 9], across a range of concentrations. At commonly used inhibitory concentrations (0.5-1 µM), blastoids maintained normal morphology and preserved lineage-specific spatial organization. At 2 µM, a modest reduction in blastoid size was observed, consistent with Yap's role in promoting proliferation (**Response Figure 1-5B**). Higher concentrations (5-10 µM) resulted in overt cytotoxicity, including in cells cultured without Dox (**Response Figure 1-5C**), indicating nonspecific effects.

Taken together, these results indicate that although YAP/TAZ activity correlates with lineage identity, particularly increased nuclear Yap in Gata3-H cells, pharmacological inhibition of Yap at standard working concentrations does not disrupt lineage segregation. While we cannot fully exclude a contributory role, these data support Gata3 dosage as a key determinant of lineage bias and spatial organization in our tested blastoid system.

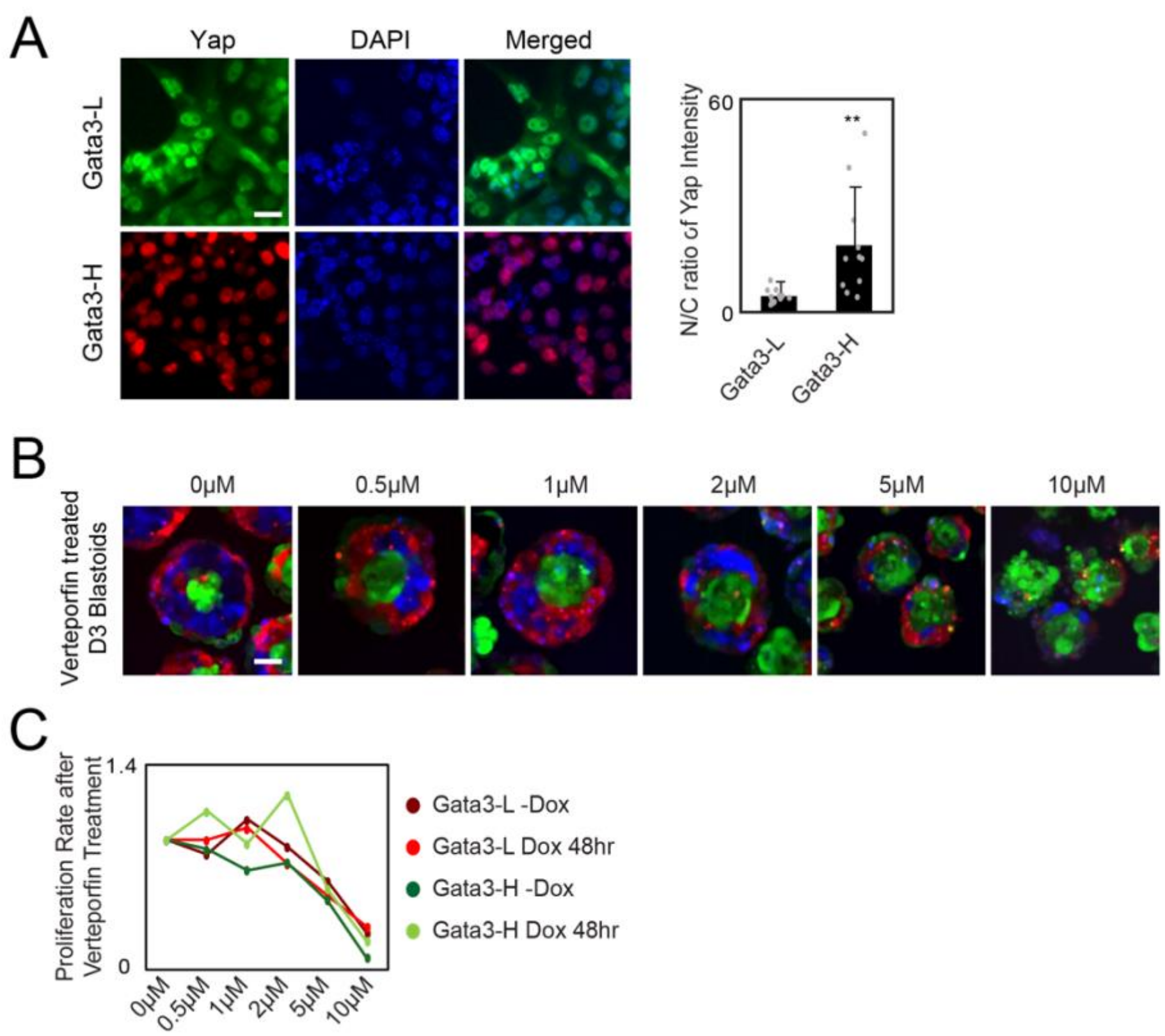

Figure 1-5. YAP/TAZ signaling correlates with lineage identity but is not required for lineage segregation in blastoids.
(A) Immunofluorescence for total Yap in Gata3-L and Gata3-H cells following 2 days of Dox induction (0.5 µg/mL) (left). Scale bars: 100 µm. Quantification of the nuclear-to-cytoplasmic (N/C) Yap ratio is shown (right). Dots represent individual fields of view (n = 12 for Gata3-L; n=11 for Gata3-H) from a single representative experiment. Bars indicate the mean ± SEM. Statistical significance was determined using Student's t-test (P = 0.0031).
(B) Confocal images of day 3 blastoids treated with increasing concentrations of Verteporfin (0, 0.5, 1, 2, 5, 10µM) throughout blastoid culture. Scale bars: 50 µm.
(C) Proliferation rate of self-renewing (-Dox) and 48-hour Dox-treated Gata3-L (red) and Gata3-H (green) cells following Verteporfin treatment across the indicated concentrations.

1-6. Additional context for understanding the chromatin landscape of GATA3-L and GATA3-H sites may be helpful to add mechanistic insight into how these levels may mediate targeting of chromatin that is inaccessible as detected by ATAC-Seq. Perhaps overlaying some of this data with existing ChIP-seq datasets for H3K27ac, H3K4me1, H3K9me3 in mESCs could clarify whether GATA3-L preferentially engages with cis-regulatory elements that have specific chromatin states. Useful but not absolutely necessary. There is plenty of histone mark data online that could be used to generate heatmaps addressing this question.

Response: To examine how different levels of Gata3 engage with the chromatin landscape, as suggested by the reviewer, we compared Gata3 occupancy with ChIP-seq datasets for H3K27ac and H3K4me1 (generated in-house) and H3K9me3 from a published dataset [10] in mouse ES cells. These signals were analyzed at Gata3-L- and Gata3-H-specific binding regions in conjunction with ATAC-seq accessibility (**Figure 3H**).

Heatmap analysis revealed that a substantial fraction of both Gata3-L- and Gata3-H-specific binding sites are located in regions lacking detectable chromatin accessibility in mouse ES cells. These regions also exhibit minimal enrichment for active (H3K27ac, H3K4me1) or repressive (H3K9me3) histone marks (**Response Figure 1-6**). Together, these results indicate that overexpressed Gata3 in ES cells can associate with chromatin regions that lack prior accessibility or canonical histone modification signatures, suggesting potential pioneer factor-like chromatin engagement.

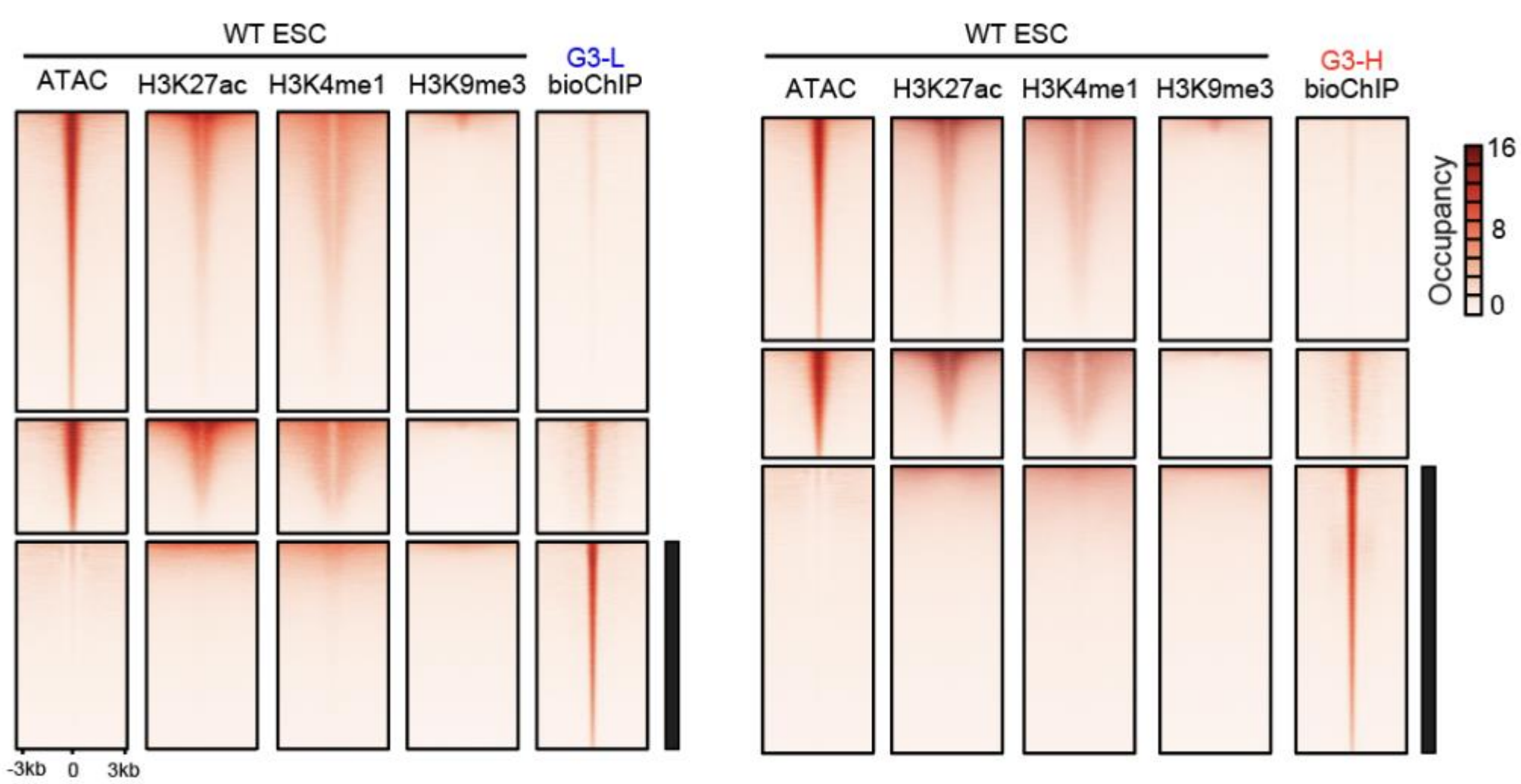

Figure 1-6. Gata3 engages silent chromatin independently of pre-existing accessibility or histone marks.

Heatmaps of ATAC-seq and ChIP-seq signals for H3K27ac, H3K4me1, and H3K9me3 in WT ES cells compared to Gata3-L and Gata3-H bioChIP peaks (±3kb from peak center). Black bars indicate regions that were closed in control ES cells but became accessible with Gata3 expression.

1-7. Is the GATA3 expression level also recapitulated in vivo during blastocyst formation? Does this dosage align with TE and PE cell fates?

Response (related to Comment 2-1): While in vitro systems do not necessarily need to fully recapitulate in vivo development, we agree that this is an important point and thank the reviewer for raising it. To assess whether differential Gata3 expression associated with lineage specification in our ES cell system is reflected in vivo, we analyzed publicly available scRNA-seq datasets from mouse [11] and human embryos [12].

In mouse E4.5 blastocysts, where the three primary lineages (EPI, PE, and TE) are specified [13], we quantified Gata3 expression in lineage-defined populations based on marker expression (Cdx2-/Gata6-/Nanog+ for EPI, Cdx2-/Gata6+/Nanog- for PE, and Cdx2+/Gata6-/Nanog- for TE) (**Response Figure 1-7A**). As expected, TE cells exhibited the highest Gata3 expression. Notably, PE cells also displayed detectable, albeit lower, levels of Gata3.

For human embryo datasets for 6 to 8 days post fertilization, lineage identities were assigned using a module score approach [14, 15] with canonical markers: EPI (NANOG, POU5F1, SOX2), hypoblast (SOX17, PDGFRA, GATA6), and TE (TFAP2C, CDX2, ELF5) (**Response Figure 1-7B**). Consistent with the mouse data, GATA3 expression was highest in TE cells but remained detectable at lower levels in hypoblasts.

Together, these observations indicate that low-level Gata3/GATA3 expression is present in PE/hypoblast populations in vivo and is conserved between mouse and human. These findings are consistent with the relevance of our in vitro dosage-based framework for PE/TE lineage bias.

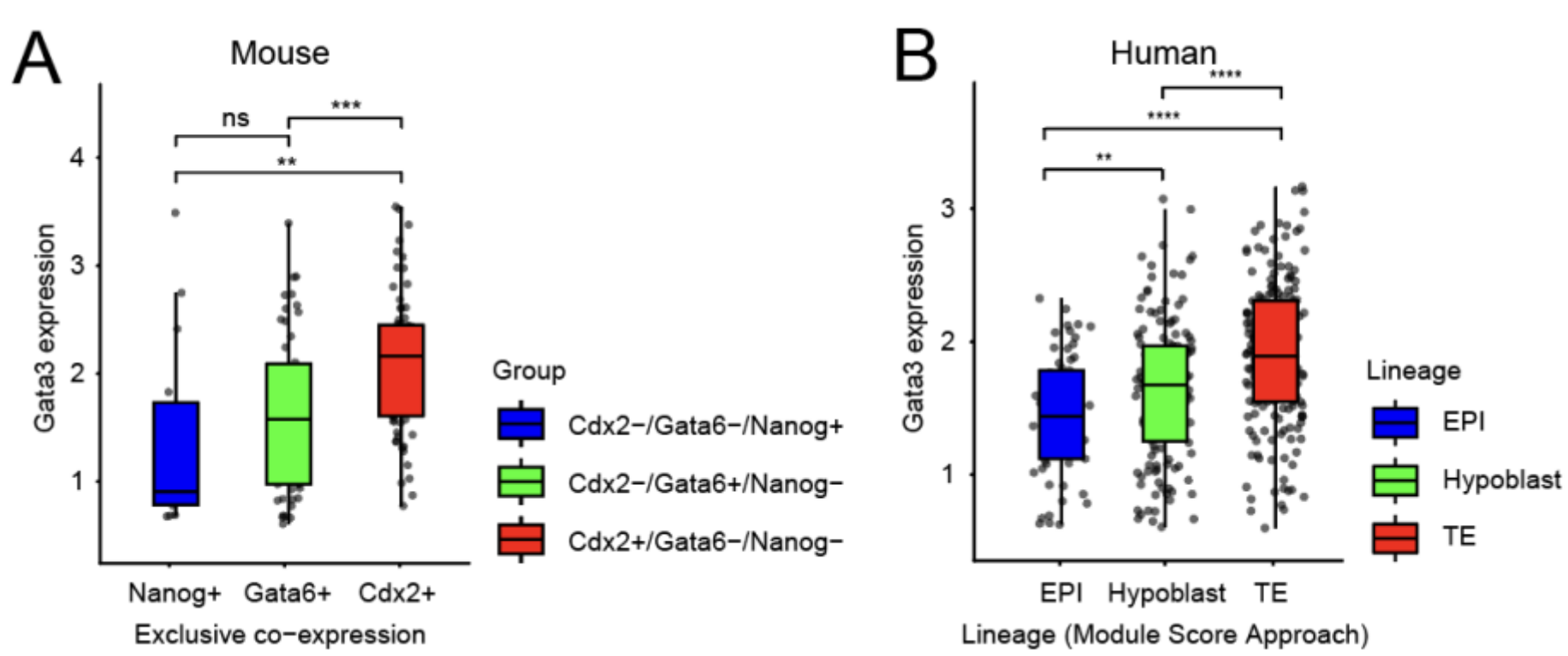

Figure 1-7. Low-level Gata3/GATA3 expression occurs in PE/hypoblast and high expression in TE in vivo.
(A) Mouse E4.5 blastocysts scRNA-seq data, with lineage defined using exclusive expression of lineage markers. Data are presented as mean ± SEM. Statistical significance was determined using Student's t-test. The adjusted P-values for the comparison between Nanog+ lineage (n = 15), Gata6+ lineage (n = 50) is P = 0.36 (ns). Significant differences were observed for Nanog+ vs. Cdx2+ (n = 71; P = 0.0086) and Gata6+ vs. Cdx2+ (P = 0.00035).
(B) Human 6-8 d.p.f. blastocysts analyzed by scRNA-seq, with lineage defined using a module score approach. Data are presented as mean ± SEM. Statistical significance was determined using Student's t-test. The adjusted P-values are as follows: EPI (n = 61) vs. Hypoblast (n = 61), P = 0.0096; EPI vs. TE (n = 140), P = 2.5e-9; Hypoblast vs. TE, P = 1.7e-5.

Referee #2:

The manuscript by Yeejin Jang and coauthors have showed that GATA3, in important regulator of Trophectoderm development, may also contribute to primitive endoderm development when expressed in low doses in mouse embryonic stem cells. The manuscript is interesting and could lead to a better understanding of the mechanisms of cell fate specification during early mammalian development. However, there are many aspects that should be addressed.

2-1. My major concern is the validation of these findings in the context of embryonic development in vivo. Is this mechanism, low level induction of GATA3 expression, relevant in the context of primitive endoderm development? If so, authors should show that GATA3 is induced at a low level during epiblast to hypoblast transition.

Response (related to Comment 1-7): We thank the reviewer for this important comment. As Comments 1-7 and 2-1 raise the same underlying issue, we address them together in our response to Comment 1-7.

2-2. It is not clear what criteria is used to determine for high vs low doses of GATA3 level. There needs to a specific quantitation process to define that aspect, such as a comparison to the endogenous expression level in trophectoderm or trophoblast cells.

Response: The Dox-inducible Sleeping Beauty system used in this study is an OE platform and is not designed to precisely replicate physiological Gata3 expression levels observed in vivo. Consequently, the goal of clone selection was not to match endogenous trophectodermal expression but to generate discrete and stable expression ranges of Gata3 within a controlled OE context.

"Low" and "High" Gata3 doses were defined based on quantitative differences in induced Gata3 expression among independent clonal lines following 48 hours of Dox treatment (0.5 µg/mL). Clones were selected from a pooled population, and the expression levels in the pooled colony served as the reference baseline (please see our response to Comment 1-1 as well). Gata3-H clones exhibited higher Gata3 expression than the pooled population, while Gata3-L clones displayed lower expression (**Response Figure 2-2A**). These clones reproducibly differed in Gata3 levels and demonstrated distinct lineage-associated transcriptional and protein profiles, supporting their functional separation.

We agree that a quantitative reference to endogenous Gata3 is important. Because direct comparisons between in vitro and in vivo cell types are challenging, we measured Gata3 mRNA in mouse trophoblast stem (TS) cells [16] and directly compared the values to our inducible clones (**Response Figure 2-2B**). This analysis revealed that even the Gata3-L clone expresses Gata3 at levels exceeding those of endogenous TS cells, consistent with the nature of the system [17]. Thus, Gata3-L and Gata3-H clones represent relatively low versus high Gata3 OE states, rather than physiological versus non-physiological thresholds.

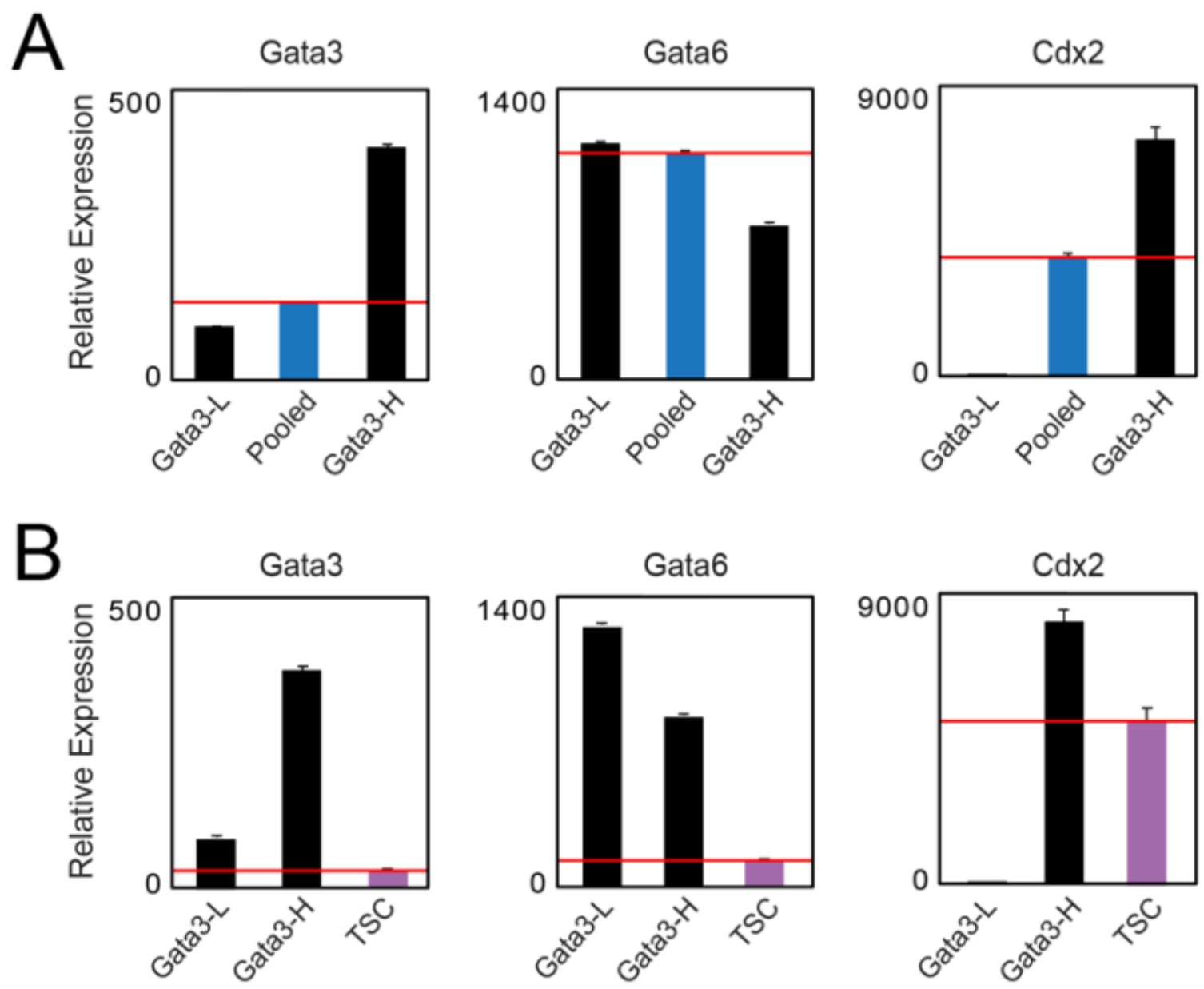

Figure 2-2. Gata3-L and Gata3-H clones define discrete overexpression states.
(A) Relative expression of Gata3, Gata6, and Cdx2 in Dox-inducible clones 48 hours after 0.5 µg/mL Dox treatment, comparing Gata3-L, Pooled, and Gata3-H population. Red line indicates the expression in the Pooled sample.
(B) Relative expression of Gata3, Gata6, and Cdx2 in inducible clones compared with endogenous expression in mouse trophoblast stem cells (TSC). Red line indicates TSC expression.

2-3. What is the phenotype when GATA3 is not high or not low?

Response: To directly address the reviewer's question regarding the phenotype at intermediate Gata3 levels, we performed a Dox dose-response analysis in pooled inducible Gata3 mouse ES cells using concentrations of 0, 0.03, 0.125, 0.5, 2, and 4 µg/mL (as shown in **Fig. EV 1E**). At low Dox concentrations (0 and 0.03 µg/mL), the cells maintained typical ES cell morphology, forming compact, round colonies. Intermediate concentrations (0.125 and 0.5 µg/mL) produced a heterogeneous population resembling the Gata3-L phenotype, with an increased proportion of flattened cells. At higher concentrations (2 and 4 µg/mL), cells were predominantly flattened and exhibited reduced proliferation, consistent with differentiation (**Response Figure 2-3A**). These results indicate that intermediate Gata3 levels do not produce a distinct intermediate morphology but instead resemble a Gata3-L-like state, with a gradual shift toward increased differentiation as expression levels rise.

To independently vary Gata3 expression, we also analyzed individual clones spanning a range of Gata3 expression levels. These clones exhibited morphologies consistent with those observed in the dose-response analysis (**Response Figure 2-3B,C**), further supporting the relationship between Gata3 levels and cell state.

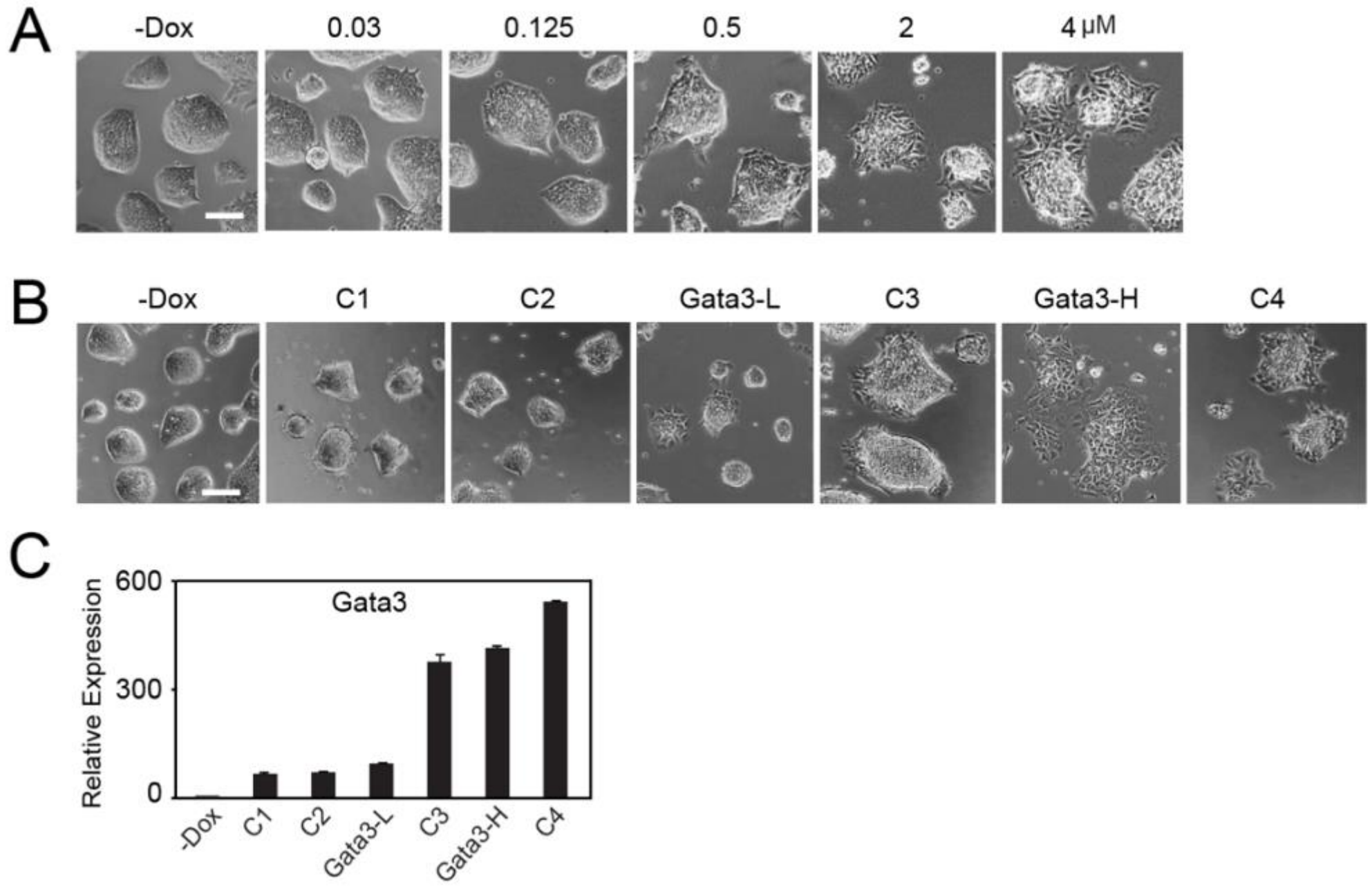

Figure 2-3. Intermediate Gata3 levels produce Gata3-L-like phenotype with increased flattened morphology.
(A) Morphology of pooled Dox-inducible Gata3 ES cells after 48 hours of Dox treatment at 0, 0.03 0.125, 0.5, 2, and 4 µg/mL. Scale bars: 100 µm.
(B) Morphology of individual clones spanning a range of Gata3 expression levels. Scale bars: 100 µm.
(C) Relative Gata3 expression in the corresponding clones.

2-4. The blastoid experiments are positive approaches. However, I am concerned about the distribution of cell types as there are mixed results. I suggest authors perform single cell RNA-seq with blastoids to better track cell fate specification through RNA-velocity or other programs.

Response (related to Comment 1-4): We agree with the reviewer that mixed cell-type distributions are observed in the blastoid experiments, which may reflect heterogeneity in Gata3 expression within the Gata3-L and Gata3-H populations, despite their clonal origin. To address this, we performed scRNA-seq on day 3 blastoids following FACS sorting of WT, Gata3-L, and Gata3-H cells using distinct fluorescent markers (mTagBFP2, mCherry, and GFP, respectively), followed by library preparation with the 10X Genomics Chromium Fixed RNA Profiling platform.

Unsupervised k-means clustering identified three major clusters (**Response Figure 1-4B**), corresponding to EPI, PE-like, and TE-like transcriptional states as determined by lineage marker expression (**Response Figure 2-4A**). While WT, Gata3-L, and Gata3-H cells were predominantly enriched in the EPI, PE-like, and TE-like clusters, respectively, some overlap was observed, including a subset of Gata3-H cells within the PE-like cluster (**Response Figure 1-4A**).

Importantly, Gata3 expression levels varied within each cluster (**Response Figure 1-4C**). Notably, a small portion of Gata3-H cells in the PE-like cluster exhibited Gata3 expression levels comparable to those of Gata3-L cells within the same cluster (**Response Figure 2-4B**). Thus, despite partial overlap, the overall relationship remained consistent: lower Gata3 expression was associated with PE-like transcriptional programs, while higher expression was associated with TE-like programs.

Consistent with this, Slingshot trajectory analysis [18] positioned WT cells at the origin, followed by Gata3-L and then Gata3-H cells along the inferred trajectory (**Response Figure 2-4C**), reflecting increasing transcriptional divergence from the EPI state [19].

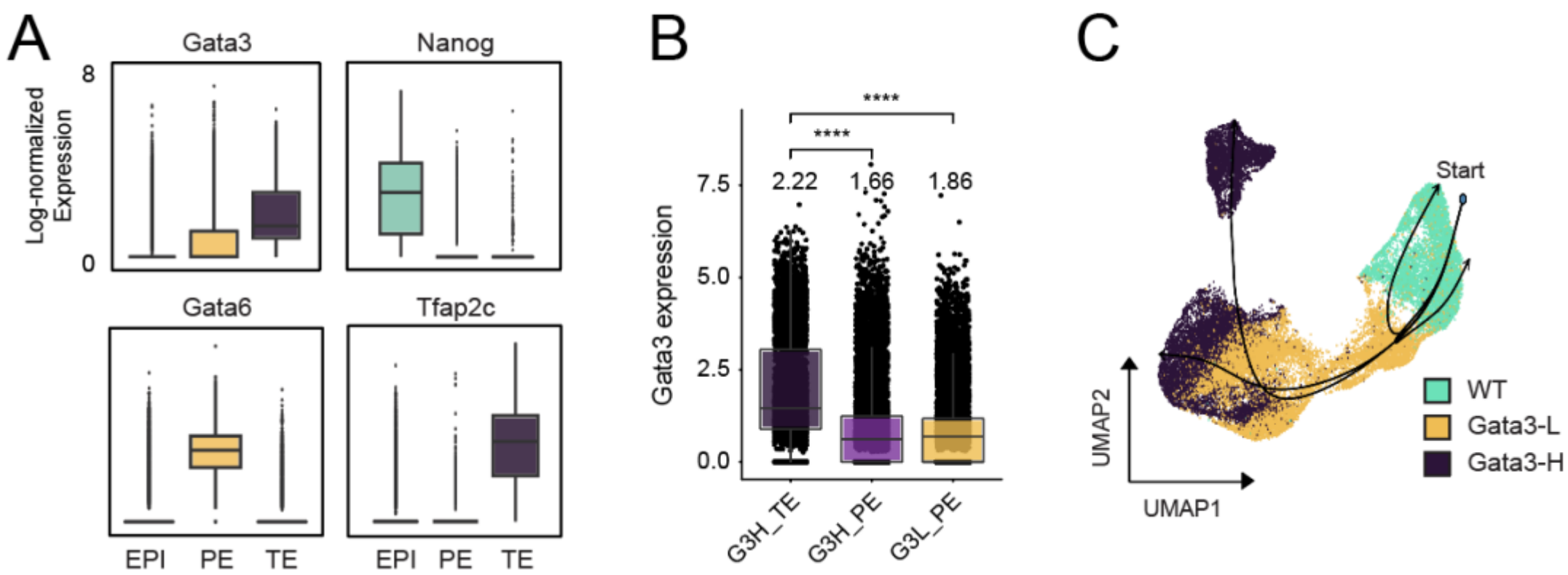

Figure 2-4. Gata3 dosage directs cell fate specification in blastoids.
(A) Cluster assignment to EPI-, PE-, and TE-like lineages based on canonical lineage marker expression.
(B) Gata3-H cells in the PE cluster express levels comparable to Gata3-L cells, reflecting partial overlap. Statistical significance was determined using Student's t-test. The adjusted P-values are as follows: G3H_TE (n = 3609) vs. G3H_PE (n = 4661), P = 6.23e-126; G3H_TE vs. G3L_PE (n = 3703), P = 3.92e-25; G3H_PE vs. G3L_PE, P = 2.4e-36.
(C) Slingshot trajectory analysis position WT cells at the origin, followed by Gata3-L and Gata3-H cells. Data was analyzed using the Seurat v5.3.1 package from R software.

Referee #3:

This study focuses on the dosage-dependent regulatory effect of the transcription factor Gata3 in the lineage specification of mouse embryonic stem cells (mESCs) into the primitive endoderm (PE) and trophectoderm (TE). Integrating omics analysis techniques, it clarifies that this regulatory effect is closely associated with the dose-dependent redistribution of Gata3 binding at enhancer regions and its pioneer factor-like chromatin remodeling activity.
The research defines Gata3 as a dosage-sensitive regulatory molecule that mediates the mutually exclusive differentiation of the PE and TE lineages, which supplements and extends the classical understanding of Gata3 as a TE-specific determinant. Furthermore, it provides a novel perspective on the regulatory role of transcription factor expression levels in early embryonic lineage specification. Overall, the experimental design is logically rigorous, the technical route is standardized, the conclusions are fully supported by experiments, and the discussion is in-depth.

3-1. However, I suggest that the authors appropriately supplement validations of cell function or differentiation potential based on the analysis of differentiation marker gene expression. This additional evidence will better reinforce the core conclusion that Gata3 expression at different dosages regulates PE and TE differentiation.

Response: To assess whether Gata3 dosage functionally biases lineage specification, we induced Gata3-L and Gata3-H clones for 3 days with Dox under pluripotent conditions, followed by differentiation in PE- or TE-promoting media (XEN or TS cell culture conditions, respectively [16, 20] for two passages (3 days each) with continuous induction (**Response Figure 3-1A**).

Under continuous Dox treatment, Gata3-L clones transitioned from compact colonies in LIF+ conditions to a more flattened, epithelial-like morphology by passage 2 under XEN conditions, consistent with a XEN-like phenotype (**Response Figure 3-1B**) [20]. This was accompanied by progressive upregulation of canonical PE-associated markers (Gata6, Gata4, Pdgfra) and endodermal genes (Sox17, Foxa2), along with downregulation of Nanog, indicating exit from pluripotency and acquisition of a PE/endoderm-like transcriptional program (**Response Figure 3-1C**).

In contrast, Gata3-H clones acquired a morphology resembling TS-like cells over time (**Response Figure 3-1B**) [16], with preferential activation of TE-associated transcription factors (Tead4, Elf5, Eomes) [21] and downstream trophoblast genes (Fosl1, Hand1) [5], alongside reduced Nanog expression (**Response Figure 3-1D**).

Together, these results indicate that sustained Gata3 expression levels bias both morphological and transcriptional programs toward PE/endoderm-like or TE-like states. These findings support a role for Gata3 dosage in modulating lineage-associated differentiation programs.

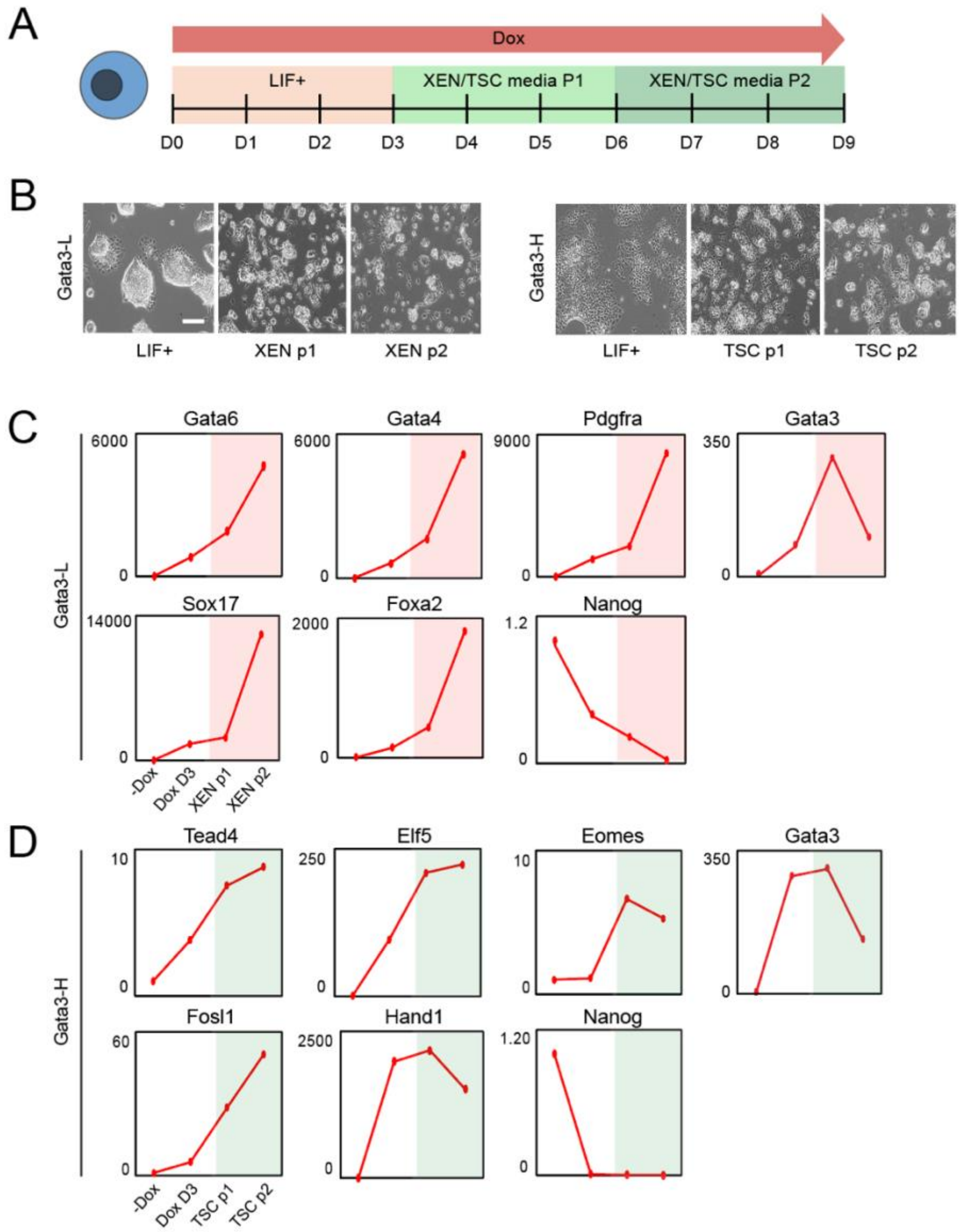

Figure 3-1. Gata3 dosage functionally biases PE versus TE differentiation.
(A) Experimental schematic of functional validation. Gata3-L and Gata3-H clones were induced for 3 days in pluripotent conditions followed by differentiation in PE- (XEN) or TE- (TSC) promoting media for two passages of 3 days each under continuous Dox induction.
(B) Morphology of Gata3-L and Gata3-H clones during differentiation. Scale bars: 100 μm.
(C) Relative expression of PE and endoderm markers. Pink box highlights growth in XEN media.
(D) Relative expression of TE markers. Green box highlights growth in TSC media.

Response References

1. Godecke, N., et al., Controlled re-activation of epigenetically silenced Tet promoter-driven transgene expression by targeted demethylation. Nucleic Acids Res, 2017. **45**(16): p. e147.
2. Lau, K.Y.C., G. Amadei, and M. Zernicka-Goetz, Assembly of complete mouse embryo models from embryonic and induced stem cell types in vitro. Nat Protoc, 2023. **18**(12): p. 3662-3689.
3. Tarazi, S., et al., Post-gastrulation synthetic embryos generated ex utero from mouse naive ESCs. Cell, 2022. **185**(18): p. 3290-3306 e25.
4. Cho, L.T., et al., Conversion from mouse embryonic to extra-embryonic endoderm stem cells reveals distinct differentiation capacities of pluripotent stem cell states. Development, 2012. **139**(16): p. 2866-77.
5. Lee, B.K., et al., Super-enhancer-guided mapping of regulatory networks controlling mouse trophoblast stem cells. Nat Commun, 2019. **10**(1): p. 4749.
6. Ueno, M., et al., c-Met-dependent multipotent labyrinth trophoblast progenitors establish placental exchange interface. Dev Cell, 2013. **27**(4): p. 373-86.
7. Chung, H., et al., Yap1 is dispensable for self-renewal but required for proper differentiation of mouse embryonic stem (ES) cells. EMBO Rep, 2016. **17**(4): p. 519-29.
8. Brodowska, K., et al., The clinically used photosensitizer Verteporfin (VP) inhibits YAP-TEAD and human retinoblastoma cell growth in vitro without light activation. Exp Eye Res, 2014. **124**: p. 67-73.
9. LeBlanc, L., et al., Yap1 safeguards mouse embryonic stem cells from excessive apoptosis during differentiation. Elife, 2018. **7**.
10. Navarro, C., et al., An embryonic stem cell-specific heterochromatin state promotes core histone exchange in the absence of DNA accessibility. Nat Commun, 2020. **11**(1): p. 5095.
11. Chen, R., et al., Analyzing embryo dormancy at single-cell resolution reveals dynamic transcriptional responses and activation of integrin-Yap/Taz prosurvival signaling. Cell Stem Cell, 2024. **31**(9): p. 1262-1279 e8.
12. Zhou, F., et al., Reconstituting the transcriptome and DNA methylome landscapes of human implantation. Nature, 2019. **572**(7771): p. 660-664.
13. Grabarek, J.B., et al., Differential plasticity of epiblast and primitive endoderm precursors within the ICM of the early mouse embryo. Development, 2012. **139**(1): p. 129-39.
14. Tirosh, I., et al., Dissecting the multicellular ecosystem of metastatic melanoma by single-cell RNA-seq. Science, 2016. **352**(6282): p. 189-96.
15. Wang, R.H. and J. Thakar, Comparative analysis of single-cell pathway scoring methods and a novel approach. NAR Genom Bioinform, 2024. **6**(3): p. lqae124.
16. Rhee, C., et al., Arid3a is essential to execution of the first cell fate decision via direct embryonic and extraembryonic transcriptional regulation. Genes Dev, 2014. **28**(20): p. 2219-32.
17. Ng, A.H.M., et al., A comprehensive library of human transcription factors for cell fate engineering. Nat Biotechnol, 2021. **39**(4): p. 510-519.

18. Street, K., et al., Slingshot: cell lineage and pseudotime inference for single-cell transcriptomics. BMC Genomics, 2018. **19**(1): p. 477.
19. Guo, G., et al., Resolution of cell fate decisions revealed by single-cell gene expression analysis from zygote to blastocyst. Dev Cell, 2010. **18**(4): p. 675-85.
20. Niakan, K.K., et al., Derivation of extraembryonic endoderm stem (XEN) cells from mouse embryos and embryonic stem cells. Nat Protoc, 2013. **8**(6): p. 1028-41.
21. Ng, R.K., et al., Epigenetic restriction of embryonic cell lineage fate by methylation of Elf5. Nat Cell Biol, 2008. **10**(11): p. 1280-90.

1st Revision - Editorial Decision **21st May 2026**

Dear Dr. Kim

Thank you for the submission of your revised manuscript to EMBO reports. We have now received the full set of referee reports that is copied below.

As you will see, all referees are very positive about the study and request no changes to clarify text and figures.

From the editorial side, there are also a few things that we need before we can proceed with the official acceptance of your study:

- As a standard procedure, we edit the abstract of manuscripts to make them more accessible to a general readership. Please find the suggested versions below my signature, which has only been adjusted to maintain present tense.

- Please remove the "This PDF file includes:" section from the manuscript.

- Please rename "Main text" from the manuscript text.

- Please rename the "Materials and methods" section to Methods.

- Please rename the "Competing Interest Statement" to Disclosure and Competing Interests Statement and place it after Acknowledgements.

- CRediT has replaced the traditional author contributions section because it offers a systematic machine readable author contributions format that allows for more effective research assessment. Please remove the Author Contributions section from the manuscript and use the free text boxes beneath each contributing author's name in our online systems to add specific details on the author's contribution. More information is available in our guide to authors.

- Please remove the section Supporting tables from the manuscript. Rename the tables to Table EV1 and Table EV2 and upload as separate files (file type Expanded View Content). The names should be updated in all places (source file names, titles in the system, legends, callouts in the manuscript).

- Please remove the Reagents and Tools table from the manuscript file and upload separately.

- Please adjust the format of the reference list and of the in-text citations according to EMBO Journal format (alphabetical order, up to 10 author names in the reference list before et al). Please refer to our Guide to Authors for additional information on EMBO J reference format.

- Please also adjust the format for citation of preprints as specified in our author guidelines:
The citation in the text should be: "(preprint: NAME1 et al, YEAR)"
The citation in the reference list: "Author NAME1, Author NAME2, ... (YEAR) article title. PLATFORM NAME, doi: XXX [PREPRINT]"

- Please include a separate citation for publicly-available datasets that were reanalyzed for this manuscript.
Please, refer to our detailed online guidelines for further information:
https://www.embopress.org/page/journal/14602075/authorguide#referencesformat

- Please note that EMBO press papers are accompanied online by:
A) a short (2 sentences) summary of the findings and their significance,
B) 2-5 short bullet points highlighting the key results, and
C) a synopsis image in .jpg or .png format that is exactly 550 pixels wide and 300 pixels high. Please note that the text needs to be legible at the final size. Please upload this information along with your revised manuscript (the text for A and B should be provided in one separate Word file uploaded as Cover Art/Synopsis Image).

- Figure EV images are pixelated under image filters. Please upload the source data for the EV blot and microscopy images.

- Thanks for providing the source data (SD). Please upload the SD file folders, which are currently all combined in a single ZIP archive, as one separate archive per each main figure. Source data files need to be saved in a scheme one figure/folder and then uploaded as .zip files. E.g. all the Source data files for figure 1 need to be saved in a single folder and this needs to be zipped and then uploaded as "SD figure 1.zip" file. For EV and/or appendix figures, ZIP together all source data.

- Please doublecheck that the source data checklist is properly filled in. In particular, please doublecheck the entries for Fig 1B,G.

- Can you explain the duplicated cells identified in the attached source data files for figures 4F-G?

- Please doublecheck that the author checklist is filled out correctly. In particular, please check the microbes entry.

- Please note that individual points should be plotted for all figures with n<5. In particular, please doublecheck Figs 1B,E,F, EV1D,F.

- Our production/data editors have asked you to clarify several points in the figure legends (see below). Please incorporate these changes in the manuscript and return the revised file with tracked changes with your final manuscript submission.
- Please note that the box plots need to be defined in terms of minima, maxima, centre, bounds of box and whiskers, and percentile in the legends of figures 5C, D; EV2 C, EV5 A-D
- Please note that information related to n is missing in the legends of figures 1B, 2B, 5C, D; EV1 A, B, D, F, H; EV2 A, C; EV5 A, B
- Please note that the error bars are not defined in the legends of figures 1B, EV1 F

Kurt Weir
Editor
EMBO Reports

Transcription factor (TF) dosage represents an overlooked aspect of developmental regulation. While Gata3 has traditionally been viewed as a determinant of trophectoderm (TE), its potential role in primitive endoderm (PE) has remained unclear. Here, we demonstrate that Gata3 functions as a dosage-sensitive regulator directing mutually exclusive lineage programs in mouse embryonic stem (ES) cells. Low levels of Gata3 (Gata3-L) promote PE-like transcriptional states, while high levels (Gata3-H) drive TE identity by rapidly repressing pluripotency and inducing TE markers. Genome-wide binding analysis reveals a dose-dependent redistribution of Gata3 across enhancers, with chromatin engagement consistent with pioneer factor-like activity. Functional 3D blastoid assays combined with single-cell transcriptomics further establish that Gata3 dosage alone is sufficient to instruct the spatial segregation of PE- versus TE-like compartments. These findings redefine Gata3 not merely as a TE determinant but as a central dosage-sensitive switch in lineage specification. More broadly, our results position TF dosage as a fundamental regulatory parameter that integrates enhancer selection, chromatin engagement, and spatial patterning, providing new opportunities to refine stem cell-based models and engineer developmental outcomes.

Referee #1:

Authors did a good job of adding scRNA seq data to improve the paper. Accept.

Referee #2:

The revised manuscript addresses all my concerns. I have no more comments.

The authors have addressed all minor editorial requests.

Jonghwan Kim
University of Texas at Austin
Molecular Biosciences
2506 Speedway
NMS 4.246
Austin, Texas 78712
United States

Dear Dr. Kim,

I am pleased to inform you that your manuscript has been accepted for publication in EMBO reports. Your manuscript will be processed for publication by EMBO Press. It will be copy edited and you will receive page proofs prior to publication. Please note that you will be contacted by Springer Nature Author Services to complete licensing and payment information.

You may qualify for financial assistance for your publication charges - either via a Springer Nature fully open access agreement or an EMBO initiative. Check your eligibility: https://link.springer.com/journal/44319/how-to-publish-with-us

Should you be planning a Press Release on your article, please notify the production team via this form in order to coordinate publication and release dates: https://forms.cloud.microsoft/e/eH4A8JVP6g

If you have any questions, please do not hesitate to contact the Editorial Office. Thank you for your contribution to EMBO Reports.

Yours sincerely,

Kurt Weir
Editor
EMBO Reports

>>> Please note that it is EMBO Reports policy for the transcript of the editorial process (containing referee reports and your response letter) to be published as an online supplement to each paper. If you do NOT want this, you will need to inform the Editorial Office via email immediately. More information is available here: https://link.springer.com/partners/embo-press/editorial-policies#Peer%20review

EMBO Press Author Checklist

Corresponding Author Name: Jonghwan Kim
Journal Submitted to: EMBO Reports
Manuscript Number: EMBOR-2025-62785V2

USEFUL LINKS FOR COMPLETING THIS FORM
The EMBO Journal - Author Guidelines
EMBO Reports - Author Guidelines
Molecular Systems Biology - Author Guidelines
EMBO Molecular Medicine - Author Guidelines

Reporting Checklist for Life Science Articles (updated January
This checklist is adapted from Materials Design Analysis Reporting (MDAR) Checklist for Authors. MDAR establishes a minimum set of requirements in transparent reporting in the life sciences (see Statement of Task: 10.31222/osf.io/9sm4x). Please follow the journal's guidelines in preparing your
Please note that a copy of this checklist will be published alongside your article.

Abridged guidelines for figures
1. Data
The data shown in figures should satisfy the following conditions:
- ➞ the data were obtained and processed according to the field's best practice and are presented to reflect the results of the experiments in an accurate and unbiased manner.
- ➞ ideally, figure panels should include only measurements that are directly comparable to each other and obtained with the same assay.
- ➞ plots include clearly labeled error bars for independent experiments and sample sizes. Unless justified, error bars should not be shown for technical
- ➞ if n<5, the individual data points from each experiment should be plotted. Any statistical test employed should be justified.
- ➞ Source Data should be included to report the data underlying figures according to the guidelines set out in the authorship guidelines on Data

2. Captions
Each figure caption should contain the following information, for each panel where they are relevant:
- ➞ a specification of the experimental system investigated (eg cell line, species name).
- ➞ the assay(s) and method(s) used to carry out the reported observations and measurements.
- ➞ an explicit mention of the biological and chemical entity(ies) that are being measured.
- ➞ an explicit mention of the biological and chemical entity(ies) that are altered/varied/perturbed in a controlled manner.
- ➞ the exact sample size (n) for each experimental group/condition, given as a number, not a range;
- ➞ a description of the sample collection allowing the reader to understand whether the samples represent technical or biological replicates (including how many animals, litters, cultures, etc.).
- ➞ a statement of how many times the experiment shown was independently replicated in the laboratory.
- ➞ definitions of statistical methods and measures:
  - common tests, such as t-test (please specify whether paired vs. unpaired), simple χ2 tests, Wilcoxon and Mann-Whitney tests, can be unambiguously identified by name only, but more complex techniques should be described in the methods section;
  - are tests one-sided or two-sided?
  - are there adjustments for multiple comparisons?
  - exact statistical test results, e.g., P values = x but not P values < x;
  - definition of 'center values' as median or average;
  - definition of error bars as s.d. or s.e.m.

Please complete ALL of the questions below.
Select "Not Applicable" only when the requested information is not relevant for your study.

Materials

Newly Created Materials	Information included in the manuscript?	In which section is the information available? (Reagents and Tools Table, Materials and Methods, Figures, Data Availability Section)
New materials and reagents need to be available; do any restrictions apply?	Yes	Methods

Antibodies	Information included in the manuscript?	In which section is the information available? (Reagents and Tools Table, Materials and Methods, Figures, Data Availability Section)
For **antibodies** provide the following information:<br>- Commercial antibodies: RRID (if possible) or supplier name, catalogue number and or/clone number<br>- Non-commercial: RRID or citation	Yes	Reagents and Tools Table

DNA and RNA sequences	Information included in the manuscript?	In which section is the information available? (Reagents and Tools Table, Materials and Methods, Figures, Data Availability Section)
**Short novel DNA or RNA including primers, probes**: provide the sequences.	Yes	Expanded View Content

Cell materials	Information included in the manuscript?	In which section is the information available? (Reagents and Tools Table, Materials and Methods, Figures, Data Availability Section)
**Cell lines:** Provide species information, strain. Provide accession number in repository **OR** supplier name, catalog number, clone number, and/**OR** RRID.	Yes	Reagents and Tools Table
**Primary cultures:** Provide species, strain, sex of origin, genetic modification status.	Not Applicable	
Report if the cell lines were recently **authenticated** (e.g., by STR profiling) and tested for mycoplasma contamination.	Not Applicable	

Experimental animals	Information included in the manuscript?	In which section is the information available? (Reagents and Tools Table, Materials and Methods, Figures, Data Availability Section)
**Laboratory animals or Model organisms:** Provide species, strain, sex, age, genetic modification status. Provide accession number in repository **OR** supplier name, catalog number, clone number, **OR** RRID.	Not Applicable	
**Animal observed in or captured from the field:** Provide species, sex, and age where possible.	Not Applicable	
Please detail **housing and husbandry conditions**.	Not Applicable	

Plants and microbes	Information included in the manuscript?	In which section is the information available? (Reagents and Tools Table, Materials and Methods, Figures, Data Availability Section)
**Plants:** provide species and strain, ecotype and cultivar where relevant, unique accession number if available, and source (including location for collected wild specimens).	Not Applicable	
**Microbes:** provide species and strain, unique accession number if available, and source.	Not Applicable	

Human research participants	Information included in the manuscript?	In which section is the information available? (Reagents and Tools Table, Materials and Methods, Figures, Data Availability Section)
If collected and within the bounds of privacy constraints report on age, sex and gender or ethnicity for all study participants.	Not Applicable	

Core facilities	Information included in the manuscript?	In which section is the information available? (Reagents and Tools Table, Materials and Methods, Figures, Data Availability Section)
If your work benefited from core facilities, was their service mentioned in the acknowledgments section?	Yes	Acknowledgements Section of Manuscript

Design

**Study protocol**	**Information included in the manuscript?**	**In which section is the information available?** (Reagents and Tools Table, Materials and Methods, Figures, Data Availability Section)
If study protocol has been **pre-registered, provide DOI in the manuscript**. For clinical trials, provide the trial registration number **OR** cite DOI.	Not Applicable	
Report the **clinical trial registration number** (at ClinicalTrials.gov or equivalent), where applicable.	Not Applicable	

**Laboratory protocol**	**Information included in the manuscript?**	**In which section is the information available?** (Reagents and Tools Table, Materials and Methods, Figures, Data Availability Section)
Provide DOI OR other citation details if **external detailed step-by-step protocols** are available.	Yes	Methods

**Experimental study design and statistics**	**Information included in the manuscript?**	**In which section is the information available?** (Reagents and Tools Table, Materials and Methods, Figures, Data Availability Section)
Include a statement about **sample size** estimate even if no statistical methods were used.	Yes	Figure legends
Were any steps taken to minimize the effects of subjective bias when allocating animals/samples to treatment (e.g. **randomization procedure**)? If yes, have they been described?	Not Applicable	
Include a statement about **blinding** even if no blinding was done.	Yes	No blinding was done
Describe **inclusion/exclusion criteria** if samples or animals were excluded from the analysis. Were the criteria pre-established?<br><br>If sample or data points were omitted from analysis, report if this was due to attrition or intentional exclusion and provide justification.	Not Applicable	
For every figure, are **statistical tests** justified as appropriate? Do the data meet the assumptions of the tests (e.g., normal distribution)? Describe any methods used to assess it. Is there an estimate of variation within each group of data? **Is** the variance similar between the groups that are being statistically compared?	Yes	Figures and Figure legends

**Sample definition and in-laboratory replication**	**Information included in the manuscript?**	**In which section is the information available?** (Reagents and Tools Table, Materials and Methods, Figures, Data Availability Section)
In the figure legends: state number of times the experiment was **replicated** in laboratory.	Yes	Figures
In the figure legends: define whether data describe **technical or biological replicates**.	Yes	Figures

Ethics

**Ethics**	**Information included in the manuscript?**	**In which section is the information available?** (Reagents and Tools Table, Materials and Methods, Figures, Data Availability Section)
Studies involving **human participants**: State details of **authority granting ethics approval** (IRB or equivalent committee(s), provide reference number for approval.	Not Applicable	
Studies involving **human participants**: Include a statement confirming that **informed consent** was obtained from all subjects and that the experiments conformed to the principles set out in the WMA Declaration of Helsinki and the Department of Health and Human Services Belmont Report.	Not Applicable	
Studies involving **human participants:** For publication of **patient photos**, include a statement confirming that consent to publish was obtained.	Not Applicable	
Studies involving experimental **animals**: State details of **authority granting ethics approval** (IRB or equivalent committee(s), provide reference number for approval. Include a statement of compliance with ethical regulations.	Not Applicable	
Studies involving **specimen and field samples:** State if relevant **permits** obtained, provide details of authority approving study; if none were required, explain why.	Not Applicable	

**Dual Use Research of Concern (DURC)**	**Information included in the manuscript?**	**In which section is the information available?** (Reagents and Tools Table, Materials and Methods, Figures, Data Availability Section)
Could your study fall under dual use research restrictions? Please check biosecurity documents and list of **select agents and toxins** (CDC): https://www.selectagents.gov/sat/list.htm	Not Applicable	
If you used a select agent, is the security level of the lab appropriate and reported in the manuscript?	Not Applicable	
If a study is subject to dual use research of concern regulations, is the name of the **authority granting approval and reference number** for the regulatory approval provided in the manuscript?	Not Applicable	

Reporting

The MDAR framework recommends adoption of discipline-specific guidelines, established and endorsed through community initiatives. Journals have their own policy about requiring specific guidelines and recommendations to complement MDAR.

**Adherence to community standards**	**Information included in the manuscript?**	**In which section is the information available?** (Reagents and Tools Table, Materials and Methods, Figures, Data Availability Section)
State if relevant guidelines or checklists (e.g., **ICMJE, MIBBI, ARRIVE, PRISMA**) have been followed or provided.	Not Applicable	
For **tumor marker prognostic studies,** we recommend that you follow the **REMARK** reporting guidelines (see link list at top right). See author guidelines, under 'Reporting Guidelines'. Please confirm you have followed these guidelines.	Not Applicable	
For **phase II and III randomized controlled trials**, please refer to the **CONSORT** flow diagram (see link list at top right) and submit the CONSORT checklist (see link list at top right) with your submission. See author guidelines, under 'Reporting Guidelines'. Please confirm you have submitted this list.	Not Applicable	

Data Availability

**Data availability**	**Information included in the manuscript?**	**In which section is the information available?** (Reagents and Tools Table, Materials and Methods, Figures, Data Availability Section)
Have **primary datasets** been deposited according to the journal's guidelines (see 'Data Deposition' section) and the respective accession numbers provided in the Data Availability Section?	Yes	Data Availability
Were **human clinical and genomic datasets** deposited in a public access-controlled repository in accordance to ethical obligations to the patients and to the applicable consent agreement?	Not Applicable	
Are **computational models** that are central and integral to a study available without restrictions in a machine-readable form? Were the relevant accession numbers or links provided?	Not Applicable	
If publicly available data were reused, provide the respective **data citations in the reference list**.	Yes	Methods
