# Supplementary material for: Gata3 dosage governs primitive endoderm versus trophectoderm specification in embryonic stem cells
Source: EMBO Rep. 2026 Jul 6;27(16):4668–93. doi: 10.1038/s44319-026-00860-y (PMC13503713; doi:10.1038/s44319-026-00860-y)
Supplement: Supplementary file 3 [file 44319_2026_860_MOESM1_ESM.docx]

**Table EV1**. Cell type-specific gene sets

| **Endoderm-related** | **Placenta-related** | **Pluripotency** |
| --- | --- | --- |
| Angpt1 | Adm | Agtrap |
| Angpt2 | Akt1 | Anp32a |
| Angpt4 | Arnt | Asxl1 |
| Ankrd17 | Ascl2 | Rif1 |
| Arc | Birc6 | Camk2n1 |
| Bmp4 | Bmp5 | Casq2 |
| Bmpr1a | Bmp7 | Cbx1 |
| Bptf | Bptf | Cbx7 |
| Brd3 | Ccn1 | Cd38 |
| Cdc73 | Cdkn1c | Chd9 |
| Cfc1 | Cdx2 | Cobl |
| Col12a1 | Cdx4 | Crmp1 |
| Col5a1 | Cebpa | Dll1 |
| Col5a2 | Cebpb | Dst |
| Ctnnb1 | Cited1 | Dyrk3 |
| Ctr9 | Cited2 | Evx1 |
| Dkk1 | Crxos | Fbxo15 |
| Dusp1 | Csf2 | Fn1 |
| Dusp2 | Cts7 | Gja1 |
| Dusp4 | Cts8 | Gjb3 |
| Dusp5 | Dnajb6 | Gpa33 |
| Eomes | E2f7 | Gpr1 |
| Epb41l5 | E2f8 | Hoxb13 |
| Ext1 | Egfr | Ifitm1 |
| Fgf8 | Egln1 | Ifitm2 |
| Fn1 | Elf5 | Il6st |
| Foxa2 | Eomes | Itga9 |
| Gata4 | Epas1 | Jam2 |
| Gata6 | Erf | Kifc3 |
| Gdf1 | Esrrb | Klf2 |
| Gdf3 | Esx1 | Klf9 |
| Grb2 | Fbxw8 | Lefty2 |
| Hdac1 | Fgfr2 | Lrrc2 |
| Hhex | Foxd3 | Map4k4 |
| Hmga2 | Fzd5 | Mllt6 |
| Hnf1b | Gab1 | Msh6 |
| Hsbp1 | Gata2 | Mybl2 |
| Inhba | Gcm1 | Mycn |
| Itga5 | Ggnbp2 | Nanog |
| Itgav | Gjb5 | Nfkbia |
| Kif16b | Grb2 | Nodal |
| Leo1 | Grhl2 | Phc1 |
| Lhx1 | Hand1 | Pipox |
| Macroh2a1 | Hectd1 | Plscr1 |
| Map2k1 | Hes1 | Pou5f1 |
| Med12 | Hey1 | Ppp1r15b |
| Mesp1 | Hey2 | Rarg |
| Mixl1 | Hif1a | Rbbp5 |
| Myh9 | Hs6st1 | Rbm14 |
| Nanog | Hsf1 | Rest |
| Nckap1 | Igf2 | Rhof |
| Nkx2-1 | Il10 | Rps19 |
| Nkx2-5 | Itga4 | Sfrp1 |
| Nodal | Itgav | Slc20a1 |
| Nog | Junb | Socs2 |
| Notch1 | Krt19 | Socs3 |
| Nr0b1 | Krt8 | Sox13 |
| Onecut1 | Lef1 | Sox2 |
| Otx2 | Lif | Spry2 |
| Paf1 | Llgl2 | Sulf2 |
| Pax9 | Map2k1 | Tbc1d13 |
| Pelo | Mapk1 | Tbl1xr1 |
| Pou5f1 | Mdfi | Tcea3 |
| Pthlh | Med1 | Tcf3 |
| Rtf1 | Mir127 | Tdgf1 |
| Setd2 | Ncoa1 | Tmem8 |
| Smad2 | Ncoa3 | Tpd52 |
| Smad3 | Ncoa6 | Trib3 |
| Smad4 | Nfe2 | Trim25 |
| Smim43 | Nodal | Uck2 |
| Sox17 | Nr2f2 | Upp1 |
| Sox2 | Nrk | Zfp36l1 |
| Sox7 | Nsdhl | Zfp42 |
| Ssbp3 | Ovol2 | Zic2 |
| Tgfb1 | Pcdh12 | Zic5 |
| Tnrc6a | Pdgfb | 1700019D03Rik |
| Tnrc6c | Phlda2 | 2310003C23Rik |
| Vtn | Pkd1 | 2410137M14Rik |
| Wnt8a | Pkd2 | 5730559C18Rik |
| Zfp36l1 | Plac1 | 6030445D17Rik |
|  | Plcd1 | AW822216 |
|  | Plcd3 | C79267 |
|  | Plg | D11Ertd636e |
|  | Plk4 | D14Ertd436e |
|  | Prdm1 | D5Ertd585e |
|  | Rbpj | D630039A03Rik |
|  | Rspo3 | Dido1 |
|  | Rtl1 | Epb4.9 |
|  | Senp2 | Fubp3 |
|  | Setd2 | Grhl3 |
|  | Slc8a1 | Il11ra2 |
|  | Snai1 | LOC433801 |
|  | Socs3 | Lrrc34 |
|  | Sox15 | Lrrn2 |
|  | Sp1 | Mapk1ip1 |
|  | Sp3 | MGC117846 |
|  | Spint1 | OTTMUSG00000010673 |
|  | Spint2 | Prmt8 |
|  | St14 | Ptch1 |
|  | Stk3 | Raet1a |
|  | Stk4 | Reep3 |
|  | Syde1 | Rmnd5b |
|  | Syna | Slc25a36 |
|  | Synb | Tcfcp2l1 |
|  | Taf10 | Wdr79 |
|  | Tex19.1 | Zfp13 |
|  | Tfeb | Zfp206 |
|  | Tmed2 | Zfp219 |
|  | Trim28 | Zfp428 |
|  | Ttpa | Zfp57 |
|  | Vash1 | Zfp704 |
|  | Vash2 |  |
|  | Vcam1 |  |
|  | Wnt2 |  |
|  | Wnt7b |  |
|  | Xist |  |
|  | Zfat |  |
|  | Zfp36l1 |  |
|  | Zfp568 |  |
