# Supplementary material for: Gata3 dosage governs primitive endoderm versus trophectoderm specification in embryonic stem cells
Source: EMBO Rep. 2026 Jul 6;27(16):4668–93. doi: 10.1038/s44319-026-00860-y (PMC13503713; doi:10.1038/s44319-026-00860-y)
Supplement: Supplementary file 4 [file 44319_2026_860_MOESM2_ESM.docx]

**Table EV2.** Oligo sequences of RT-qPCR primers

| **Gene Name** | **Forward Primer (5'-3')** | **Reverse Primer (3'-5')** |
| --- | --- | --- |
| Gata3 | TGGGCTGTACTACAAGCTTCATAA | CTTTTTCGATTTGCTAGACATCTTC |
| Gata6 | GACGGCACCGGTCATTACC | ACAGTTGGCACAGGACAGTCC |
| Gata4 | TTCTCAGAAGGCAGAGAGTGTGT | ATGCCGTTCATCTTGTGATAGAG |
| Cdx2 | GCGAAACCTGTGCGAGTGGATG | CGGTATTTGTCTTTTGTCCTGGTTTTCA |
| Hand1 | CCTTCAAGGCTGAACTCAAAAA | GCGCCCTTTAATCCTCTTCT |
| Bmp2 | CGCTTCTTCTTCAATTTAAGTTCTG | AACTACTGTTTCCCAAAGCTTCCT |
| Gata2 | GCCTCTACCACAAGATGAATGG | GTCTGACAATTTGCACAACAGG |
| Otx2 | AAGTGAGTTCAGAGAGTGGAACAAG | CTCCAGATAGACACTGGAGCACT |
| Nes | AGGACCAGGTGCTTGAGAGA | TTCGAGAGATTCGAGGGAGA |
| T | CTTCAAGGAGCTAACTAACGAGATG | GTCCAGCAAGAAAGAGTACATGG |
| Gsc | AGAAGGTGGAGGTCTGGTTTAAG | GAGGACGTCTTGTTCCACTTCT |
| Pou5f1 | CTGAGGGCCAGGCAGGAGCACGAG | CTGTAGGGAGGGCTTCGGGCACTT |
| Nanog | AGGGTCTGCTACTGAGATGCTCTG | CAACCACTGGTTTTTCTGCCACCG |
| Sox2 | GCGGAGTGGAAACTTTTGTC | TATTTATAATCCGGGTGCTCCT |
