# Supplementary material for: Gata3 dosage governs primitive endoderm versus trophectoderm specification in embryonic stem cells
Source: EMBO Rep. 2026 Jul 6;27(16):4668–93. doi: 10.1038/s44319-026-00860-y (PMC13503713; doi:10.1038/s44319-026-00860-y)
Supplement: Supplementary file 6 [file 44319_2026_860_MOESM5_ESM.zip › Figure 2/2F/20250903_heatmap_target_genes.pdf]

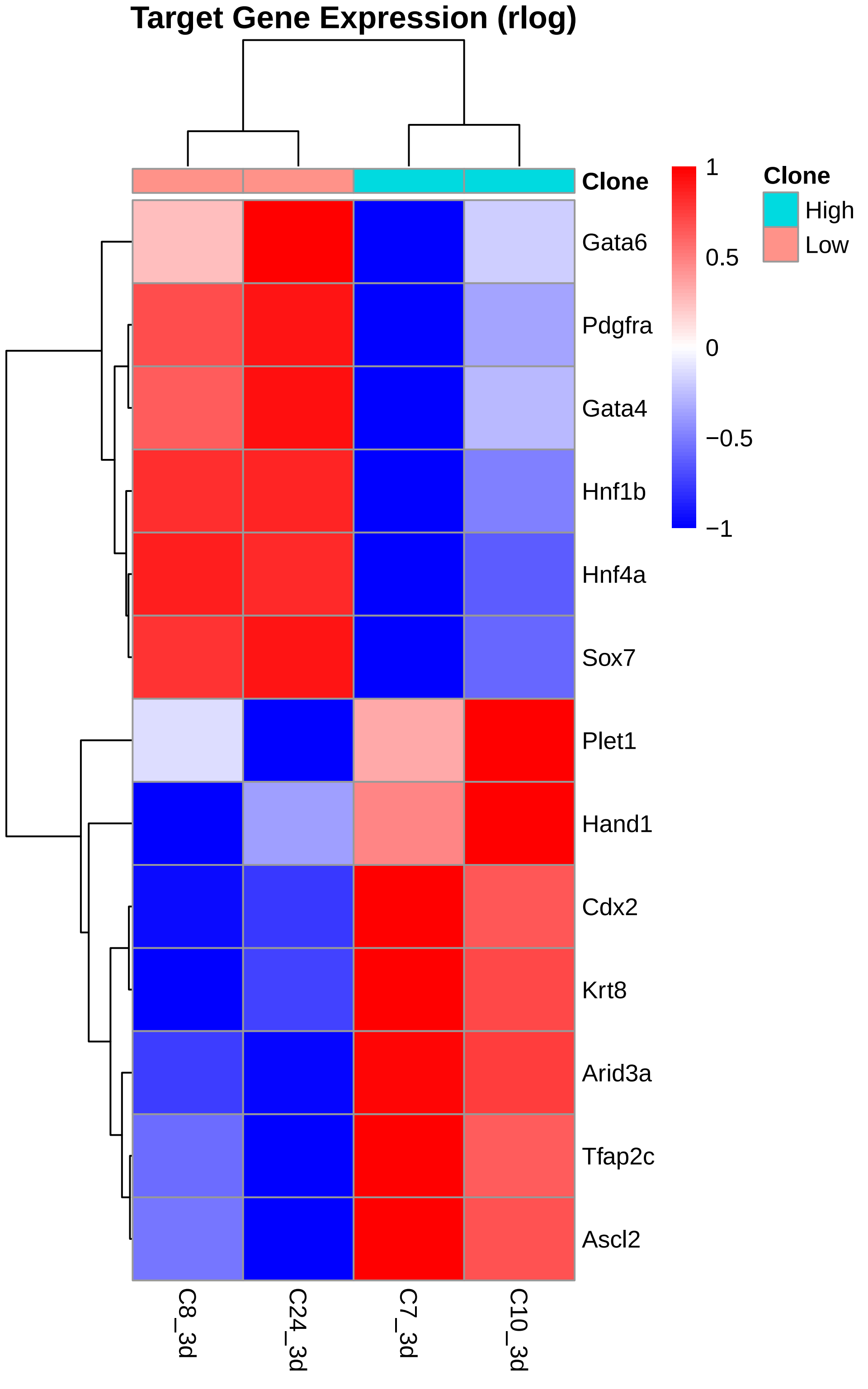
Target Gene Expression (rlog)
Clone
Gata6
Pdgfra
Gata4
Hnf1b
Hnf4a
Sox7
Plet1
Hand1
Cdx2
Krt8
Arid3a
Tfap2c
Ascl2
C8_3d
C24_3d
C7_3d
C10_3d
1
0.5
0
−0.5
−1
Clone
High
Low
