# Supplementary material for: Gata3 dosage governs primitive endoderm versus trophectoderm specification in embryonic stem cells
Source: EMBO Rep. 2026 Jul 6;27(16):4668–93. doi: 10.1038/s44319-026-00860-y (PMC13503713; doi:10.1038/s44319-026-00860-y)
Supplement: Supplementary file 6 [file 44319_2026_860_MOESM5_ESM.zip › Figure 2/2C/20250610_g3_heatmap.pdf]

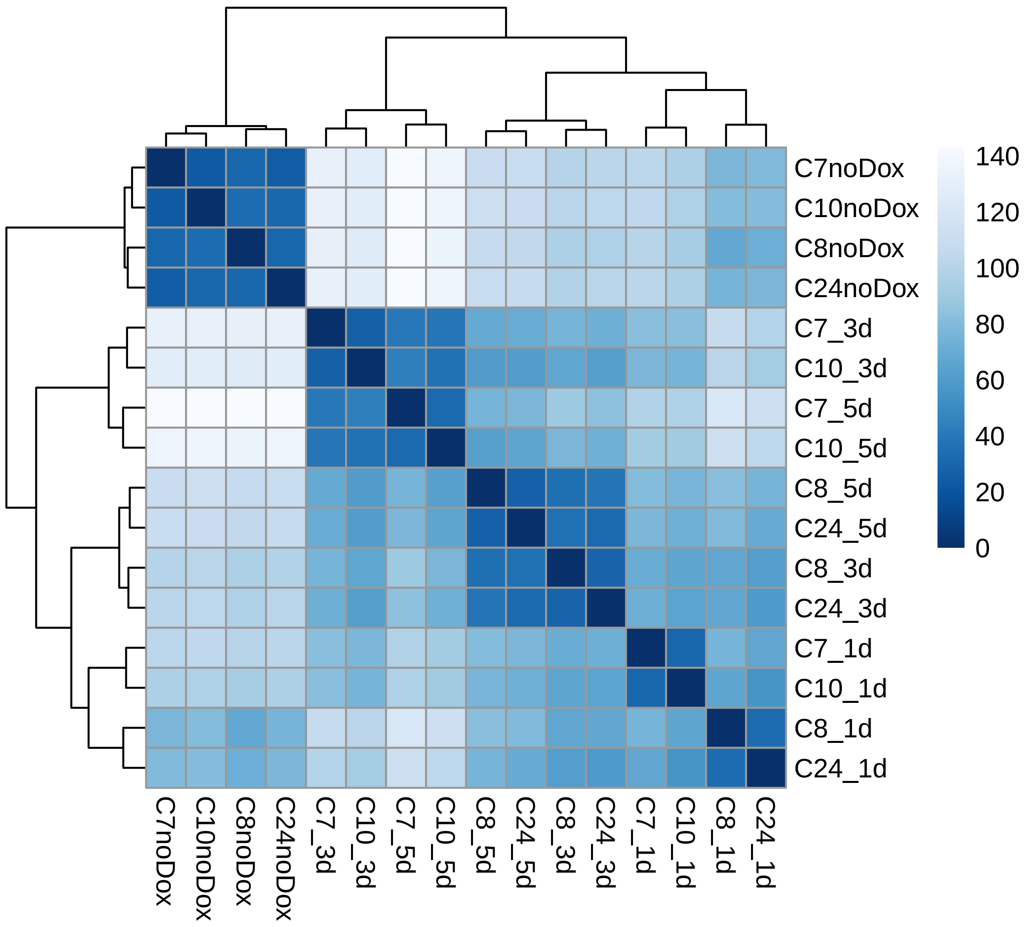

C7noDox
C10noDox
C8noDox
C24noDox
C7_3d
C10_3d
C7_5d
C10_5d
C8_5d
C24_5d
C8_3d
C24_3d
C7_1d
C10_1d
C8_1d
C24_1d
140
120
100
80
60
40
20
0
