# Supplementary material for: Gata3 dosage governs primitive endoderm versus trophectoderm specification in embryonic stem cells
Source: EMBO Rep. 2026 Jul 6;27(16):4668–93. doi: 10.1038/s44319-026-00860-y (PMC13503713; doi:10.1038/s44319-026-00860-y)
Supplement: Supplementary file 6 [file 44319_2026_860_MOESM5_ESM.zip › Figure 2/2B/Gata3_High_D3_vs_Control_plot.pdf]

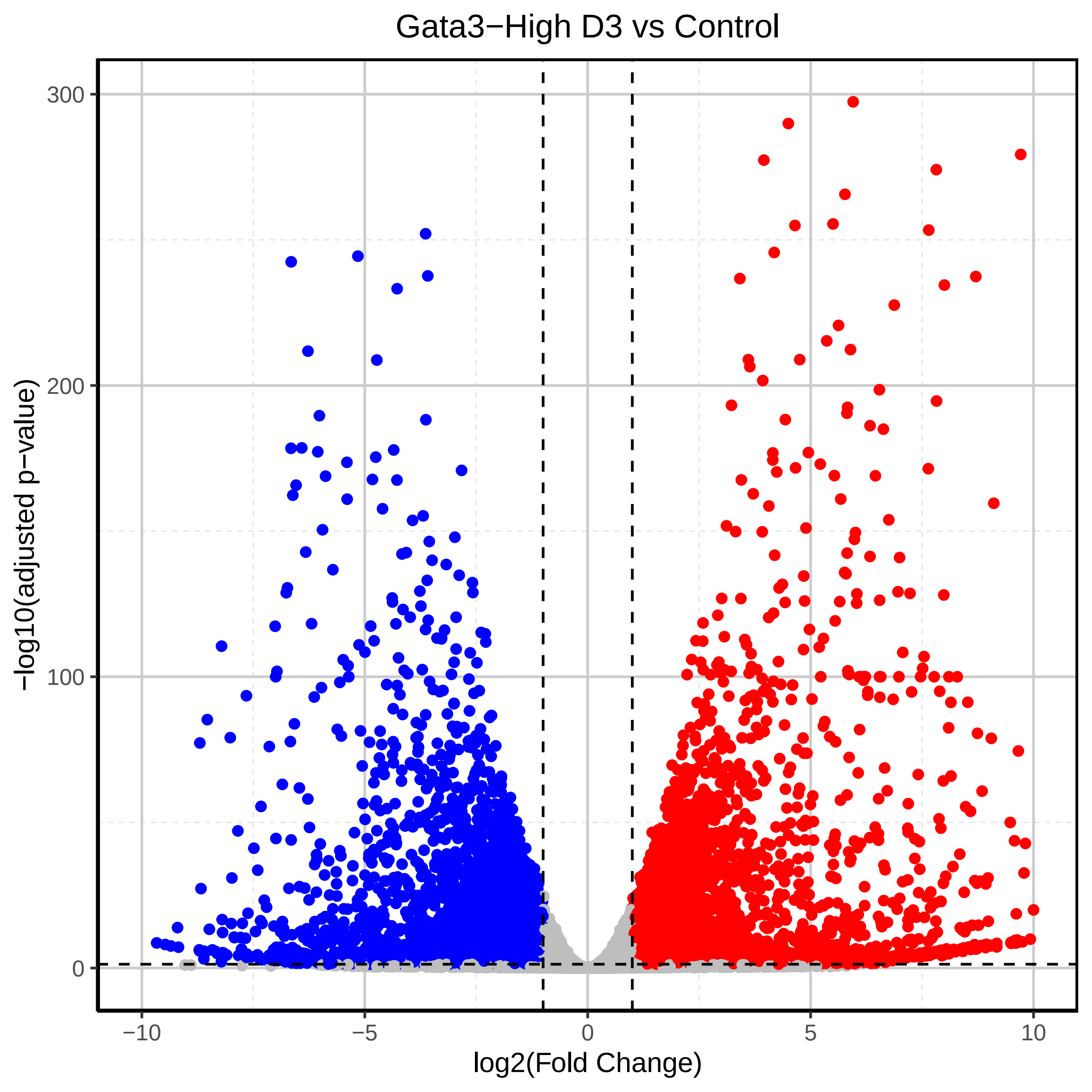
Gata3−High D3 vs Control
−log10(adjusted p−value)
300
200
100
0
−10
−5
0
5
10
log2(Fold Change)
