# Supplementary material for: Gata3 dosage governs primitive endoderm versus trophectoderm specification in embryonic stem cells
Source: EMBO Rep. 2026 Jul 6;27(16):4668–93. doi: 10.1038/s44319-026-00860-y (PMC13503713; doi:10.1038/s44319-026-00860-y)
Supplement: Supplementary file 7 [file 44319_2026_860_MOESM6_ESM.zip › Figure 3/3B/matrix_two_groups.pdf]

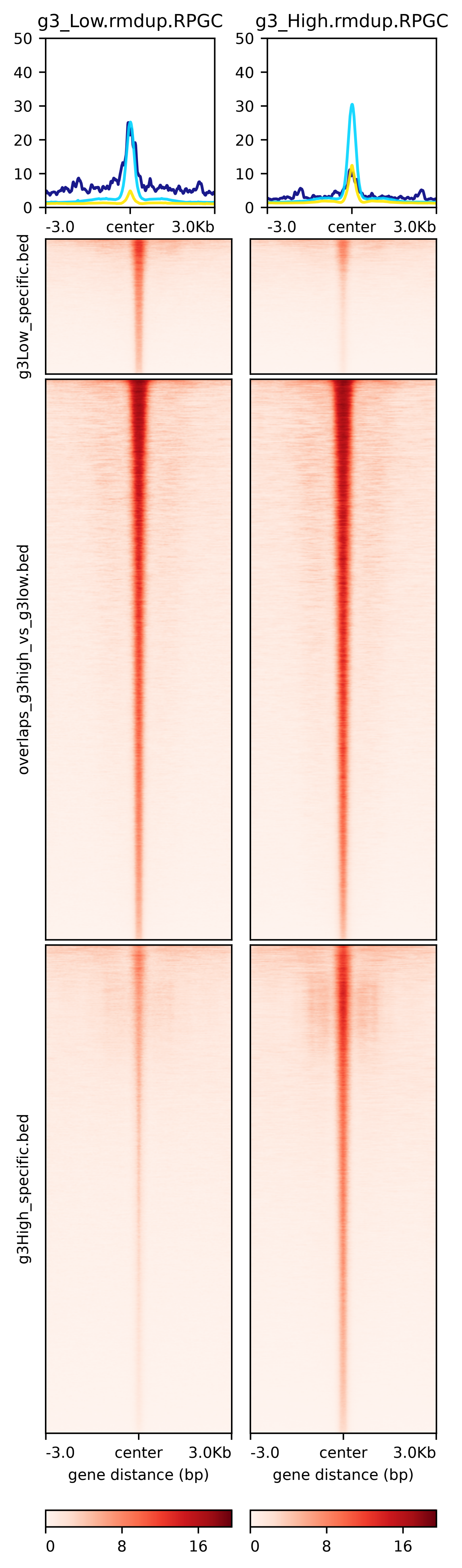
g3_Low.rmdup.RPGC
g3_High.rmdup.RPGC
50
40
30
20
10
0
-3.0
center
3.0Kb
g3Low_specific.bed
overlaps_g3high_vs_g3low.bed
g3High_specific.bed
gene distance (bp)
0
8
16
