# Supplementary material for: Gata3 dosage governs primitive endoderm versus trophectoderm specification in embryonic stem cells
Source: EMBO Rep. 2026 Jul 6;27(16):4668–93. doi: 10.1038/s44319-026-00860-y (PMC13503713; doi:10.1038/s44319-026-00860-y)
Supplement: Supplementary file 9 [file 44319_2026_860_MOESM8_ESM.zip › Figure 5/5D/Marker_Expression_Lineage.pdf]

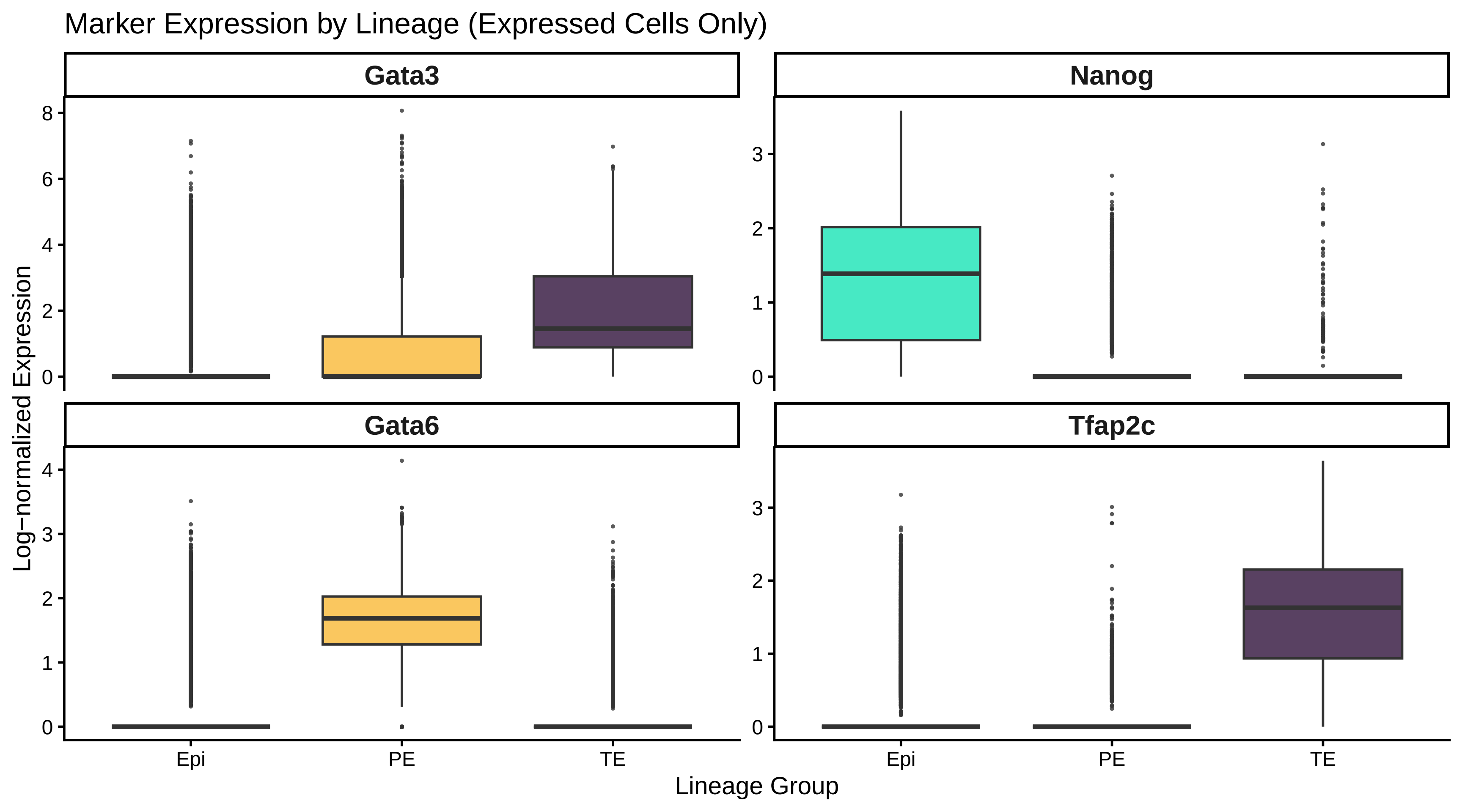

Marker Expression by Lineage (Expressed Cells Only)
Gata3
Nanog
Gata6
Tfap2c
Log−normalized Expression
0
2
4
6
8
0
1
2
3
0
1
2
3
4
0
1
2
3
Epi
PE
TE
Epi
PE
TE
Lineage Group
